# Supplementary material for: Chemical inhibition of stomatal differentiation by perturbation of the master-regulatory bHLH heterodimer via an ACT-Like domain
Source: Nat Commun. 2024 Oct 23;15:8996. doi: 10.1038/s41467-024-53214-4 (PMC11500415; doi:10.1038/s41467-024-53214-4)
Supplement: Supplementary file 1 — Supplementary Information [file 41467_2024_53214_MOESM1_ESM.pdf]

Chemical inhibition of stomatal differentiation by perturbation of the master-regulatory bHLH heterodimers via an ACT-Like domain

Ayami Nakagawa*, Krishna Mohan Sepuru*, Shu Jan Yip*, Hyemin Seo, Calvin M. Coffin, Kota Hashimoto, Zixuan Li, Yasumoto Segawa, Rie Iwasaki, Hiroe Kato, Daisuke Kurihara, Yusuke Aihara, Stephanie Kim, Toshinori Kinoshita, Kenichiro Itami, Soon-Ki Han, Kei Murakami, and Keiko U. Torii

SUPPLEMENTARY MATERIALS TABLE OF CONTENTS

Supplementary Figures

Supplementary Figure 1. Stomidazolone does not affect stomatal movement

Supplementary Figure 2. Stomidazolone inhibits stomatal differentiation in solid culture

Supplementary Figure 3. Stability of Stomidazolone in acidic/alkaline solvents and identification of the biologically inactive major degradation product

Supplementary Figure 4. Stability of Stomidazolone under our experimenal aqueous conditions

Supplementary Figure 5. A precursor of Stomidazolone, SIM3*, exhibits no effect on stomatal differentiation and MUTE-SCRM heterodimerization

Supplementary Figure 6. Stomidazolone disrupts the heterodimerization of SCRM2 with SPCH/MUTE/FAMA *in planta*

Supplementary Figure 7. Thermodynamics of the effects of Stomidazolone and its inactive analogs on SCRM-MUTE heterodimers

Supplementary Figure 8. Thermodynamics of Stomidazolone and its inactive analogs interacting with full length and ACTL domains of SCRM/SPCH/MUTE/FAMA and mutant versions of MUTE ACTL domain

Supplementary Figure 9. Heterodimerization of SCRM with Stomidazolone-resistant versions of MUTE and the ineffectiveness of biologically-inactive Stomidazolone analogs in disrupting SCRM-MUTE

Supplementary Figure 10. Docking modeling of Stomidazolone and its biologically inactive analogs

Supplementary Figure 11. Amino-acid sequence alignment of the Stomidazolone-MUTE binding interface

Supplementary Figure 12. Additional transgenic lines of the engineered Stomidazolone-resistant plants

Supplementary Tables

Supplementary Table 1. X-ray crystallography statistics

Supplementary Table 2. Thermodynamic parameters for the binding of Stomid with both bHLH transcription factors and their corresponding ACT domains

Supplementary Table 3. Kinetic- and binding constants for Stomid with both bHLH transcription factors and their corresponding ACT domains using BLI

Supplementary Table 4. Binding energies for Stomidazolone-MUTE docking simulation

Supplementary Table 5. List of oligo DNA primers and their sequences used in this study

Supplementary Table 6. List of plasmids used in this study

Supplementary Documents:

Supplementary Document 1. Chemical synthesis, X-ray crystallography, NMR and HRMS analyses

Source Data

Source Data 1. Quantitative data underlying each figure

Source Data 2. PDB Coordinates and validation files of MUTE ACTL domain- Stomidazolone binding for both racemic enantiomers

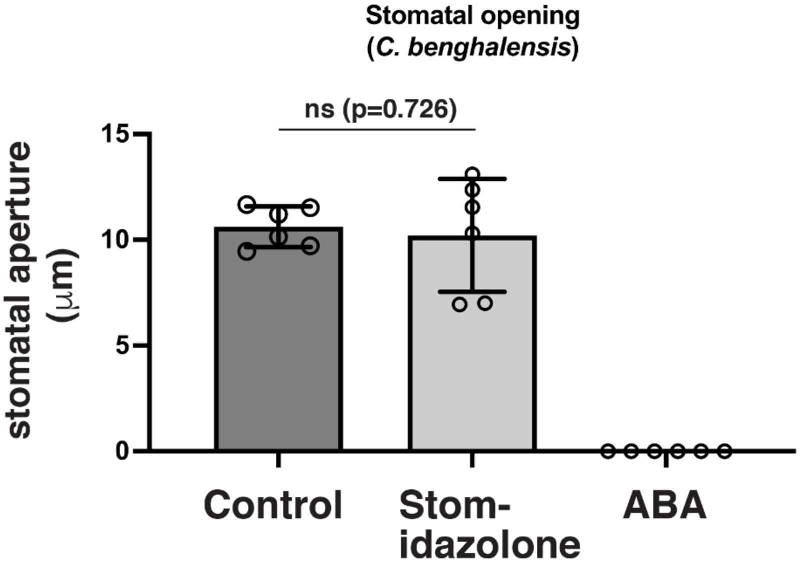

Supplementary Figure 1. Stomidazolone does not affect stomatal movement

Shown are the effects of Stomidazolone (50 μM) or positive control ABA (20 μM) on *C. benghalensis* stomatal opening induced by light (50 μmol m^{-2} s^{-1} blue light and 150 μmol m^{-2} s^{-1} red light) for 3 hours. The data are presented as mean ± SD (n = 6 biologically independent samples examined over 6-19 stomata per leaf). Paired, two-tailed Student T-test was performed, with p values indicated in the graph.

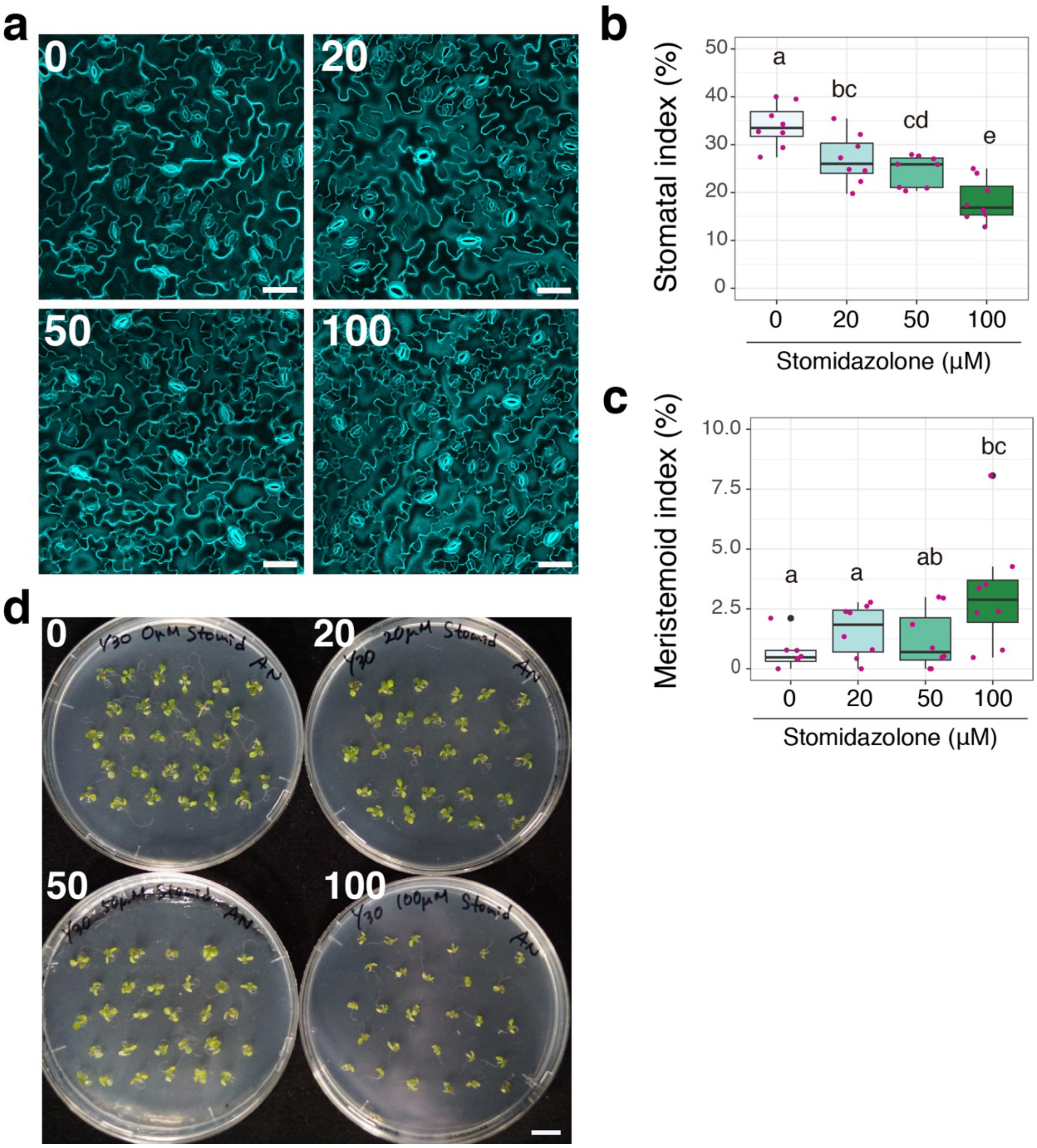

Supplementary Figure 2. Stomidazolone inhibits stomatal differentiation in solid culture
(a) Representative confocal images of 0, 20, 50 or 100 µM Stomidazolone grown on solid MS media. Stomidazolone was solidified with low-melting agarose. Scale bars, 50 µm.
(b, c) Quantitative analysis of stomatal index (%) (b) and meristemoid index (%) (c) of cotyledon abaxial epidermis from seedlings grown in the presence of 0 (mock), 20, 50, and 100 µM Stomidazolone. One-way ANOVA followed by Tukey's HSD analysis were performed for each cell type (stomata and meristemoids). Letters (a-e) indicate groups that are statistically different from other groups within each cell type. See Methods for the definition of the boxplots. n = 8.
(d) Stomidazolone does not affect growth at 20 or 50 µM, but strikingly affects growth at 100 µM. Shown are two representative 9-day-old WT seedlings, grown in the presence of 0 (mock), 20, 50, and 100 µM Stomidazolone. Scale bars, 10 mm.

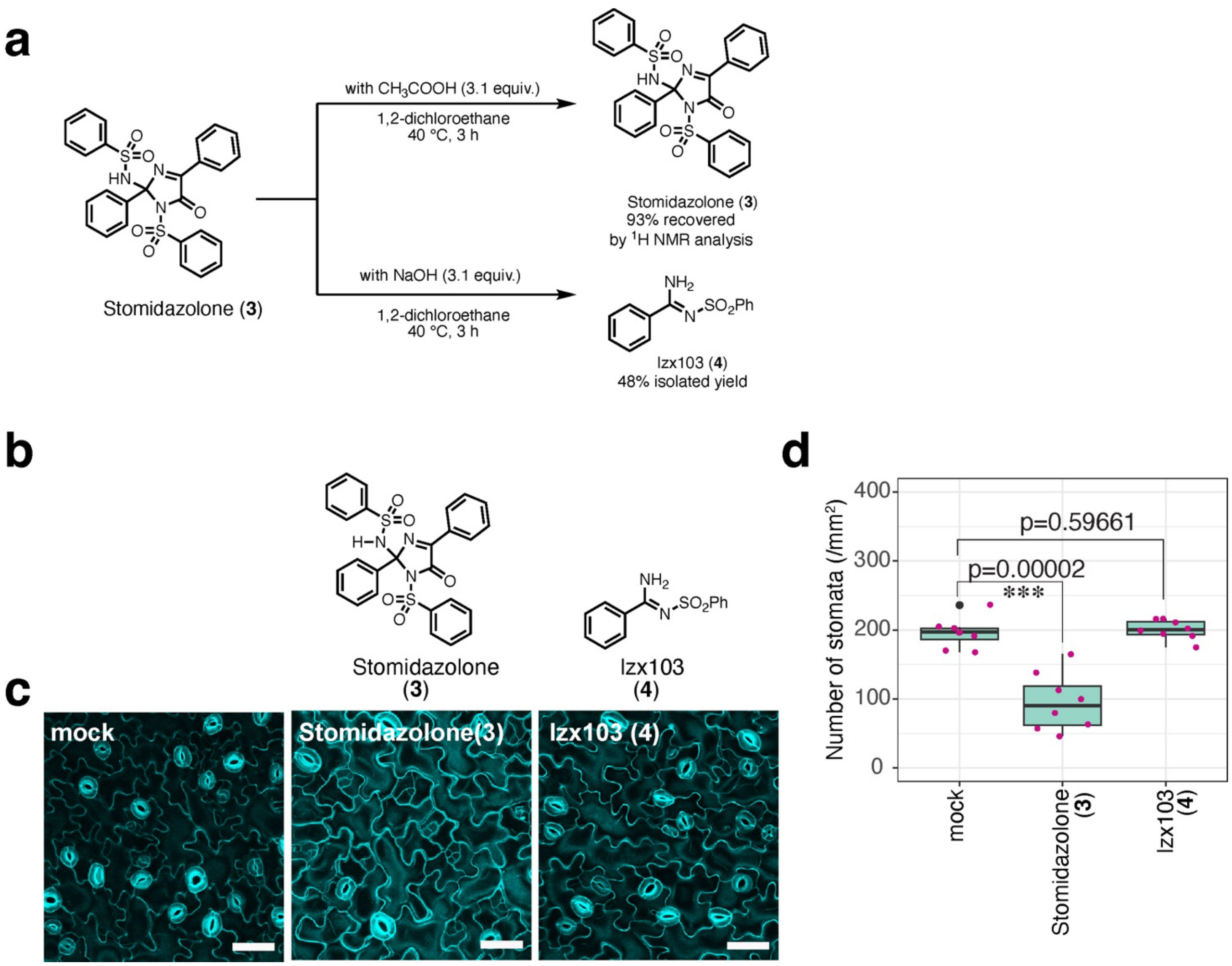

Supplementary Figure 3. Stability of Stomidazolone in acidic/alkaline solvents and identification of the biologically inactive major degradation product

(a) Summary of experiment and recovery of Stomidazolone (compound **3**) and its major degradation product lzx103 (compound **4**) in acidic (top) and alkaline (bottom) solvent conditions.
(b) Chemical structures of Stomidazolone and lzx103.
(c) Representative confocal images of cotyledon abaxial epidermis from seedlings grown with mock, or 50 μM Stomidazolone or 50 μM lzx103 grown on liquid1/2 MS media. Scale bars, 50 μm.
(d) Number of stomata per 1 mm^2 of cotyledon abaxial epidermis from seedlings grown in the presence of mock or 50 μM Stomidazolone and lzx103. Two-tailed Student's T test was performed and the exact p values are indicated. ***, $p<0.0005$. See Methods for the definition of the boxplots. n = 8.

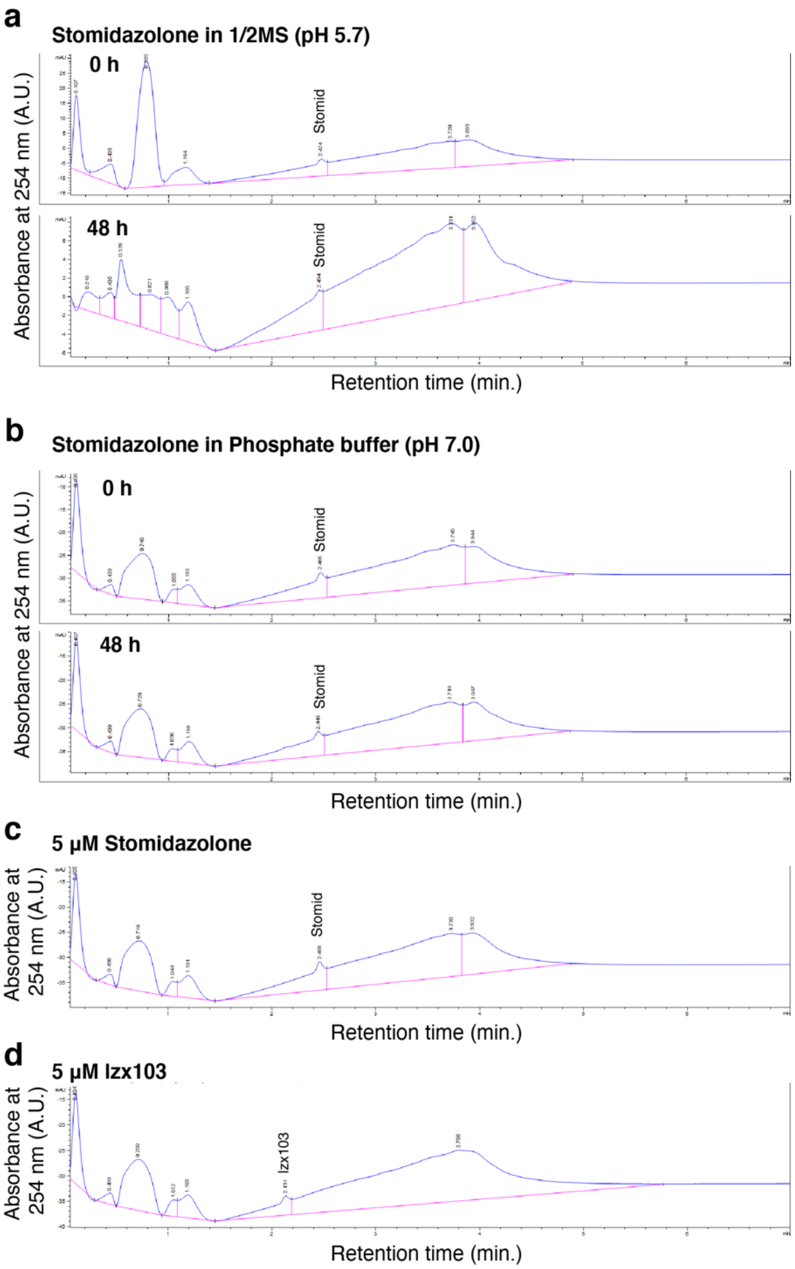

Supplementary Figure 4. Stability of Stomidazolone under our experimenal aqueous conditions

(a, b) The HPLC chromatograms of 0.5 mM Stomidazolone in 1/2MS (pH 5.7) (a), in 50 mM phosphate buffer (pH 7.0) (b) incubated for 0 or 48 hours.

(c, d) Control samples of 5 μM Stomidazolone (c) and 5 μM Izx103 (d). Stomidazolone was detected at retention time of 2.47 min. and Izx103 (compound **4**) was detected at 2.13 min. The peaks corresponding to Stomidazolone in the samples were marked as Stomid.

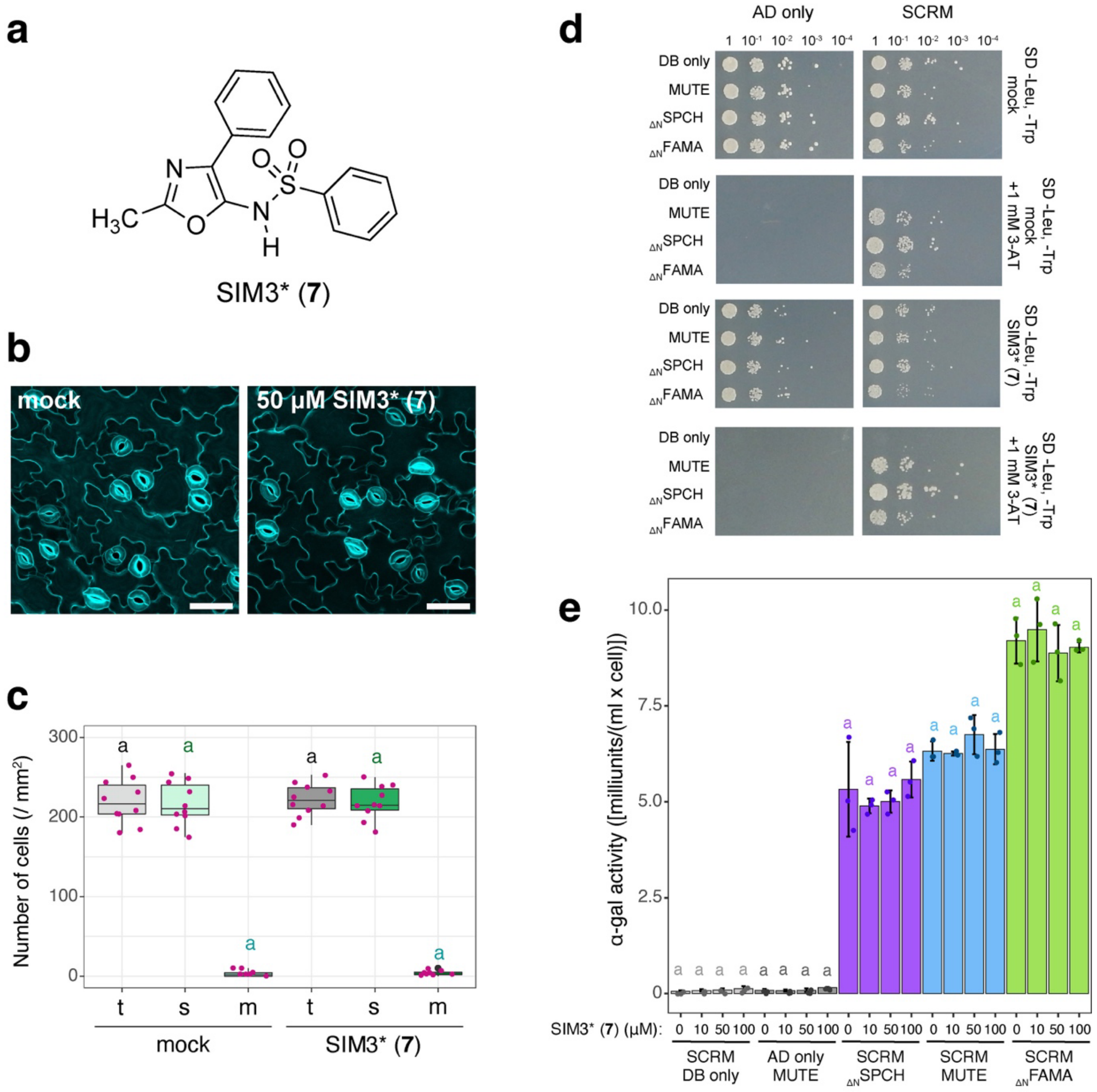

Supplementary Figure 5. A precursor of Stomidazolone, SIM3*, exhibits no effect on stomatal differentiation and MUTE-SCRM heterodimerization

(a) Structural diagram of SIM3* (compound **7**).

(b) Confocal microscopy images of representative cotyledon abaxial epidermis from 9-day-old seedlings grown in the absence (mock) or presence of SIM3* (50 μM). Scale bars, 50 μm.

(c) Quantitative analysis of stomatal (green), meristemoid (cyan), and total cell (gray) density per mm^2 of cotyledon abaxial epidermis from seedlings grown in the absence (mock) or presence of SIM3* (50 μM). One-way ANOVA followed by Tukey's HSD analysis were performed for each cell type. Letters indicate groups that are statistically different from other groups within each cell type. See Methods for the definition of the boxplots. n = 8

(d) Y2H analysis. Yeasts expressing control and pairs of stomatal bHLH proteins were spotted in 10-fold serial dilutions on appropriate selection media with or without SIM3* (50 μM). 3-AT, 3-amino-1,2,4-triazole. SIM3* does not affect the heterodimerizations.

(e) Quantitative α-galactosidase assays. Yeasts expressing control and pairs of stomatal bHLH proteins were cultured in 0, 10, 50, and 100 μM SIM3* and enzyme assays were performed. One-way ANOVA followed by Tukey's HSD analysis were performed. Letters indicate that the enzyme activities are statistically different within the group. Experiments were repeated 3 times.

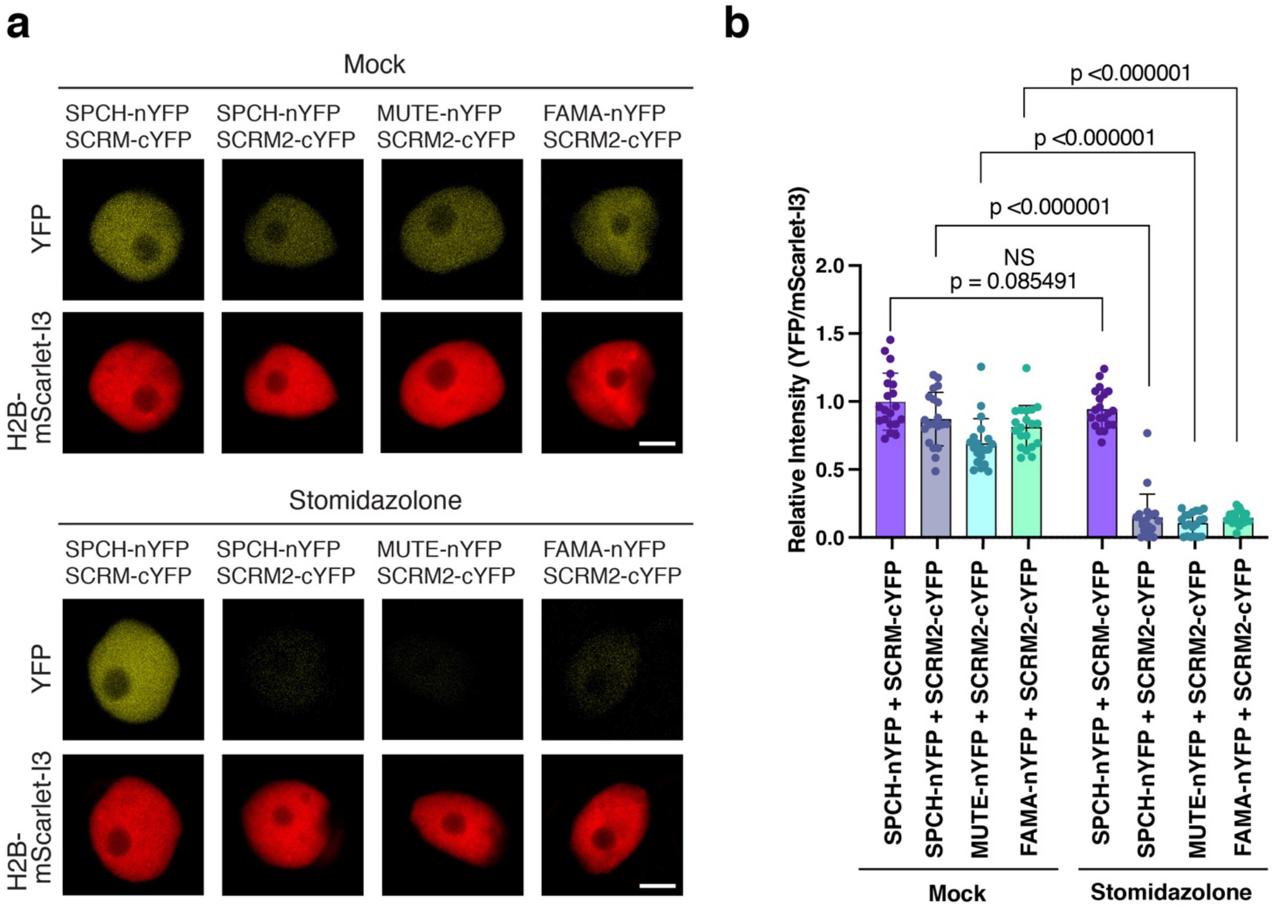

Supplementary Figure 6. Stomidazolone disrupts the heterodimerization of SCRM2 with SPCH/MUTE/FAMA *in planta*

(a) Ratiometric BiFC analysis. *N. benthamiana* leaves are infiltrated with Histone H2B (H2B)-mScarlet-I3 and a pairwise combination of SCRM2 with SPCH, MUTE, and FAMA. The SCRM-SPCH pair serves as a positive control as Stomidazoloe does not severely impact their heterodimerization (see Fig. 6c, d). Subsequently, leaf disks were treated either with mock (top) or 100 μM Stomidazolone (bottom). Shown is a representative nucleus from each combination imaged simultaneously for mScarlet-I3 (control) and YFP (interaction) signals. Scale bars, 5 μm. (b) Quantitative analysis of BiFC YFP signal intensity ratio normalized by the signal intensity of H2B-mScarlet-I3. Two-tailed Student T-test was performed for each pairwise comparison. NS, not significant. The exact p values: SPCH-SCRM2, p = 2.0514E-10; MUTE-SCRM2, p = 6.7079E-10; FAMA-SCRM2, p = 1.5976E-13. Experiments were repeated twice. Values indicate mean ± SD. n=20.

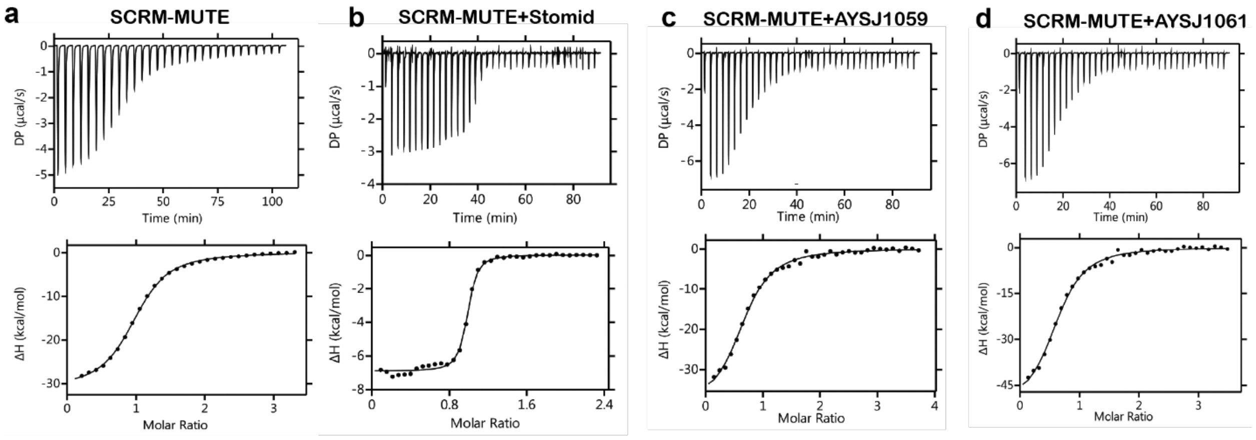

Supplementary Figure 7. Thermodynamics of the effects of Stomidazolone and its inactive analogs on SCRM-MUTE heterodimers

Shown are isotherms corresponding to the binding of SCRM to MUTE (a), SCRM to MUTE+Stomidazolone (Stomid: compound **3**) (b), SCRM to MUTE+AYSJ1059 (**5**) (c), and SCRM to MUTE+AYSJ1061 (**6**) (d). The titrations and the integrated data obtained after subtracting the heat of dilution are shown in the upper and lower panels, respectively.

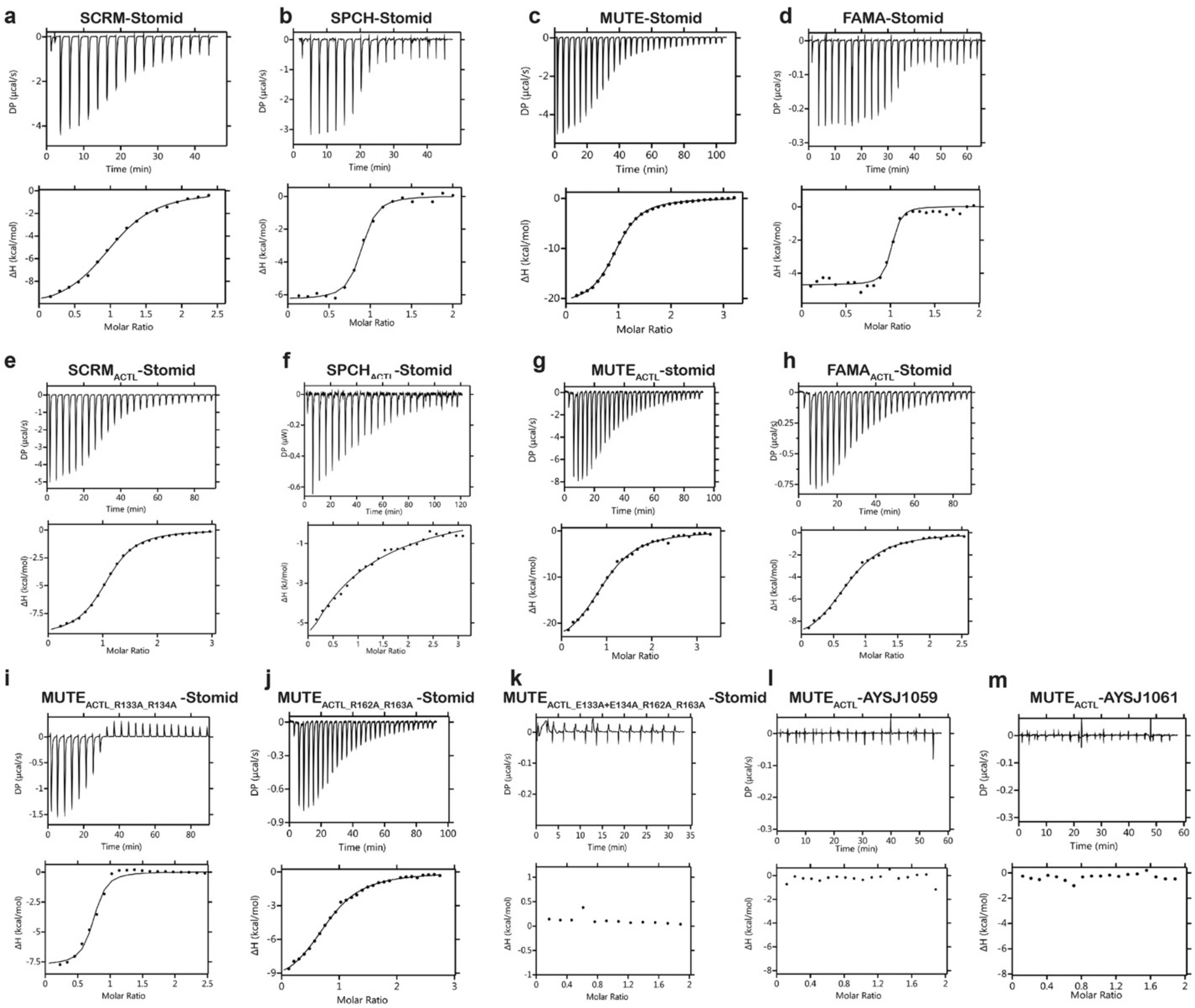

Supplementary Figure 8. Thermodynamics of Stomidazolone and its inactive analogs interacting with full length and ACTL domains of SCRM/SPCH/MUTE/FAMA and mutant versions of MUTE ACTL domain

Shown are isotherms corresponding to the binding of Stomidazolone (Stomid) to SCRM (a), Stomid to SPCH (b), Stomid to MUTE (c), Stomid to FAMA (d), Stomid to SCRM_ACTL domain (e), Stomid to SPCH_ACTL domain (f), Stomid to MUTE_ACTL domain (g), Stomid to FAMA_ACTL domain (h), Stomid to MUTE_ACTL$_{R133A_R134A}$ (i) Stomid to MUTE_ACTL$_{E162A_E163A}$ (j), Stomid to MUTE_ACTL$_{R133A_R134A_E162A_E163A}$ (k), AYSJ1059 to MUTE_ACTL (l), and AYSJ1061 to MUTE_ACTL (m). The titrations and the integrated data obtained after subtracting the heat of dilution are shown in the upper and lower panels, respectively.

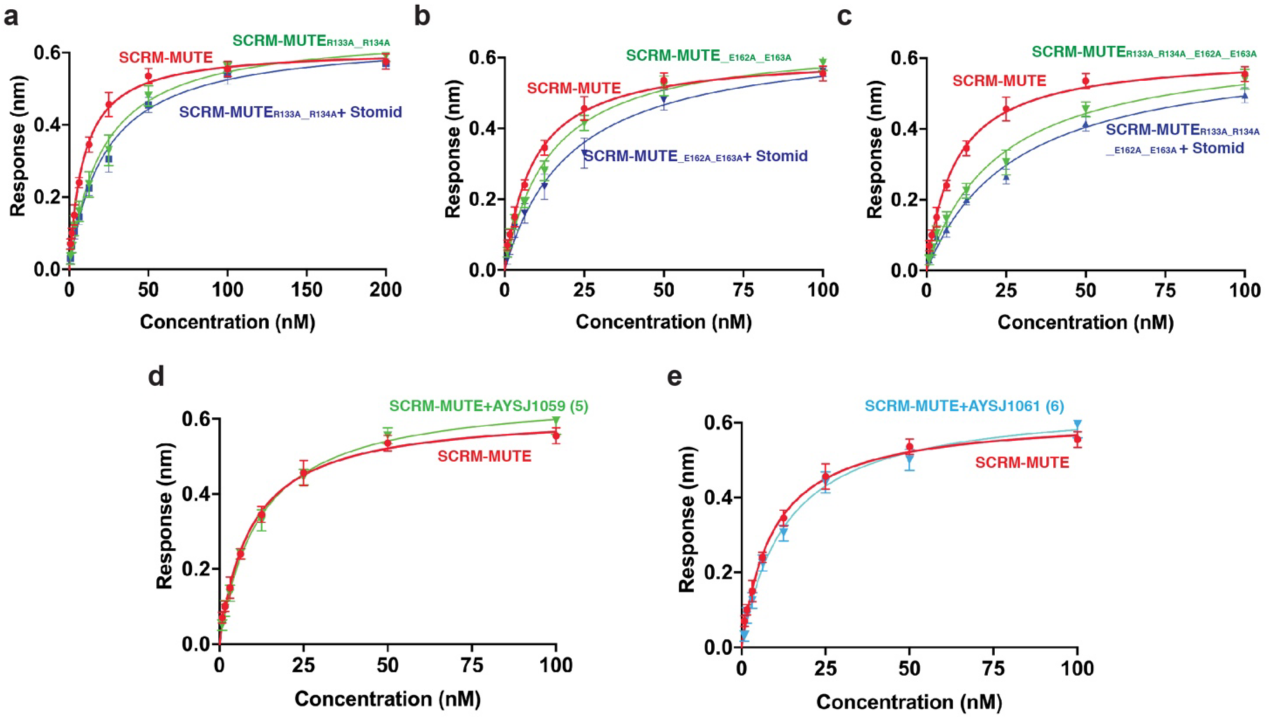

Supplementary Figure 8. Heterodimerization of SCRM with Stomidazolone-resistant versions of MUTE and the ineffectiveness of biologically-inactive Stomidazolone analogs in disrupting SCRM-MUTE

(a-c) Quantitative analysis of SCRM-MUTE interactions (red) compared with SCRM-MUTE$_{R133A_R134A}$ (a) SCRM-MUTE $_{E162A\_E163A}$ (b), and SCRM-MUTE $_{R133A\_R134A\_E162A\_E163A}$ (c) in the absence (green) and presence (blue) of Stomidazolone.

(d-e) Quantitative analysis of SCRM-MUTE interactions in the absence (red), and presence of AYSJ1059 (compound **5**, d: green) and AYSJ1061 (compound **6**, e: cyan). *In vitro* binding response curves are provided for SCRM with MUTE and with/without Stomidazolone analogues. Binding assays were performed in duplicate, and error bars indicate the standard error of the mean (SEM). The data represent the mean ± SD and are representative of two independent experiments. For all panels, the identical, representative SCRM-MUTE interaction kinetics are re-plotted for comparison. See Table S2 for the Kd values.

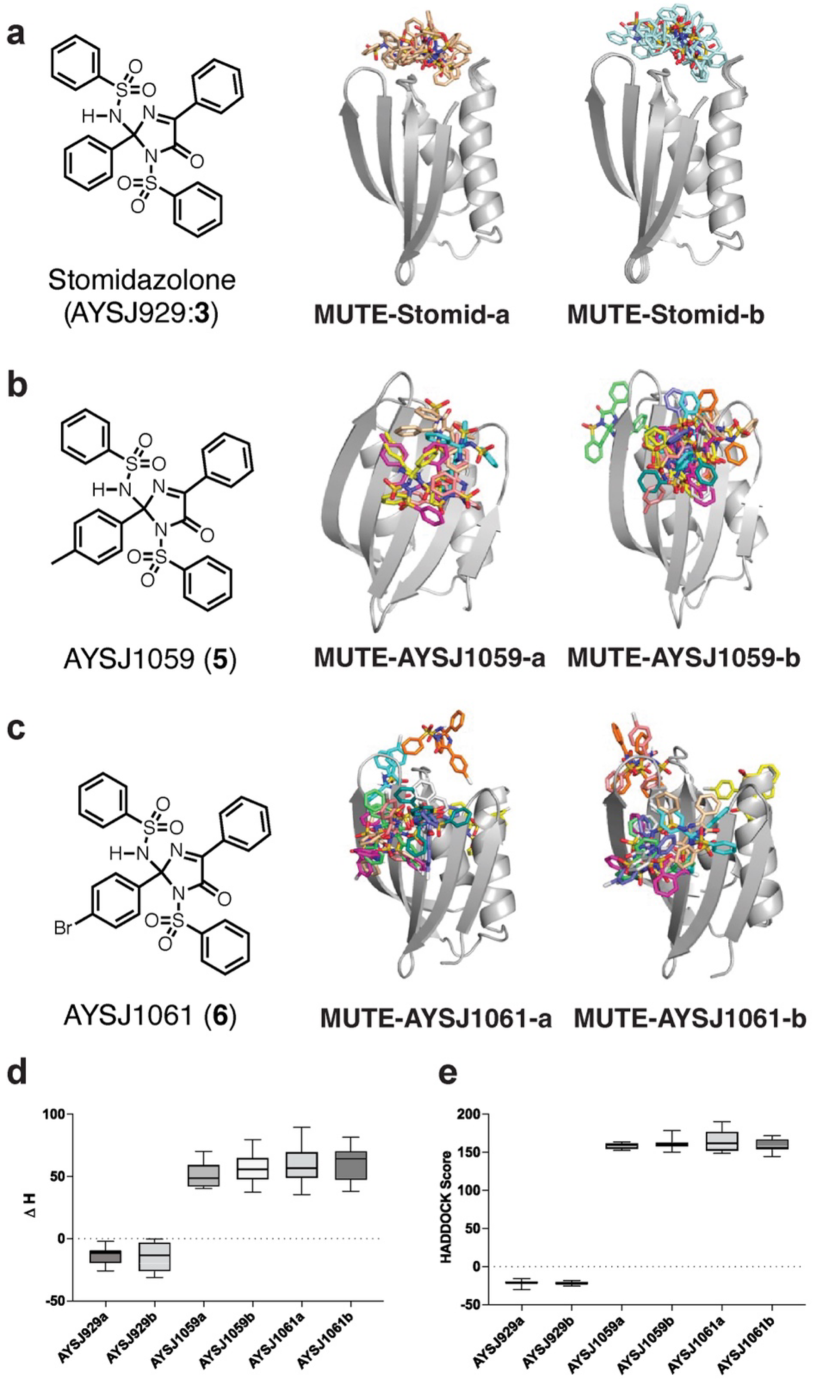

Supplementary Figure 10. Docking modeling of Stomidazolone and its biologically inactive analogs

(a-c) An overlap of 10 binding poses of MUTE-Stomidazolone (AYSJ929: **3**) (a), AYSJ1059 (**5**) (b), and AYSJ1061 (**6**) (c) from different clusters. Left, chemical structures of each compound. Middle and Right, each racemic form of the ligand models. Stomidazolone is well-clustered at the binding site, indicating specific binding.

(d, e) Comparison of the ΔH (d) and HADDOCK Score (e) suggests that stomidazolone has specific binding compared to AYSJ1059 and AYSJ1061. See Source Data S2 for raw data.

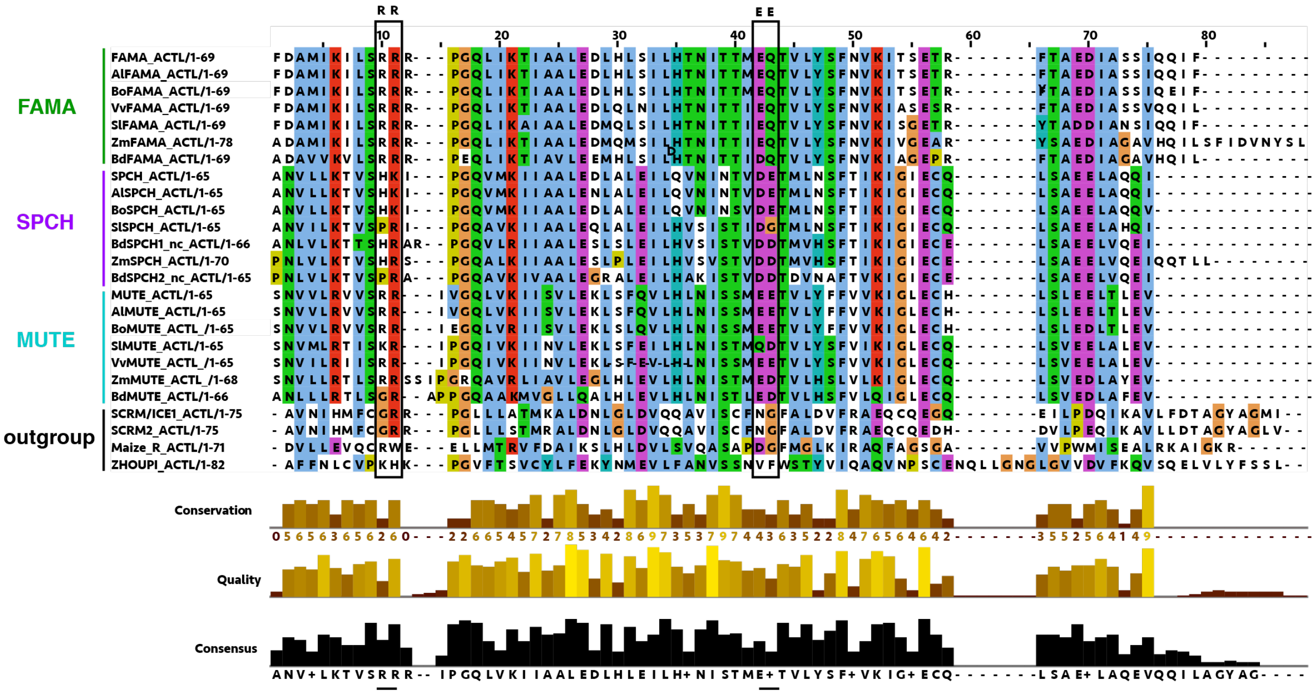

Supplementary Figure 11. Amino-acid sequence alignment of the Stomidazolone-MUTE binding interface

Sequence alignment of the ACTL domain from SPCH (purple), MUTE (cyan), and FAMA (green) and their orthologs from *Arabidopsis lyrata*, *Brassica oleracea*, *Solanum lycospersicum*, *Vitis vinifera*, *Zea mays*, and *Brachypodium distachyon* using CLUSTALW and JALVIEW. The ACTL domain from Arabidopsis SCRM, SCRM2, ZHOUPI, and Maize R are also included as an outgroup (black). The predicted and confirmed Stomidazolone binding interface of MUTE, R133 and R134, as well as E162 and E163 are highlighted in boxes and underlined below the Consensus sequence. Figure modified from Seo et al. 2022.

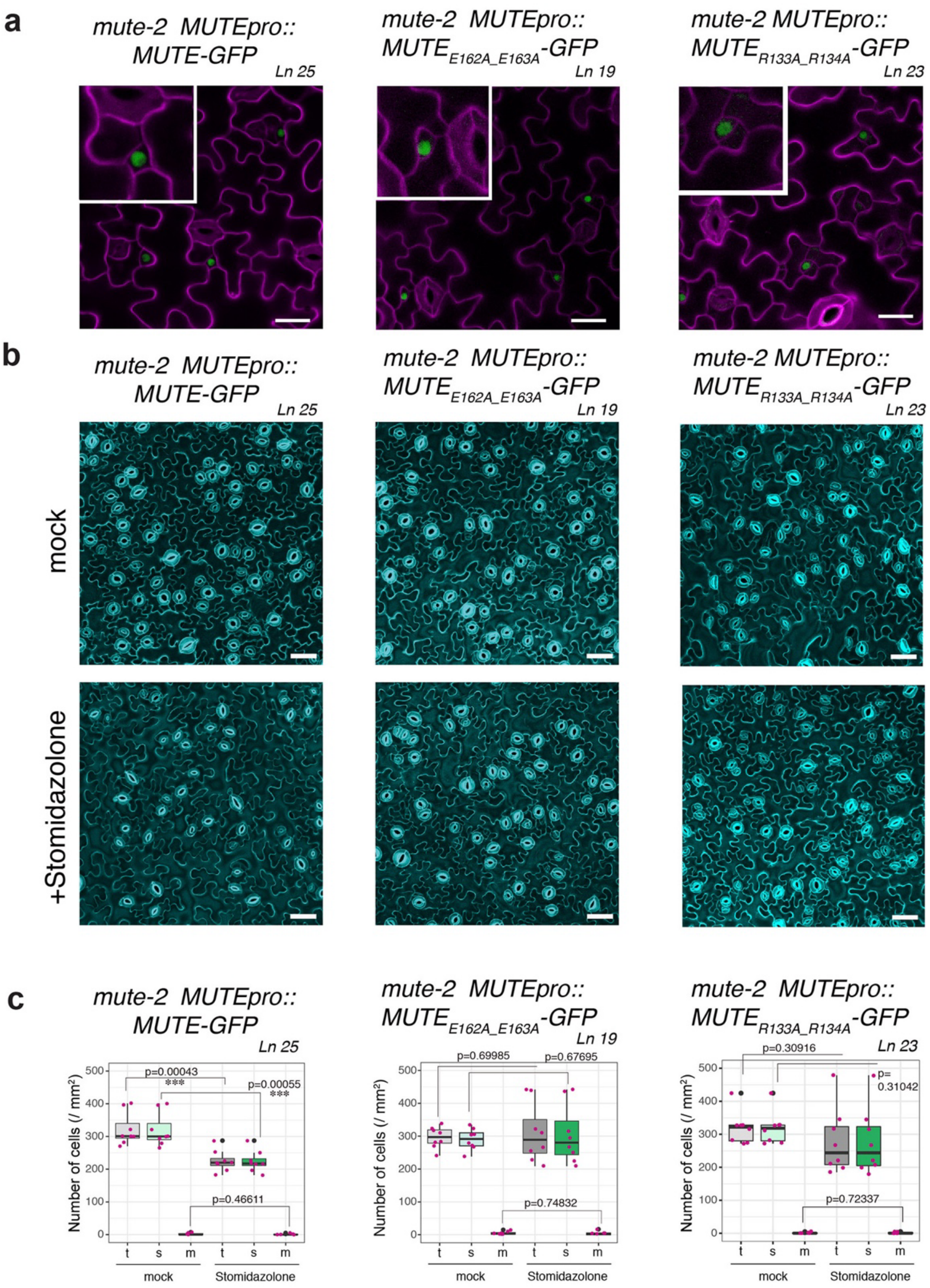

Supplementary Figure 12. Additional transgenic lines of the engineered Stomidazolone-resistant plants

(a) Site-directed mutant versions of MUTE that disrupt Stomidazolone binding are expressed normally and biologically functional. Representative cotyledon abaxial epidermis of 5-day-old *mute-2* seedlings expressing *MUTEpro::MUTE-GFP* (left), *MUTEpro::MUTE*$_{E162A\_E163A}$ *-GFP* (center), and *MUTEpro::MUTE*$_{R133A\_R134A}$ *-GFP* (right) from the additional independent transgenic

lines to those shown in Figure 7d. Insets, representative late meristemoids from each panel with MUTE-GFP signals in nucleus. Scale bars, 20 μm.

(b) MUTE mutant proteins that disrupt Stomidazolone binding confer drug resistance *in vivo*. Representative cotyledon abaxial epidermis of 9-day-old *mute-2* seedlings expressing additional independent lines of *MUTEpro::MUTE-GFP* (left), *MUTEpro::MUTE*$_{E162A\_E163A}$ *-GFP* (center), and *MUTEpro::MUTE*$_{\_R133A\_R134A}$ *-GFP* (right) with mock- (top) and 20 μM Stomidazolone for eight days (bottom). Scale bars, 50 μm.

(c) Quantitative analysis of total (t: stomata and meristemoids, gray), stomatal (s: green) or meristemoid (m: light cyan) density per mm^2 of cotyledon abaxial epidermis from seedlings of the additional independent transgenic lines grown in the presence of 0 (mock) and 20 μM Stomidazolone. Two-tailed Student's T-test was performed for each cell type (total, stomata and meristemoids) and p values are indicated. *, $p<0.05$, ***, $p<0.0005$. Experiments were repeated twice. See Methods for the definition of the boxplots. n = 8

Supplementary Table 1. Crystallographic data and structure refinement details for Stomidazolone

	**Stomidazolone**
CCDC No.	2089650
formula	$C_{27}H_{21}N_3O_5S_2$
fw	531.59
T (K)	113(2)
λ (Å)	0.71073
cryst syst	*monoclinic*
space group	$P2_1/c$
a (Å)	10.1701(3)
b (Å)	20.4139(5)
c (Å)	12.8041(4)
α (deg)	90
β (deg)	112.463(3)
γ (deg)	90
V (Å^3)	2456.58(13)
Z	4
D_{calc} (g·cm^{-3})	1.437
μ (mm^{-1})	0.262
F(000)	1104.0
cryst size (mm)	0.15 × 0.10 × 0.10
2θ range (deg)	5.27–64.134
reflns collected	29084
indep reflns/R_{int}	7038/0.0344
params	334
GOF on F^2	1.070
R_1, wR_2 [I>2σ(I)]	0.0353, 0.0881
R_1, wR_2 (all data)	0.0452, 0.0921

Supplementary Table 2. Thermodynamic parameters for the binding of Stomid with both bHLH transcription factors and their corresponding ACT domains.
The thermodynamic parameters were measured by ITC in PBS buffer, pH 7.2 buffer at 25 °C. The values reported are the means of two experiments.

	**Kd**	**n**	**ΔH**	**-TΔS**	**ΔG**
SCRM_MUTE	9.5 ± 1.6 nM	0.96	-30.5 ± 0.6	25.3 ± 0.5	-5.2 ± 0.2
SCRM_MUTE+ Stomid	900 ± 34.5 nM	0.91	-7.5 ± 0.3	5.2 ± 0.4	-2.3 ± 0.2
SCRM_MUTE+ AYSJ1059	9.6 ± 1.8 nM	1.0	-33.5 ± 0.6	27.2 ± 0.6	-6.3 ± 0.2
SCRM_MUTE+ AYSJ1061	11.4 ± 2.1 nM	1.1	-45.5 ± 0.6	35.3 ± 0.6	-10.2 ± 0.2
MUTE + Stomid	7.8 ± 1.1 μM	1.0	-20.6 ± 0.4	17.3 ± 0.4	-3.3 ± 0.2
SCRM + Stomid	71.5 ± 5.1 μM	0.94	-8.9 ± 0.2	4.1 ± 0.2	-4.8 ± 0.2
SPCH + Stomid	79.6 ± 3.6 μM	0.9	-6.2 ± 0.5	3.6 ± 0.2	-2.6 ± 0.2
FAMA + Stomid	80.4 ± 4.1 μM	1.1	-4.2 ± 0.2	2.4 ± 0.3	-1.8 ± 0.2
MUTE_ACTL + Stomid	9.8 ± 1.3 μM	1.1	-22.5 ± 0.5	14.4 ± 0.3	-8.1 ± 0.2
SCRM_ACTL + Stomid	78.4 ± 5.1 μM	0.98	-9.3 ± 0.4	6.1 ± 0.2	-3.2 ± 0.2
SPCH_ACTL + Stomid	96.8 ± 7.6 μM	0.97	-5.5 ± 0.3	3.4 ± 0.2	-2.1 ± 0.2
FAMA_ACTL + Stomid	109.6 ± 13.8 μM	1.1	-8.2 ± 0.5	4.8 ± 0.2	3.4 ± 0.2
MUTE_ACTL + AYSJ1059	ND	ND	ND	ND	ND
MUTE_ACTL + AYSJ1061	ND	ND	ND	ND	ND
$MUTE\_ACTL_{R133A\_R134A}$ + Stomid	126.6 ± 28.5 μM	1.2	-8.9 ± 0.4	5.1 ± 0.2	-3.8 ± 0.2
$MUTE\_ACTL_{E162A\_E163A}$ + Stomid	85.5 ± 5.5 μM	1.0	-7.6 ± 0.5	3.1 ± 0.3	-4.5 ± 0.2
$MUTE\_ACTL\__{R133A\_R134A\_E162A\_E163A}$ + Stomid	ND	ND	ND	ND	ND

Supplementary Table 3. Kinetic- and binding constants for Stomid with both bHLH transcription factors and their corresponding ACT domains using BLI

	k_{on} ($M^{-1}s^{-1}$)	k_{off} (s^{-1})	K_D	K_D (SS)
SCRM-MUTE	2.42 x 10^4	1.47 x 10^{-4}	6.8 ± 0.5 nM	7.5 ± 1.2 nM
SCRM-MUTE + Stomid	1.14 x 10^4	8.59 x 10^{-3}	755.2 ± 9.5 nM	856 ± 15.1 nM
SCRM-MUTE + AYSJ1059	2.13 x 10^4	1.88 x 10^{-4}	8.8 ± 0.6 nM	10.5 ± 1.2 nM
SCRM-MUTE + AYSJ1061	1.82 x 10^4	2.34 x 10^{-4}	12.8 ± 0.8 nM	12.5 ± 1.2 nM
MUTE + Stomid	4.32 x 10^3	3.95 x 10^{-2}	9.2 ± 0.3 μM	8.9 ± 1.2 μM
SCRM + Stomid	3.82 x 10^3	2.52 x 10^{-1}	65.5 ± 0.6 μM	68.5 ± 5.6 μM
SPCH + Stomid	4.32 x 10^3	3.27 x 10^{-1}	75.6 ± 0.7 μM	85.2 ± 6.8 μM
FAMA + Stomid	2.05 x 10^3	1.78 x 10^{-1}	85.1 ± 4.3 μM	78.2 ± 6.5 μM
MUTE_ACTL + Stomid	4.02 x 10^3	4.85 x 10^{-2}	12.2 ± 2.1 μM	13.1 ± 1.3 μM
SCRM _ACTL + Stomid	2.35 x 10^3	1.83 x 10^{-1}	78.2 ± 1.2 μM	76.0 ± 6.8 μM
SPCH_ACTL + Stomid	3.85 x 10^3	3.40 x 10^{-1}	88.1 ± 1.1 μM	95.2 ± 3.8 μM
FAMA _ACTL + Stomid	2.85 x 10^3	2.70 x 10^{-1}	95.2 ± 3.5 μM	105.2 ± 13.8 μM
MUTE_ACTL + AYSJ1059	3.45 x 10^3	4.70 x 10^{-1}	123.3 ± 6.5 μM	126 ± 13.5 μM
MUTE_ACTL + AYSJ1061	4.10 x 10^3	5.52 x 10^{-1}	134.6 ± 5.5 μM	139.5 ± 9.5 μM
MUTE_ACTL$_{R133A_R134A}$ + Stomid	9.87 x 10^2	1.24 x 10^{-1}	120.3 ± 5.5 μM	125 ± 12.5 μM
MUTE_ACTL$_{E162A_E163A}$ + Stomid	2.80 x 10^3	2.49 x 10^{-1}	88.3 ± 5.5 μM	85.6 ± 5.5 μM
MUTE_ACTL$_{R133A_R134A_E162A_E163A}$ + Stomid	ND	ND	ND	ND
SCRM-MUTE$_{R133A_R134A}$	1.13 x 10^4	2.56 x 10^{-4}	22.5 ± 1.8 nM	26.5 ± 1.2 nM
SCRM-MUTE $_{E162A\_E163A}$	2.90 x 10^4	3.34 x 10^{-4}	11.5 ± 2.6 nM	13.5 ± 1.6 nM
SCRM-MUTE$_{R133A_R134A_E162A_E163A}$	1.56 x 10^4	4.14 x 10^{-4}	26.5 ± 3.7 nM	32.5 ± 1.8 nM
SCRM-MUTE $_{R133A\_R134A}$ +Stomid	1.33 x 10^4	8.34 x 10^{-4}	62.5 ± 8.2 nM	56.5 ± 2.2 nM
SCRM-MUTE $_{E162A\_E163A}$ +Stomid	2.07 x 10^4	6.34 x 10^{-4}	30.5 ± 4.8 nM	33.5 ± 3.8 nM
SCRM-MUTE$_{R133A_R134A_E162A_E163A}$ + Stomid	3.48 x 10^3	3.36 x 10^{-4}	96.5 ± 12.8 nM	93.5 ± 7.8 nM

Supplementary Table 4: Binding energies for Stomidazolone-MUTE docking simulation

Racemate-a #Cluster	Haddock Score	rmsd (Å)	Nstruc	vdW (au)	Eelec (au)	BSA (Å^2)	#dH	Desol (au)
Cluster 1	-30.23	0.259	161	-25.02	-2.61	650.93	-35.024	-5.091
Cluster 2	-25.763	0.112	15	-20.18	-5.76	576.056	-13.802	-5.147
Cluster 3	-24.701	0.195	10	-21.01	10.75	654.879	9.226	-4.919
Cluster 4	-22.307	0.069	7	-18.6	-8.85	458.251	10.22	-3.276
Cluster 5	-22.249	0.141	4	-17.78	-12.21	548.473	1.642	-3.515
Cluster 6	-21.899	0.155	3	-21.27	23.11	632.448	-2.89	-2.959
**Racemate-b #Cluster**	**Haddock Score**	**rmsd (Å)**	**Nstruc**	**vdW (au)**	**Eelec (au)**	**BSA (Å^2)**	**#dH**	**Desolv (au)**
Cluster 1	-25.515	0.135	146	-20.05	-34.7	578.91	-30.404	-2.295
Cluster 2	-24.714	0.157	34	-19.02	-23.19	635.962	-26.707	-3.434
Cluster 3	-23.715	0.148	8	-17.61	-44.02	494.496	-9.051	-1.774
Cluster 4	-22.703	0.169	5	-18.18	-23.84	584.43	-2.782	-2.205
Cluster 5	-20.802	0.073	7	-15.52	-18.27	514.501	0.923	-3.529

Table includes energetic calculations with HADDOCK. rmsd: root mean square deviation; vdW–van der Waals interactions; Elec–electrostatic interactions; Desolv–desolvation energy; BSA–buried surface area; dH- free energy. a.u.–arbitrary units of energy.

Supplementary Table 5. List of oligo DNA primers and their sequences used in this study

Cloning primers

Primer ID	Primer name	Sequence (5' -> 3')
SK1	SCRM_FWD_BamH1 (1-494)	GGG GAG TCC ATG GGT CTT GAC GGA AAC
SK2	SCRM_RWD_Xho1 (1-494)	CCC CTC GAG TCA GAT CAT ACC AGC ATA
SK5	SCRM_FWD_ACT_BamH1 (405-494)	GGG GAG TCC AAA GGC CAG CAA GCT AGA
SK6	SCRM_RWD_Xho1(1-404)	CTC GAG TCA AGG ACT TGG TAA AGA AGA
SK7	MUTE_FWD_BamH1(1-202)	GGG GAG TCC ATG TCT CAC ATC GCT GTT
SK8	MUTE_RWD_Xho1(1-202)	CCC CTC GAG TTA ATT GGT AGA GAC GAT
SK9	MUTE_RWD_bHLH_Xho1(1-54)	CCC CTC GAG TTG AAC CAA TTG CTG CAA
SK10	MUTE_FWD_ACT_BamH1(114-202)	GGG GAG TCC CAT GCT AAC GTA GAA GC
SK11	SPCH_FWD_BamH1(1-364)	GGG GAG TCC ATG CAG GAG ATA ATA CCG
SK12	SPCH_RWD_Xho1(1-364)	CCC CTC GAG CTA GCA GAA TGT TTG CTG
SK15	SPCH_FWD_BamH1_ACT(285-364)	GGG GAG TCC TTG GCT GAT GTG GAA GTG
SK16	FAMA_FWD_BamH1	GCGGATCCAAGCAAATGAATGAGCATCTTCGT
SK17	FAMA_RW_Xho1	GGCTCGAGTCAAGTAAACACAATATTTCCCAGG
SK18	R133_R134 MUTE_ACTL mutant	GTA TTG AGA GTT GTC TCT GCG GCA ATC GTG GGG CAG CTC G TA
SK19	R133_R134 MUTE_ACTL mutant_com	TACGAGCTGCCCCACGATTGCCGCAGAGACAACTCTCAATAC
SK20	E162_E163_MUTE_ACTL_mutant	CTC AAT ATT AGT AGC ATG GCG GCG ACT GTC TTA TAC TTT TT C
SK21	E162_E163_MUTE_ACTL_mutant_com	GAAAAAGTATAAGACAGTCGCCGCCATGCTACTAATATTGAG
AP110	SCRM2 PENTR F	CACCATGAACAGCGACGGTGTTTGG
AP111	SCRM2 PENTR NS R	AACCAAACCAGCGTAACCTGCTGT
HS193	pAR205_R133A_F	GAGAGTTGTCTCTGGGCGAATCGTGG
HS194	pAR205_R133A_R	CCACGATTCGCCCAGAGACAACTCTC
HS195	pAR205_R134A_F	AGTTGTCTCTGCGGCCATCGTGGGGCAG
HS196	pAR205_R134A_R	CTGCCCCACGATGGCCGCAGAGACAACT
HS197	pAR205_E162A_E163A_F	CAATATTAGTAGCATGGCGGCGACTGTCTTATACTT
HS198	pAR205_E162A_E163A_R	AAGTATAAGACAGTCGCCGCCATGCTACTAATATTG
DKp665		CCTGCAGGCTCTAGAGGATCCCCCCTCAG
DKp1254		GTCGACTGTAATTGTAAATAGTAATTG
DKp2412		TACTATTTACAATTACAGTCGACATGGCACCAAGAGCCGAGAAGAA GCCCGCCGAG
DKp2599		GGCCTCTGTTGAATCCATGGCTCCACCACCTCC
DKp2600		ATGGATTCAACAGAGGCCGTGATAAAAG
DKp2601		ATCTGCAGCCGGGCGGCCGCTTCGCGATCATGAACCTCCAGAAC
DKp1251		GGCCGCCCGGCTGCAGATCGTTCAAACATTTGGCAATAAAGTTTC
DKp2598		GCTATGACCATGATTACGAATTCTCATGTTTGACAGCTTATCATCG

Supplementary Table 6. List of plasmids used in this study

Plasmid ID	Description	Insert	Backbone vector	Bacterial Resistance	Plant Resistance	References
pAR202	*MUTE* promoter casette	*MUTE* promoter	pENTR/5'-TOPO	Kan	N/A	Qi et al. 2017
pAR205	*MUTE* CDS (genomic version)	*MUTE*g no stop	pKUT612	Kan	N/A	Qi et al. 2017
pAH11	MUTEpro::MUTE-GFP	*MUTE* CDS (Genomic Version; pAR205) and *MUTE* promoter (pAR202)	R4pGWB504	Spectinomycin/Streptomycin	Hygromycin	This study
pHS133	MUTEpro::MUTE-GFP E162A E163A	MUTE E162A E163A (pHS135) and MUTE promoter (pAR202)	R4pGWB504	Spectinomycin/Streptomycin	Hygromycin	This study
pHS134	MUTEpro::MUTE-GFP R133A E134A	MUTEg_R133A_R134A (pHS136) and MUTE promoter (pAR202)	R4pGWB504	Spectinomycin/Streptomycin	Hygromycin	This study
pHS135	MUTE E162A E163A	*MUTE* CDS E162A E163A	pENTR/D-TOPO	Kan	N/A	This study
pSH136	MUTE R133A R134A	*MUTE* CDS R133A R134A	pENTR/D-TOPO	Kan	N/A	This study
pAP143	pSPYCE-SCRM	SCRM-cYFP	35S: pSPYCE	Kan	N/A	Seo et al. 2022
pHS159	pSPYCE-SCRM2	SCRM2-cYFP	35S: pSPYCE	Kan	N/A	This study
pAP159	pSPYNE-SPCH	SPCH-nYFP	35S: pSPYNE	Kan	N/A	Putarjunan et. al 2019
pAP160	pSPYNE-MUTE	MUTE-nYFP	35S: pSPYNE	Kan	N/A	Seo et al. 2022
pLW128	pSPYNE-FAMA	FAMA-nYFP	35S: pSPYNE	Kan	N/A	Seo et al. 2022
pDKv1400	35S::H2B-mScarlet-I3	H2B-mScarlet-I3	pPZP211	Spec	Kan	This study
pAP187	pGEX-4T1-SCRM	SCRM	pGEX-4T1	Amp	N/A	Putarjunan et. al 2019
pKM003	pGEX-4T1-SCRM ACT	SCRM ACTL	pGEX-4T1	Amp	N/A	Seo et al. 2022
pAP178	pGEX-4T1-ΔN SPCH	ΔN *SPCH*	pGEX-4T1	Amp	N/A	Seo et al. 2022
pKM004	pGEX-4T1-FAMA	FAMA	pGEX-4T1	Amp	N/A	Seo et al. 2022
pAH05	pGEX-4T1-MUTE	MUTE	pGEX-4T1	Amp	N/A	Seo et al. 2022
pKM005	pGEX-4T-MUTE ACT	MUTE ACTL	pGEX-4T1	Amp	N/A	Seo et al. 2022
pKM006	pGEX-4T1-SPCH ACT	SPCH ACTL	pGEX-4T1	Amp	N/A	This study
pKM007	pGEX-4T1-FAMA ACT	FAMA ACTL	pGEX-4T1	Amp	N/A	This study
pKM008	pGEX-4T1-MUTE ΔC	*MUTE* ΔC	pGEX-4T1	Amp	N/A	This study
pKM009	pGEX-4T-MUTE ACT	MUTE ACTL_R133A_R134A	pGEX-4T1	Amp	N/A	This study
pKM010	pGEX-4T-MUTE ACT	MUTE ACTL_E162A_E163A	pGEX-4T1	Amp	N/A	This study
pKM011	pGEX-4T-MUTE ACT	MUTE ACTL_R133A_R134A_E162A_E163A	pGEX-4T1	Amp	N/A	This study
pKM012	pGEX-4T-MUTE	MUTE_R133A_R134A	pGEX-4T1	Amp	N/A	This study
pKM013	pGEX-4T-MUTE	MUTE_E162A_E163A	pGEX-4T1	Amp	N/A	This study
pKM014	pGEX-4T-MUTE	MUTE_R133A_R134A_E162A_E163A	pGEX-4T1	Amp	N/A	This study
