# Supplementary material for: Chemical inhibition of stomatal differentiation by perturbation of the master-regulatory bHLH heterodimer via an ACT-Like domain
Source: Nat Commun. 2024 Oct 23;15:8996. doi: 10.1038/s41467-024-53214-4 (PMC11500415; doi:10.1038/s41467-024-53214-4)
Supplement: Supplementary file 3 — Description of additional supplementary files [file 41467_2024_53214_MOESM3_ESM.pdf]

<u>Description of Additional Supplementary Files</u>

Supplementary Data 1 - Chemical Synthesis, X-ray Crystallography, NMR and HRMS analyses

Supplementary Data 2 - PDB Coordinates and validation files of MUTE ACTL domain-Stomidazolone binding for both racemic enantiomers
