# Supplementary material for: Chemical inhibition of stomatal differentiation by perturbation of the master-regulatory bHLH heterodimer via an ACT-Like domain
Source: Nat Commun. 2024 Oct 23;15:8996. doi: 10.1038/s41467-024-53214-4 (PMC11500415; doi:10.1038/s41467-024-53214-4)
Supplement: Supplementary file 4 — Supplementary Data 1 [file 41467_2024_53214_MOESM4_ESM.pdf]

1 Supplementary Data 1. Chemical Synthesis, X-ray Crystallography, NMR and HRMS analyses

1.1 General

Unless otherwise noted, all reagents including dry solvents were obtained from commercial suppliers and used as received without further purification. 2,4-Diphenyloxazole and chloramine T trihydrate were purchased from TCI, copper(I) iodide (CuI) and silver triflate (AgOTf) from FUJIFILM Wako Pure Chemical, chloramine B dihydrate, benzamide and α-bromoacetophenone from Kanto Chemical, and 4 Å molecular sieve beads and powder from Nacalai Tesque. Unless otherwise noted, all reactions were performed with dry solvents under N_2 atmosphere in flame-dried glassware using standard vacuum-line techniques. NMR yields were determined through ^{1}H NMR with benzyl phenyl ether as the internal standard as a singlet at δ = 5.06 ppm.

Analytical thin-layer chromatography (TLC) was performed using E. Merck silica gel 60 F254 pre-coated plates (0.25 mm). Developed chromatographs were analyzed under UV lamp (254 or 365 nm) and phosphomolybdic acid/sulfuric acid solution. Flash chromatography was performed with E. Merck silica gel 60 (230–400 mesh). Silica-gel column chromatography was performed on an Isolera Spektra instrument equipped with a Biotage SNAP Ultra 10 g cartridge for 0.2 mmol scale reactions. The high-resolution mass spectra were recorded on Thermo Fisher Scientific Exactive Plus (ESI) with Thermo Scientific Dionex UltiMate 3000 Series UHPLC, Agilent Technologies 6130 Quadrupole Liquid Chromatography/Mass Spectrometry (LC-MS), or JEOL JMS-T100LC spectrometer (ESI). Nuclear magnetic resonance (NMR) spectra were recorded on a JNM-ECA-600 (^{1}H 600 MHz, ^{13}C 150 MHz) spectrometers or a JNM-ECX-500 (^{1}H 500 MHz, ^{13}C 126 MHz) spectrometer. Chemical shifts for ^{1}H NMR are expressed in parts per million (ppm) relative to residual $CHCl_3$ in $CDCl_3$ (δ 7.26 ppm), and for ^{13}C NMR in ppm relative to $CDCl_3$ (δ 77.2 ppm). Data are reported as follows: chemical shift, multiplicity (s = singlet, d = doublet, dd = doublet of doublets, t = triplets, dt = doublet of triplets, td = triplet of doublets, tt = triplet of triplets, q = quartets, m = multiplets, brs = broad singlet), coupling constant (Hz), and integration.

1.2 Synthesis of 4-(4-fluorophenyl)-2-phenyloxazole

The synthesis of oxazole[1a] was carried out based on literature.

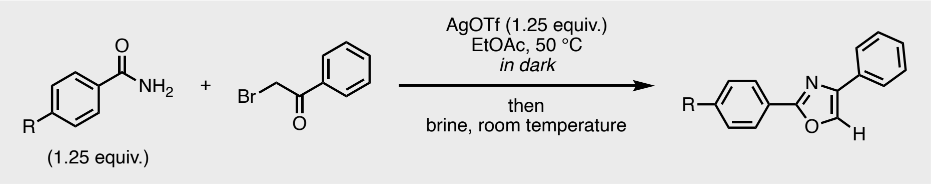

Method A: Benzamide (3.75 mmol, 1.25 equiv.), 2-bromoacetophenone (3.0 mmol), and AgOTf (3.75 mmol, 1.25 equiv.) were added to a large screw-capped tube with a stirring bar. The tube was filled with nitrogen by employing the usual Schlenk technique (evacuate-refill cycle). Anhydrous ethyl acetate (4.2 mL) was added to the tube before wrapping with aluminium foil to prevent ambient light from penetrating through, and stirred at 50 °C for 4 h. The reaction mixture was cooled to room temperature and brine was added to the flask and stirred at room temperature for 18 h. Resulting silver salt was removed by filtration and the filtrate was washed with water, aqueous $NaHCO_3$, 1 M aqueous HCl, and water. The resultant organic layer was dried over Na_2SO_4 and concentrated *in vacuo.* Purification by flash chromatography on silica gel afforded the desired product.

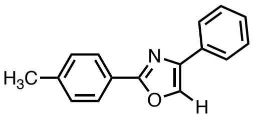

2-(4-Methylphenyl)-4-diphenyloxazole: The title compound was synthesized according to the General Procedure (**Method A**) from 4-methylbenzamide (508 mg, 3.76 mmol), 2-bromoacetophenone (599 mg, 3.01 mmol), and AgOTf (960 mg, 3.74 mmol). Purified by flash chromatography on silica gel (hexane/EtOAc = 95:5) afforded the corresponding oxazole (138 mg, 0.59 mmol, 19%) as a white solid. The product is known and the spectroscopic properties are consistent with the data available in the literature[1b].

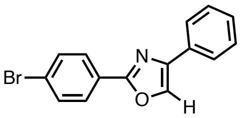

2-(4-Bromophenyl)-4-diphenyloxazole: The title compound was synthesized according to the General Procedure (**Method A**) from 4-bromobenzamide (749 mg, 3.74 mmol), 2-bromoacetophenone (596 mg, 2.99 mmol), and AgOTf (967 mg, 3.81 mmol). Purified by flash chromatography on silica gel (hexane/EtOAc = 95:5) and GPC afforded the corresponding oxazole (178 mg, 0.59 mmol, 20%) as a white solid. ^{1}H NMR ($CDCl_3$ 500 MHz) δ 7.33–7.36

(m, 1H), 7.44 (t, J = 7.5 Hz, 2H), 7.62 (dt, J = 8.5, 2.0 Hz, 2H), 7.80–7.82 (m, 2H), 7.97–8.00 (m, 3H); ^{13}C NMR ($CDCl_3$ 126 MHz) δ 125.0, 125.8, 126.5, 128.1, 128.4, 128.9, 131.0, 132.2, 133.8, 142.4, 161.2; HRMS (ESI, positive): *m/z* = 300.0020 calcd for $C_{15}H_{11}BrNO$: 300.0024 $[M + H]^+$.

1.3 Synthesis of Stomidazolone (AYSJ929)

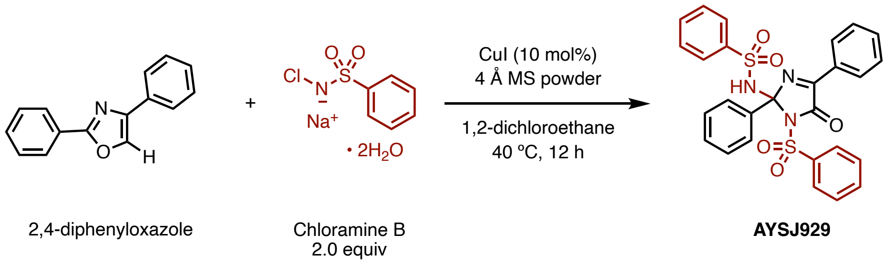

Synthesis of Stomidazolone (AYSJ929): 4 Å Molecular sieve powder (500 mg) was added into a large screw-capped tube and heat-dried *in vacuo* for 1 h. 2,4-Diphenyloxazole (111 mg, 0.50 mmol), chloramine B dihydrate (250 mg, 1.0 mmol, 2.0 equiv) and CuI (9.5 mg, 50 μmol, 10 mol%) were added into a the tube containing a magnetic stirring bar in open air. The tube was evacuated and refilled with N_2 gas following the usual Schlenk technique. Anhydrous 1,2-dichloroethane (5.0 mL, 0.10 M) was added to the tube *via* the rubber top and its capped wrapped with parafilm. The reaction mixture was stirred and heated at 40 ºC for 12 h. The mixture was then cooled to room temperature, and the crude mixture was filtered through a pad of silica gel topped with Na_2SO_4 in a short column and concentrated *in vacuo*. Purification by flash chromatography on silica gel (hexane/EtOAc = 4:1) followed by recrystallization from CH_2Cl_2/MeOH provided Stomidazolone (AYSJ929) in 42% yield (113 mg, 0.21 mmol) as colorless crystals.

Characterization of Stomidazolone (AYSJ929)

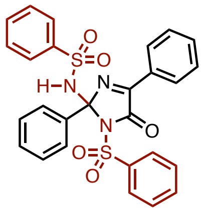

Stomidazolone (AYSJ929): ^{1}H NMR ($CDCl_3$) δ 6.75 (brs, 1H), 7.36–7.40 (m, 5H), 7.45 (t, J = 7.2 Hz, 2H), 7.52–7.54 (m, 3H), 7.58–7.61 (m, 3H), 7.70–7.73 (m, 1H), 7.78 (d, J = 8.4 Hz,

2H), 8.11 (d, *J* = 8.4 Hz, 2H), 8.22 (d, *J* = 7.8 Hz, 2H); ^{13}C NMR ($CDCl_3$) δ 97.10, 126.12, 127.72, 128.35, 128.75, 128.98, 129.05, 129.38, 129.46, 129.92, 130.29, 133.24, 133.52, 135.17, 136.53, 136.81, 141.65, 160.42, 160.54; HRMS (ESI-MS, positive): *m/z* = 554.0813. calcd for $C_{27}H_{21}N_3S_2O_5Na$: 554.0815 $[M + Na]^+$.

Synthesis of Stomidazolone analogs

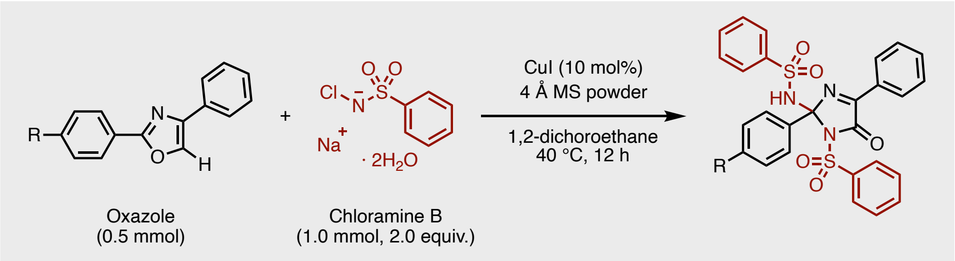

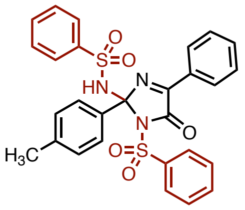

35%
AYSJ1059

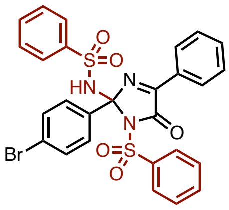

24%
AYSJ1061

Method B: 4 Å Molecular sieve powder (500 mg) was added into a large screw-capped tube with a stirring bar. The tube was heat-dried *in vacuo* for 10 minutes. Oxazole (0.5 mmol, 1.0 equiv.), chloramine salt (1.0 mmol, 2.0 equiv.), and CuI (10 mol%) were added into the tube in open air. The tube was filled with nitrogen by employing the usual Schlenk technique (evacuate-refill cycle). Anhydrous 1,2-dichloroethane (5.0 mL, 0.10 M) was added to the tube and the reaction mixture was stirred at 40 ºC for 12 h. The mixture was then cooled to room temperature, and the crude mixture was filtered through a short pad of silica gel, celite, and Na_2SO_4 and concentrated *in vacuo*. Purification by flash chromatography on silica gel provided the desired product.

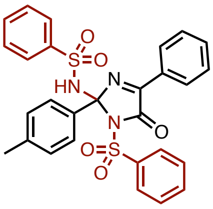

AYSJ1059: The title compound was synthesized according to General Procedure (**Method B**) from 2-(4-methylphenyl)-4-diphenyloxazole (118 mg, 0.50 mmol), Chloramine B dihydrate (250 mg, 1.0 mmol), and CuI (9.2 mg, 0.048 mmol). Purified by flash chromatography on silica gel (hexane/EtOAc = 75:25) afforded AYSJ1059 (95.0 mg, 0.17 mmol, 35%) as a white solid. ^{1}H NMR ($CDCl_3$ 500 MHz) δ 2.34 (s, 3H), 6.74 (s, 1H), 7.18 (d, *J* = 8.0 Hz, 2H), 7.35–7.46 (m, 6H), 7.52 (t, *J* = 8.0 Hz, 1H), 7.57–7.62 (m, 3H), 7.71 (t, *J* = 8.0 Hz, 1H), 7.76–7.78 (m, 2H), 8.09–8.11 (m, 2H), 8.22–8.24 (m, 2H); ^{13}C NMR ($CDCl_3$ 126 MHz) δ 21.3, 97.1, 125.9, 127.6, 128.3, 128.7, 128.9, 129.0, 129.4, 129.9, 130.0, 133.1, 133.4, 133.5, 135.1, 136.8, 140.5,

141.6, 160.1, 160.5; HRMS (APCI, positive): *m/z* = 568.0981 calcd for $C_{28}H_{23}N_3NaO_5S_2$: 568.0977 $[M + Na]^+$.

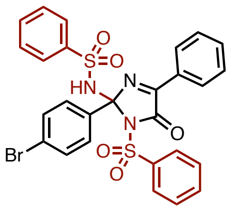

AYSJ1061: The title compound was synthesized according to General Procedure (**Method B**) from 2-(4-bromophenyl)-4-diphenyloxazole (151 mg, 0.50 mmol), Chloramine B dihydrate (250 mg, 1.0 mmol), and CuI (9.9 mg, 0.052 mmol). Purified by flash chromatography on silica gel (hexane/EtOAc = 80:20 to 75:25) afforded AYSJ1061 (72.2 mg, 0.12 mmol, 24%) as a white solid. ^{1}H NMR ($CDCl_3$ 500 MHz) δ 6.74 (s, 1H), 7.35–7.46 (m, 6H), 7.50–7.56 (m, 3H), 7.58–7.64 (m, 3H), 7.72–7.75 (m, 3H), 8.10 (d, *J* = 8.0 Hz, 2H), 8.24 (d, *J* = 8.0 Hz, 2H); ^{13}C NMR ($CDCl_3$ 126 MHz) δ 96.6, 124.7, 127.6, 127.9, 128.1, 128.8, 129.0, 129.02, 129.05, 129.9, 132.5, 133.3, 133.7, 135.3, 135.8, 136.5, 141.4, 160.3, 160.6; HRMS (APCI, positive): *m/z* = 631.9944 calcd for $C_{27}H_{20}BrN_3NaO_5S_2$: 631.9925 $[M + Na]^+$.

Stability test of Stomidazolone (AYSJ929)

Stomidazolone under acidic conditions

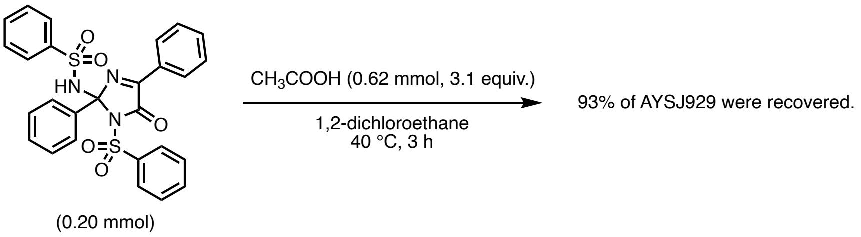

Stomidazolone (106 mg, 0.20 mmol) was added into a large screw-capped tube with a stirring bar. The tube was filled with nitrogen by employing the usual Schlenk technique (evacuate-refill cycle). Anhydrous 1,2-dichloroethane (4.0 mL) and CH_3COOH (37.0 mg, 0.62 mmol, 3.1 equiv.) were added to the tube and the reaction mixture was stirred at 40 °C for 3 h. The resulted mixture was treated with a short pad of silica gel and celite. The resultant was concentrated *in vacuo*. Stomidazolone (93% ^{1}H NMR yield using $C_2H_2Cl_4$ as the internal standard) was remained in the crude mixture.

Stomidazolone under basic conditions

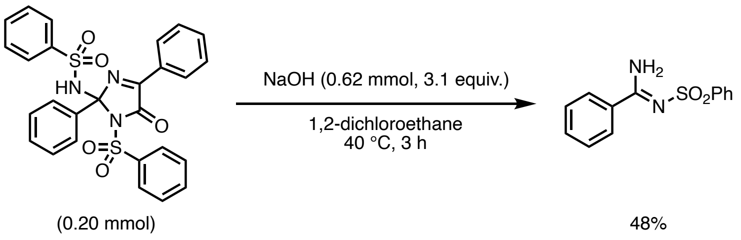

Stomidazolone (107 mg, 0.20 mmol) was added into a large screw-capped tube with a stirring bar. The tube was filled with nitrogen by employing the usual Schlenk technique (evacuate-refill cycle). Anhydrous 1,2-dichloroethane (4.0 mL) and aqueous NaOH (3.1 M, 0.20 mL) were added to the tube and the reaction mixture was stirred at 40 °C for 3 h. Saturated aqueous NH_4Cl was added to the crude mixture and extracted with CH_2Cl_2 for 3 times. The resulting organic layer was dried over Na_2SO_4 and concentrated *in vacuo*. Purification by GPC afforded lzx103 (25.3 mg, 0.097 mmol, 48%) as a white solid. ^{1}H NMR ($CDCl_3$ 500 MHz) δ 6.37 (s, 1H), 7.43 (t, *J* = 8.0 Hz, 2H), 7.48–7.51 (m, 2H), 7.53–7.57 (m, 2H), 7.78–7.80 (m, 2H), 8.00–8.02 (m, 2H), 8.36 (s, 1H); ^{13}C NMR ($CDCl_3$ 126 MHz) δ 126.6, 127.5, 128.9, 129.0, 132.5, 133.0, 133.4, 142.2, 163.0; HRMS (ESI, positive): *m/z* = 283.0530 calcd for $C_{13}H_{12}N_2NaO_2S$: 283.0517 $[M + Na]^+$.

2 X-ray Crystallographic Analysis

Details of the crystal data and a summary of the intensity data collection parameters for Stomidazolone (AYSJ929 (compound **3**), originally numbered as AYSJ1167-1) are listed in Supplementary Table 1. A suitable crystal was mounted with mineral oil on a MiTeGen MicroMeshes and transferred to the goniometer of the kappa goniometer of a RIGAKU XtaLAB Synergy-S system with 1.2 kW MicroMax-007HF microfocus rotating anode (Graphite-monochromated Mo Kα radiation (λ = 0.71073 Å)) and HyPix6000HE hybrid photon-counting detector. Cell parameters were determined and refined, and raw frame data were integrated using CrysAlis^{Pro} (Agilent Technologies, 2010). The structures were solved by direct methods with SHELXT[2] and refined by full-matrix least-squares techniques against F^2 (SHELXL-2018/3)[3] by using Olex2 software package.[4] The intensities were corrected for Lorentz and polarization effects. The non-hydrogen atoms were refined anisotropically. Hydrogen atoms were placed using AFIX instructions. CCDC 2089650 contains the supplementary crystallographic data for this paper. These data can be obtained free of charge from The Cambridge Crystallographic Data Centre via www.ccdc.cam.ac.uk/data_request/cif. CIF/PLATON report is provided.

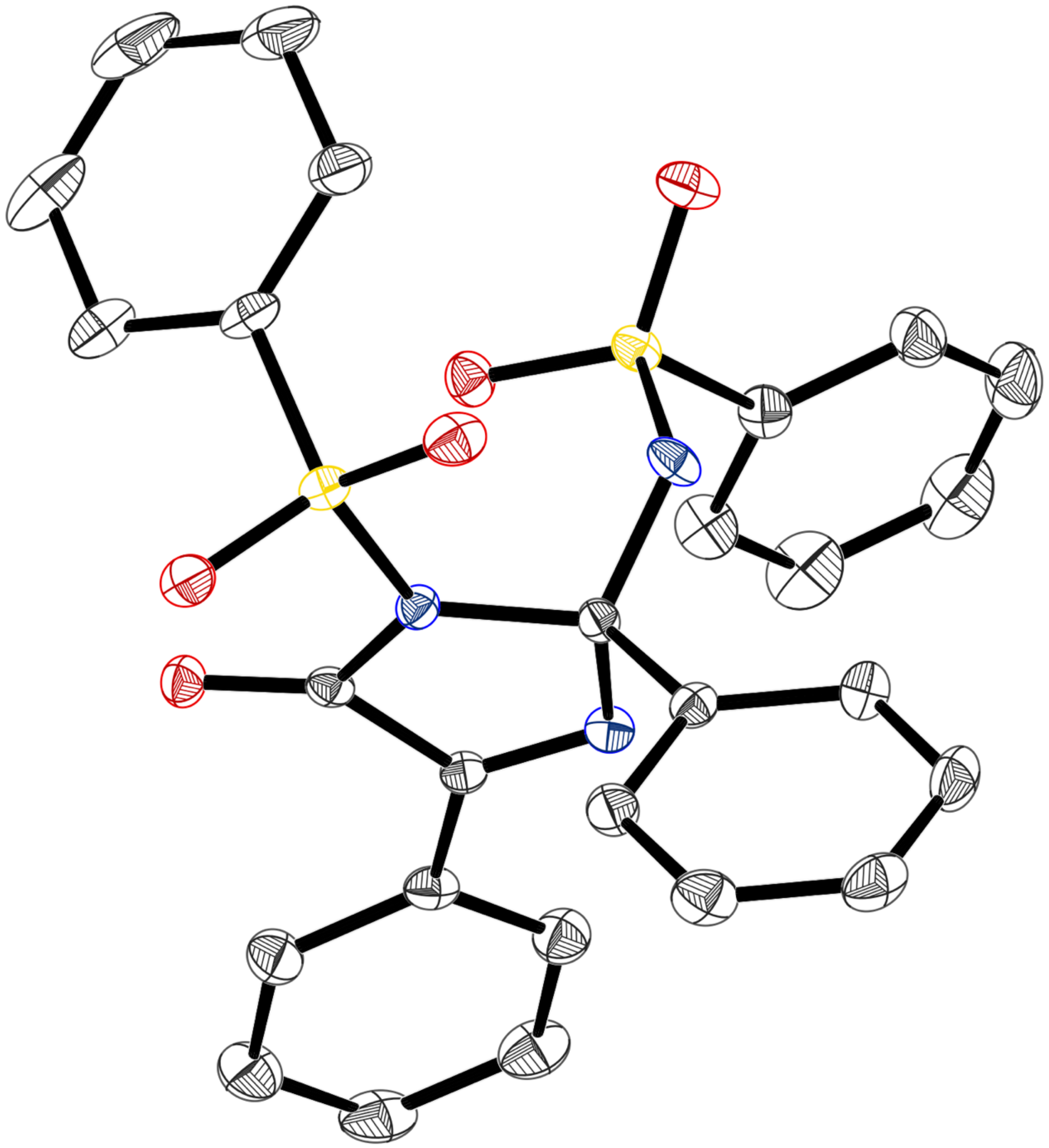

ORTEP drawing of Stomidazolone (AYSJ929) with 50% thermal probability. All hydrogen atoms and solvent molecules are omitted for clarity.

3 ^{1}H and ^{13}C NMR Spectra

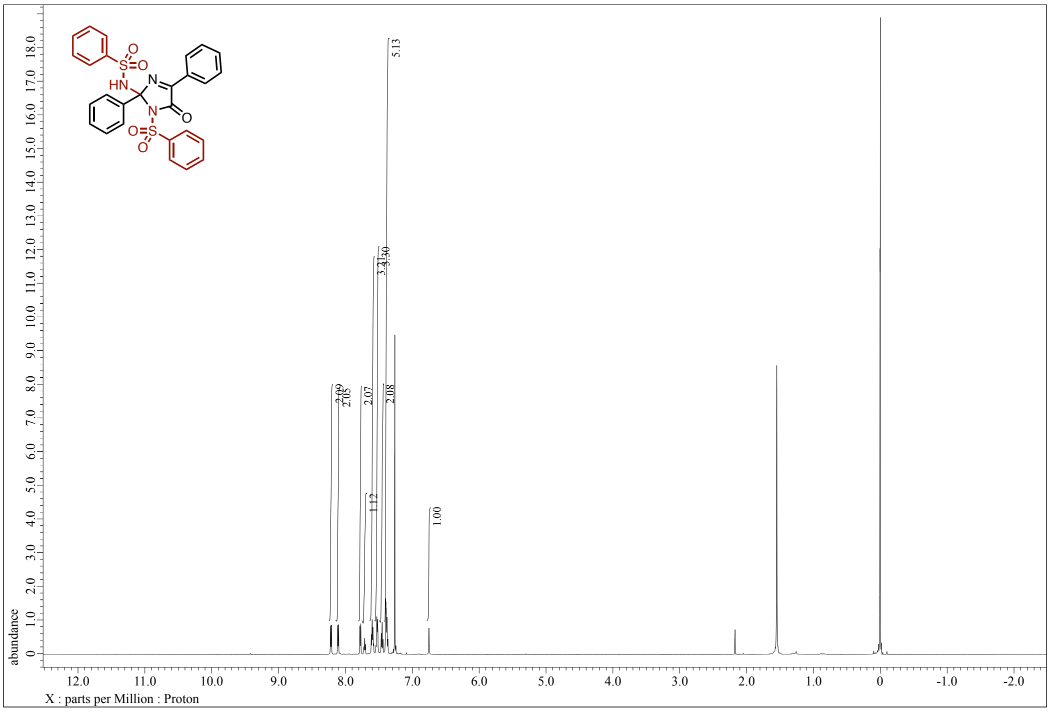

^{1}H NMR (600 MHz, $CDCl_3$) spectrum of Stomidazolone (AYSJ929)

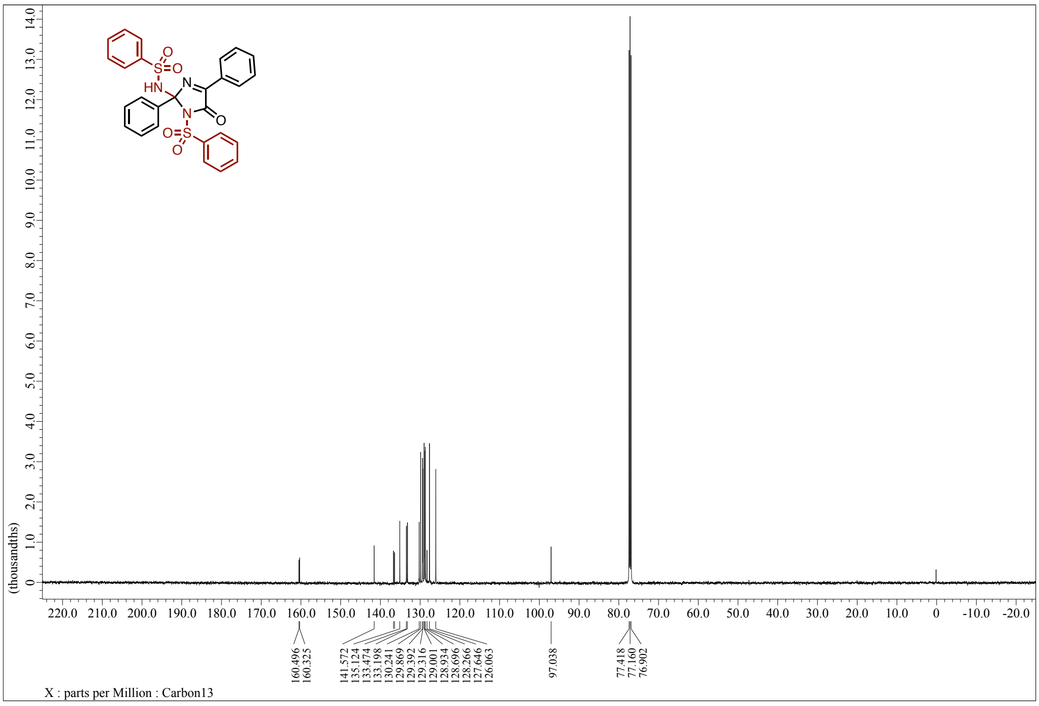

^{13}C NMR (126 MHz, $CDCl_3$) spectrum of Stomidazolone (AYSJ929)

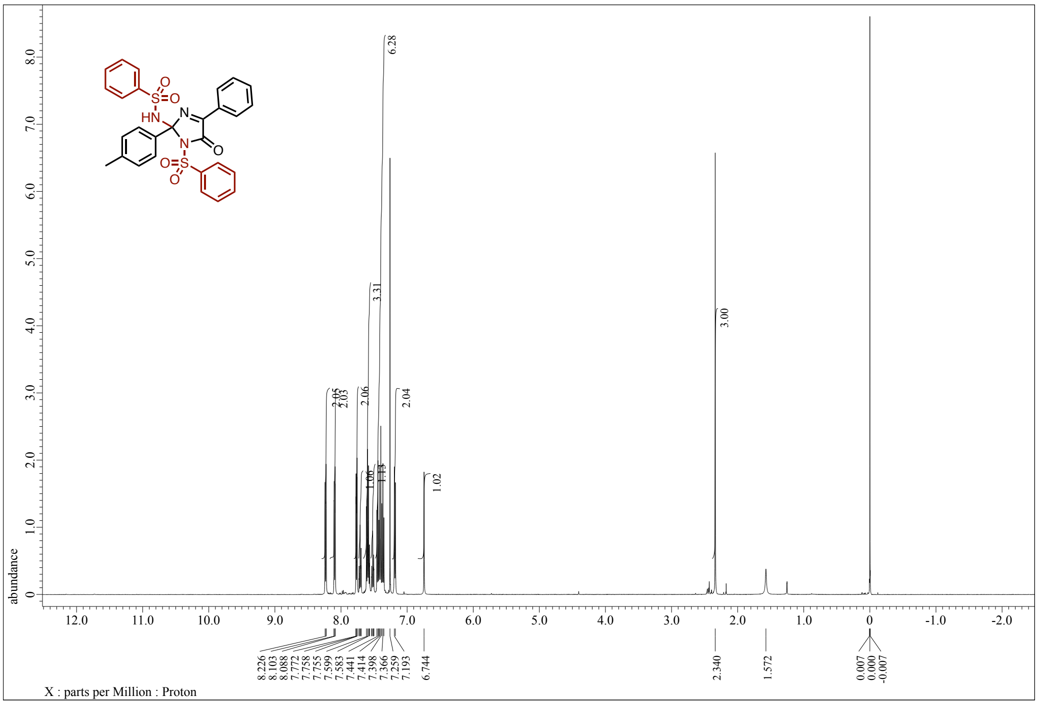

^{1}H NMR (500 MHz, $CDCl_3$) spectrum of AYSJ1059

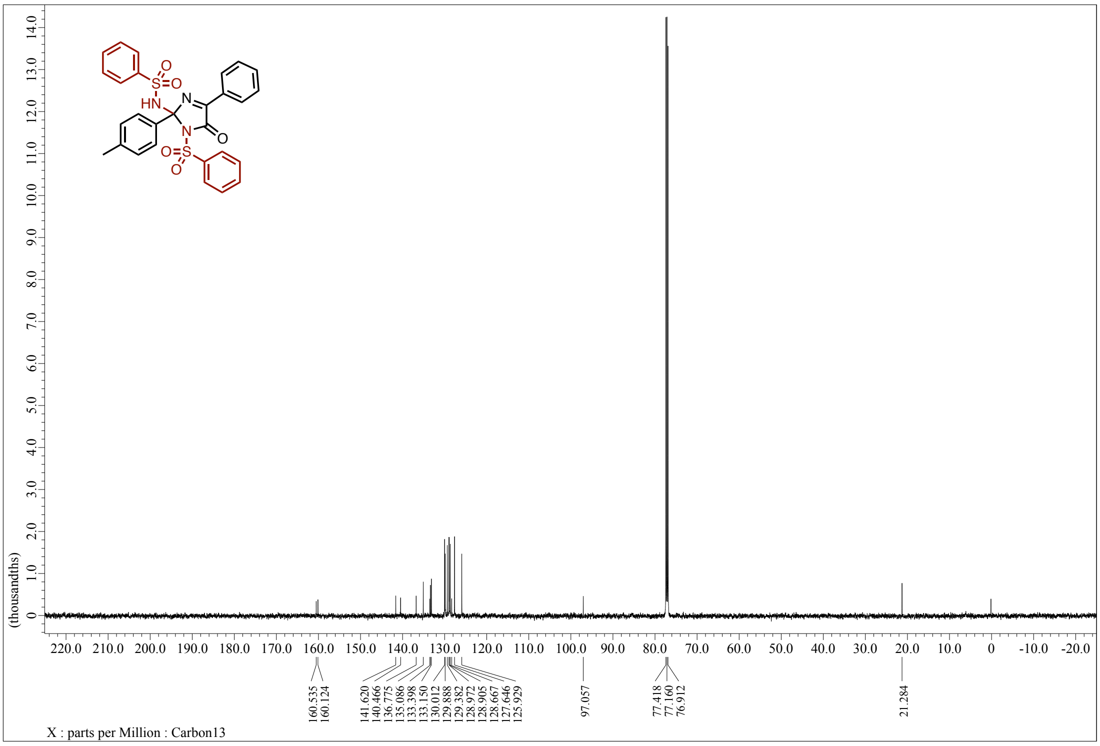

^{13}C NMR (126 MHz, $CDCl_3$) spectrum of AYSJ1059

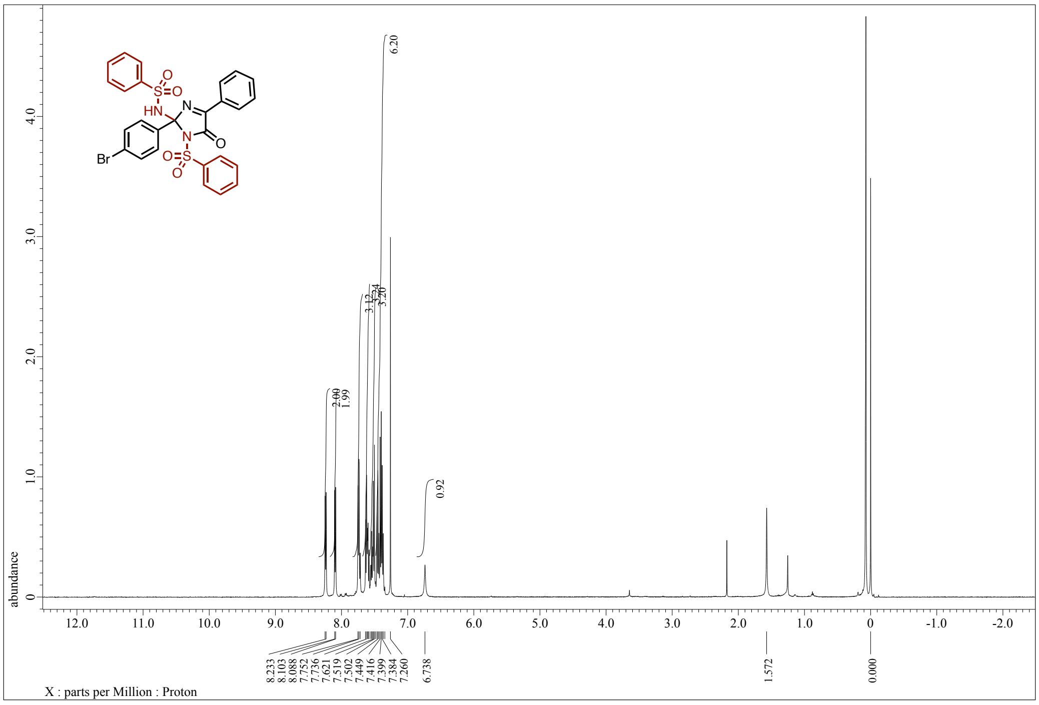

^{1}H NMR (500 MHz, $CDCl_3$) spectrum of AYSJ1061

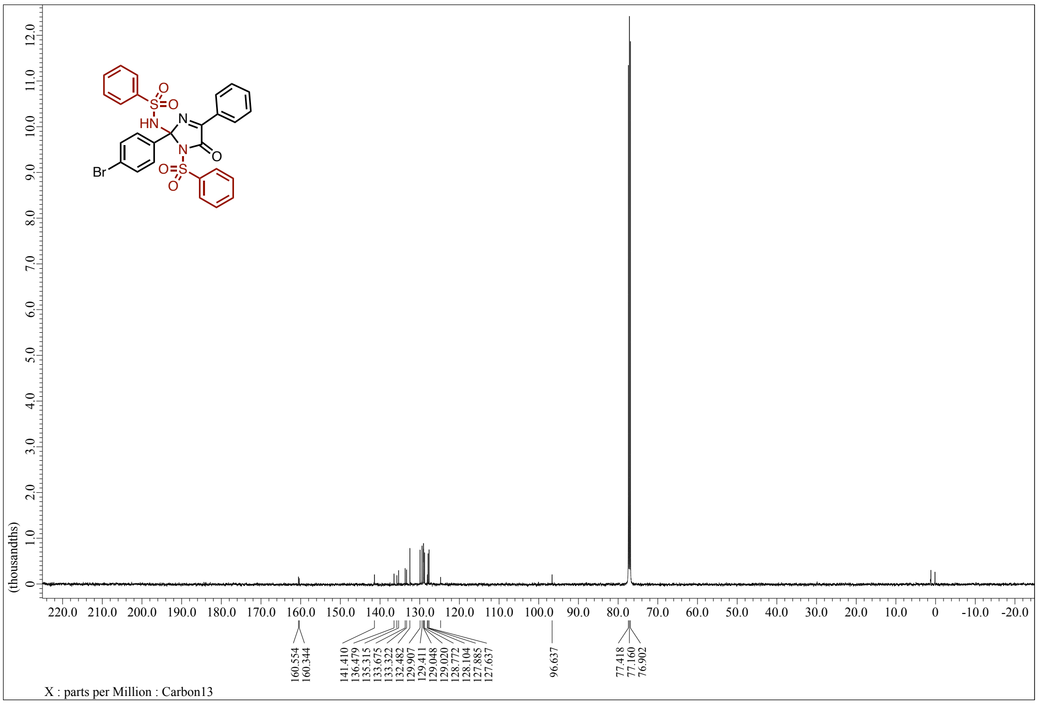

^{13}C NMR (126 MHz, $CDCl_3$) spectrum of AYSJ1061

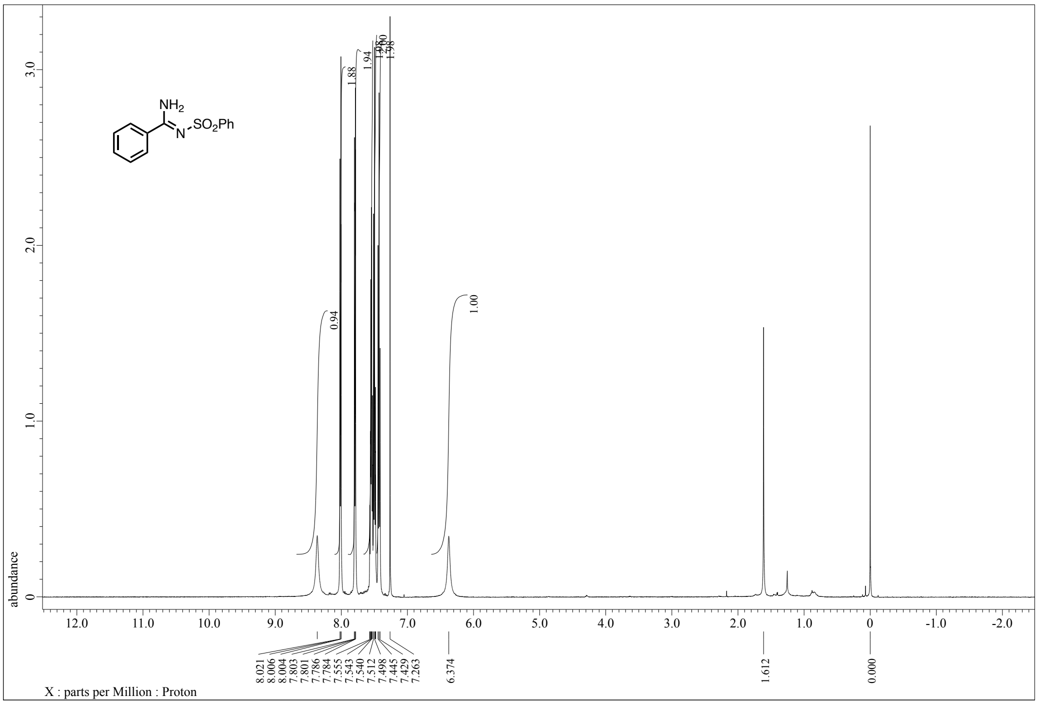

^{1}H NMR (500 MHz, $CDCl_3$) spectrum of lzx103

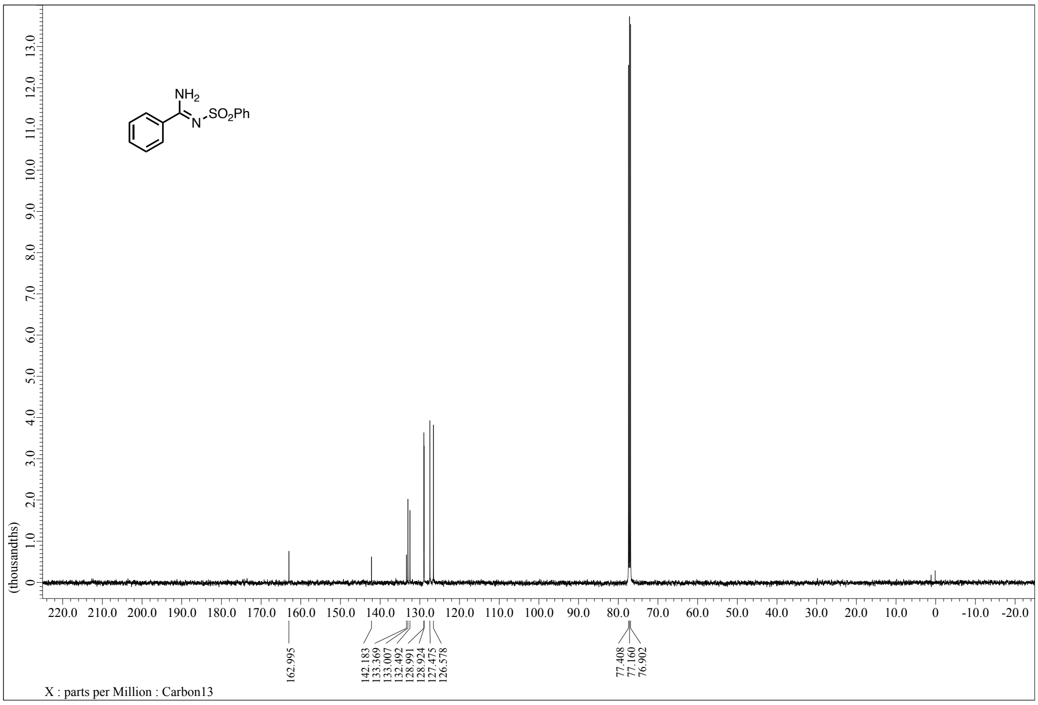

^{13}C NMR (126 MHz, $CDCl_3$) spectrum of lzx103

4 HRMS Analysis

データ:khs-460-hrms
試料名:
説明:
イオン化モード:ESI+
処理履歴:m/z軸決定[ピーク検出[頂上,面積];ベース補正[2.0%];平滑化[5]];ベース補正[5.0%];平均(MS[1] 0.16..0.26)

測定日時:2024/05/08 18:43:14
測定者:AccuTOF
質量校正データ:(内部質量校正データ)
作成日時:2024/05/08 18:50:47
作成者:AccuTOF

電荷数:1 許容誤差:50.00(ppm), 5.00 .. 15.00(mmu) 不飽和数:-1.5 .. 150.0 (端数:両方)
元素:^{12}C:15 .. 19, ^{1}H:11 .. 11, ^{2}H:0 .. 0, ^{10}B:0 .. 0, ^{11}B:0 .. 0, ^{79}Br:1 .. 1, ^{35}Cl:0 .. 0, ^{19}F:0 .. 0, ^{127}I:0 .. 0, ^{14}N:1 .. 1, ^{23}Na:0 .. 0, ^{16}O:1 .. 1, ^{31}P:0 .. 0, ^{32}S:0 .. 0, ^{28}Si:0 .. 0

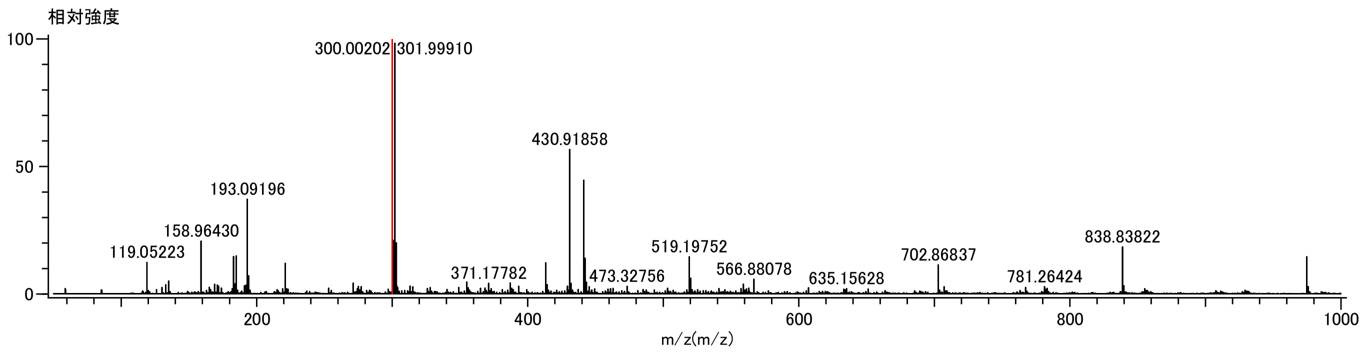

質量	強度	計算質量	質量差 mmu	質量差 ppm	推定組成式	不飽和数
300.00202	76008.06	300.00240	−0.38	−1.26	$^{12}C_{15}{}^{1}H_{11}{}^{79}Br_{1}{}^{14}N_{1}{}^{16}O_{1}$	10.5

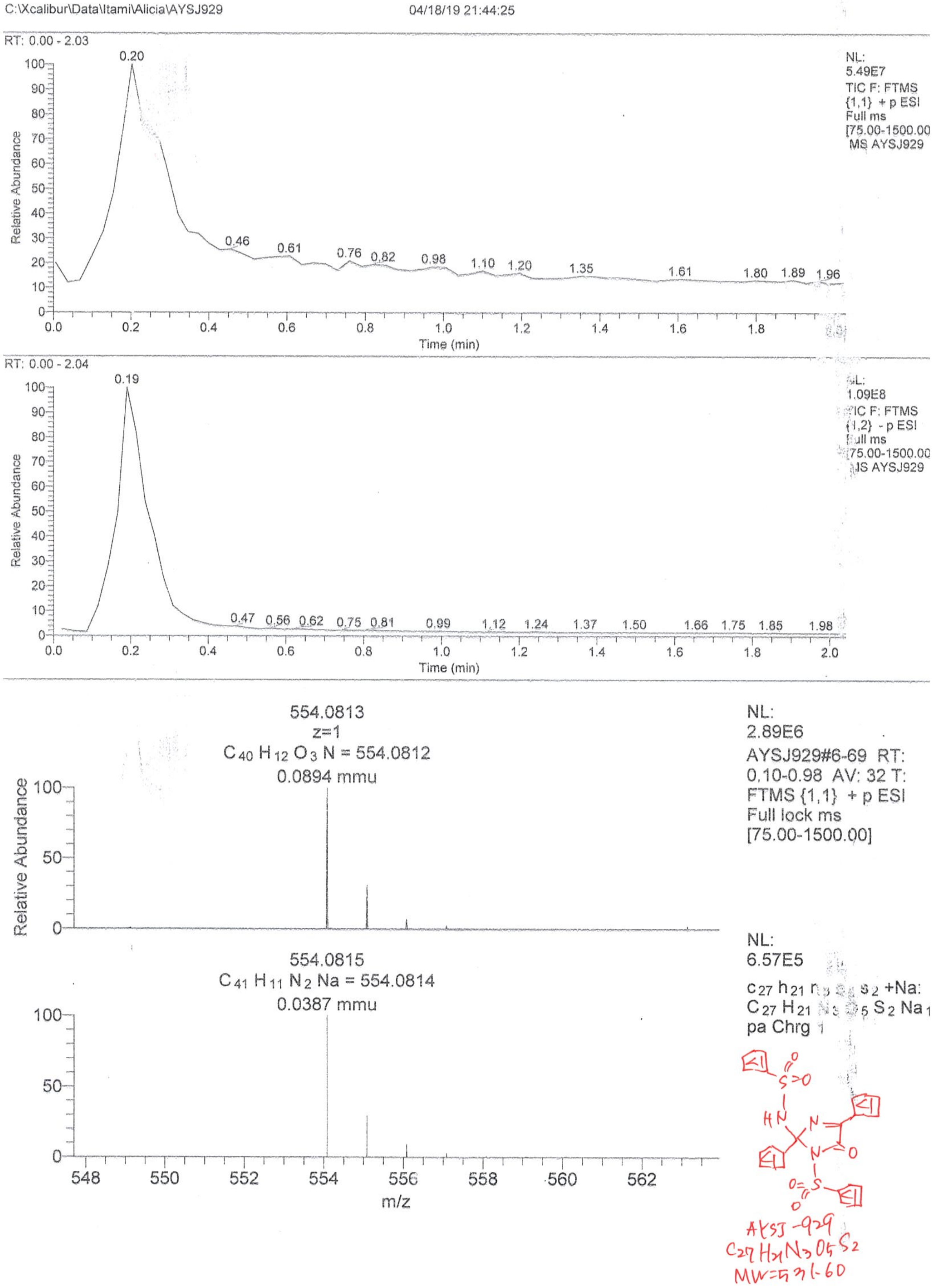
C:\Xcalibur\Data\Itami\Alicia\AYSJ929
04/18/19 21:44:25
RT: 0.00 - 2.03
NL: 5.49E7
TIC F: FTMS {1,1} + p ESI Full ms [75.00-1500.00] MS AYSJ929
Relative Abundance
Time (min)
RT: 0.00 - 2.04
NL: 1.09E8
TIC F: FTMS {1,2} - p ESI Full ms [75.00-1500.00] MS AYSJ929
554.0813
z=1
C 40 H 12 O 3 N = 554.0812
0.0894 mmu
NL: 2.89E6
AYSJ929#6-69 RT: 0.10-0.98 AV: 32 T: FTMS {1,1} + p ESI Full lock ms [75.00-1500.00]
554.0815
C 41 H 11 N 2 Na = 554.0814
0.0387 mmu
NL: 6.57E5
m/z
pa Chrg 1
AYSJ-929
C27H21N3O5S2
MW=531.60

データ:khs-475-hrms
試料名:
説明:
イオン化モード:ESI+
処理履歴:m/z軸決定[ピーク検出[頂上,面積];ベース補正[2.0%];平滑化[5]];ベース補正[5.0%];平均(MS[1] 0.11..0.35)

測定日時:2024/04/19 17:47:49
測定者:AccuTOF
質量校正データ:(内部質量校正データ)
作成日時:2024/04/20 9:37:15
作成者:AccuTOF

電荷数:1
許容誤差:50.00(ppm), 5.00 .. 15.00(mmu)
不飽和数:-1.5 .. 150.0 (端数:両方)
元素:^{12}C:13 .. 13, ^{1}H:12 .. 12, ^{2}H:0 .. 0, ^{11}B:0 .. 0, ^{10}B:0 .. 0, ^{79}Br:0 .. 0, ^{35}Cl:0 .. 0, ^{19}F:0 .. 0, ^{127}I:0 .. 0, ^{14}N:2 .. 2, ^{23}Na:1 .. 1, ^{16}O:2 .. 2, ^{31}P:0 .. 0, ^{32}S:1 .. 1, ^{28}Si:0 .. 0

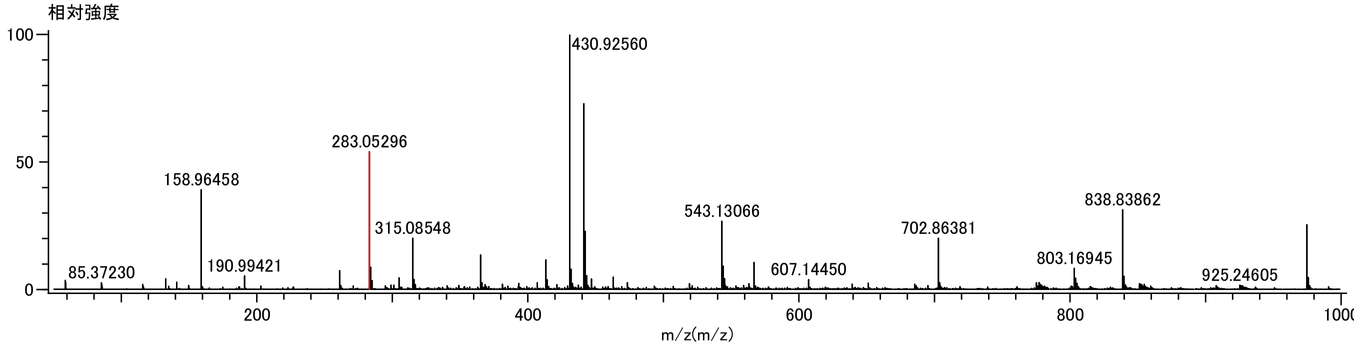

質量	強度	計算質量	質量差 mmu	質量差 ppm	推定組成式	不飽和数
283.05296	88456.72	283.05172	1.25	4.40	$^{12}C_{13}{}^{1}H_{12}{}^{14}N_2{}^{23}Na_1{}^{16}O_2{}^{32}S_1$	9.5

データ:khs-459-hrms
試料名:
説明:
イオン化モード:ESI+
処理履歴:m/z軸決定[ピーク検出[頂上,面積];ベース補正[2.0%];平滑化[5]];ベース補正[5.0%];平均(MS[1] 0.12..0.50)

測定日時:2024/03/19 13:21:09
測定者:AccuTOF
質量校正データ:(内部質量校正データ)
作成日時:2024/03/19 13:51:21
作成者:AccuTOF

電荷数:1
許容誤差:50.00(ppm), 5.00 .. 15.00(mmu)
不飽和数:-1.5 .. 150.0 (端数:両方)
元素:^{12}C:28 .. 28, ^{1}H:23 .. 23, ^{2}H:0 .. 0, ^{10}B:0 .. 0, ^{11}B:0 .. 0, ^{79}Br:0 .. 0, ^{35}Cl:0 .. 0, ^{19}F:0 .. 0, ^{127}I:0 .. 0, ^{14}N:3 .. 3, ^{23}Na:1 .. 1, ^{16}O:5 .. 5, ^{31}P:0 .. 0, ^{32}S:2 .. 2, ^{28}Si:0 .. 0

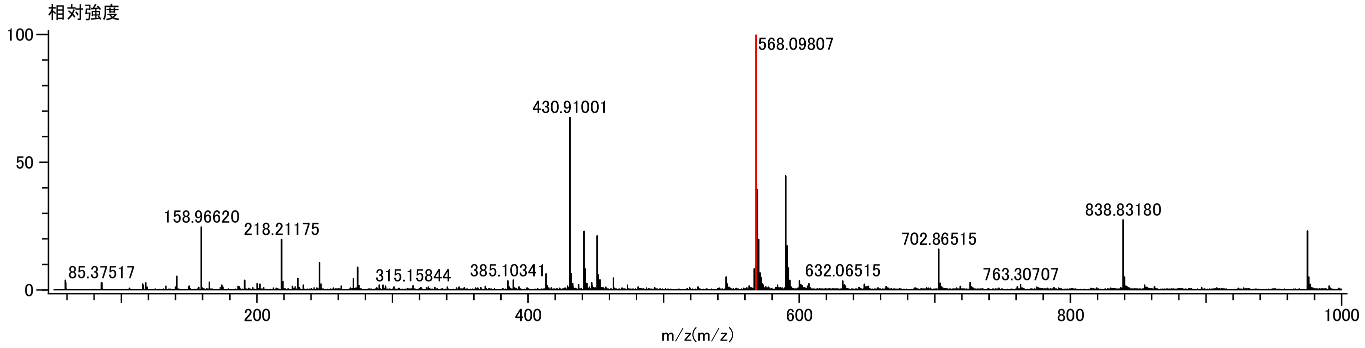

質量	強度	計算質量	質量差 mmu	質量差 ppm	推定組成式	不飽和数
568.09807	81062.92	568.09768	0.39	0.69	$^{12}C_{28}{}^{1}H_{23}{}^{14}N_{3}{}^{23}Na_{1}{}^{16}O_{5}{}^{32}S_{2}$	20.5

データ:khs-466-hrms
試料名:
説明:
イオン化モード:ESI+
処理履歴:m/z軸決定[ピーク検出[頂上,面積];ベース補正[2.0%];平滑化[5]];ベース補正[5.0%];平均(MS[1] 0.14..0.25)

測定日時:2024/03/19 13:32:01
測定者:AccuTOF
質量校正データ:(内部質量校正データ)
作成日時:2024/03/19 14:17:04
作成者:AccuTOF

電荷数:1
許容誤差:50.00(ppm), 5.00 .. 15.00(mmu)
不飽和数:-1.5 .. 150.0 (端数:両方)
元素:^{12}C:27 .. 27, ^{1}H:20 .. 20, ^{2}H:0 .. 0, ^{10}B:0 .. 0, ^{11}B:0 .. 0, ^{79}Br:1 .. 1, ^{35}Cl:0 .. 0, ^{19}F:0 .. 0, ^{127}I:0 .. 0, ^{14}N:3 .. 3, ^{23}Na:1 .. 1, ^{16}O:5 .. 5, ^{31}P:0 .. 0, ^{32}S:2 .. 2, ^{28}Si:0 .. 0

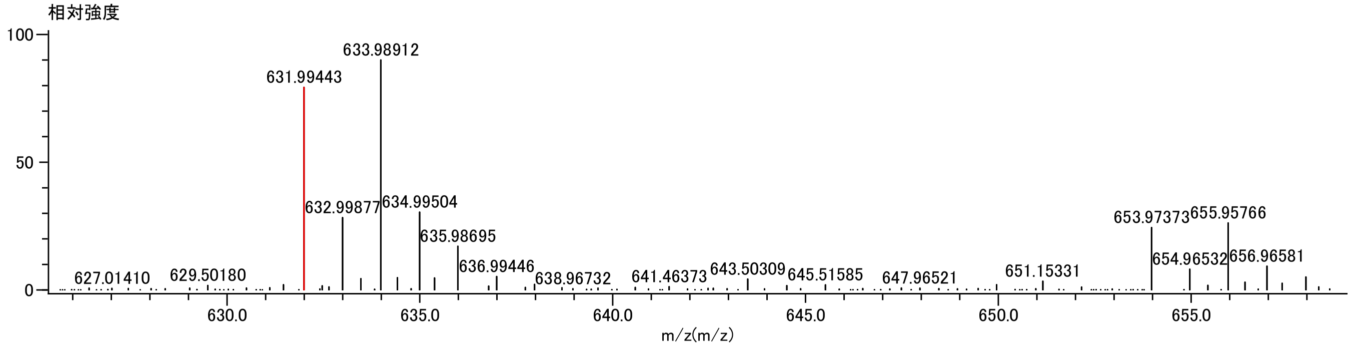

質量	強度	計算質量	質量差 mmu	質量差 ppm	推定組成式	不飽和数
631.99443	55006.33	631.99254	1.88	2.98	$^{12}C_{27}{}^{1}H_{20}{}^{79}Br_{1}{}^{14}N_{3}{}^{23}Na_{1}{}^{16}O_{5}{}^{32}S_{2}$	20.5

4 References

[1] (a) Bailey, J. L.; Sudini, R. R. *Tetrahedron Lett.* **2014**, *55*, 3674. (b) Yoneyama, H.; Oka, N.; Usami, Y.; Harusawa, S. *Tetrahedron Lett.* **2020**, *61*, 151983.

[2] Sheldrick, G. M. *Acta Crystallogr.* **2015**, *A71*, 3.

[3] Sheldrick, G. M. *Acta Crystallogr.* **2015**, *C71*, 3.

[4] Dolomanov, O. V.; Bourhis, L. J.; Gildea, R. J.; Howard, J. A. K.; Puschmann, H. *J. Appl. Crystallogr.* **2009**, *42*, 339.
