# Supplementary material for: Chemical inhibition of stomatal differentiation by perturbation of the master-regulatory bHLH heterodimer via an ACT-Like domain
Source: Nat Commun. 2024 Oct 23;15:8996. doi: 10.1038/s41467-024-53214-4 (PMC11500415; doi:10.1038/s41467-024-53214-4)
Supplement: Supplementary file 5 — Supplementary Data 2 [file 41467_2024_53214_MOESM5_ESM.pdf]

MUTE_ACT_Stomidazolone (a form)

```
ATOM      1  N   VAL A 106     -14.923  -7.079 -10.220  1.00 15.00      A    N
ATOM      2  CA  VAL A 106     -14.538  -5.671 -10.198  1.00 15.00      A    C
ATOM      3  C   VAL A 106     -13.276  -5.459  -9.362  1.00 15.00      A    C
ATOM      4  O   VAL A 106     -13.286  -5.653  -8.145  1.00 15.00      A    O
ATOM      5  CB  VAL A 106     -15.672  -4.779  -9.648  1.00 15.00      A    C
ATOM      6  CG1 VAL A 106     -15.324  -3.305  -9.809  1.00 15.00      A    C
ATOM      7  CG2 VAL A 106     -16.989  -5.095 -10.342  1.00 15.00      A    C
ATOM      8  HN  VAL A 106     -15.863  -7.320 -10.084  1.00 15.00      A    H
ATOM      9  N   GLY A 107     -12.193  -5.061 -10.022  1.00 15.00      A    N
ATOM     10  CA  GLY A 107     -10.941  -4.836  -9.325  1.00 15.00      A    C
ATOM     11  C   GLY A 107      -9.954  -4.021 -10.138  1.00 15.00      A    C
ATOM     12  O   GLY A 107     -10.042  -3.971 -11.364  1.00 15.00      A    O
ATOM     13  HN  GLY A 107     -12.246  -4.916 -10.990  1.00 15.00      A    H
ATOM     14  N   ALA A 108      -9.010  -3.385  -9.452  1.00 15.00      A    N
ATOM     15  CA  ALA A 108      -8.001  -2.568 -10.112  1.00 15.00      A    C
ATOM     16  C   ALA A 108      -6.599  -3.017  -9.727  1.00 15.00      A    C
ATOM     17  O   ALA A 108      -6.390  -3.568  -8.647  1.00 15.00      A    O
ATOM     18  CB  ALA A 108      -8.192  -1.105  -9.754  1.00 15.00      A    C
ATOM     19  HN  ALA A 108      -8.990  -3.470  -8.475  1.00 15.00      A    H
ATOM     20  N   CYS A 109      -5.639  -2.769 -10.613  1.00 15.00      A    N
ATOM     21  CA  CYS A 109      -4.255  -3.147 -10.363  1.00 15.00      A    C
ATOM     22  C   CYS A 109      -3.274  -2.081 -10.845  1.00 15.00      A    C
ATOM     23  O   CYS A 109      -3.421  -1.528 -11.934  1.00 15.00      A    O
ATOM     24  CB  CYS A 109      -3.931  -4.500 -11.008  1.00 15.00      A    C
ATOM     25  SG  CYS A 109      -4.271  -4.585 -12.783  1.00 15.00      A    S
ATOM     26  HN  CYS A 109      -5.868  -2.320 -11.454  1.00 15.00      A    H
ATOM     27  HG  CYS A 109      -5.577  -4.742 -12.941  1.00 15.00      A    H
ATOM     28  N   CYS A 110      -2.279  -1.794 -10.016  1.00 15.00      A    N
ATOM     29  CA  CYS A 110      -1.256  -0.811 -10.346  1.00 15.00      A    C
ATOM     30  C   CYS A 110       0.127  -1.432 -10.210  1.00 15.00      A    C
ATOM     31  O   CYS A 110       0.605  -1.672  -9.101  1.00 15.00      A    O
ATOM     32  CB  CYS A 110      -1.376   0.417  -9.441  1.00 15.00      A    C
ATOM     33  SG  CYS A 110      -0.156   1.710  -9.776  1.00 15.00      A    S
ATOM     34  HN  CYS A 110      -2.232  -2.256  -9.152  1.00 15.00      A    H
ATOM     35  HG  CYS A 110       1.013   1.124  -9.998  1.00 15.00      A    H
ATOM     36  N   ASN A 111       0.765  -1.685 -11.346  1.00 15.00      A    N
ATOM     37  CA  ASN A 111       2.088  -2.297 -11.364  1.00 15.00      A    C
ATOM     38  C   ASN A 111       3.197  -1.265 -11.548  1.00 15.00      A    C
ATOM     39  O   ASN A 111       3.348  -0.684 -12.623  1.00 15.00      A    O
ATOM     40  CB  ASN A 111       2.170  -3.353 -12.470  1.00 15.00      A    C
ATOM     41  CG  ASN A 111       1.239  -4.529 -12.237  1.00 15.00      A    C
ATOM     42  ND2 ASN A 111       0.748  -5.118 -13.323  1.00 15.00      A    N
ATOM     43  OD1 ASN A 111       0.968  -4.903 -11.099  1.00 15.00      A    O
ATOM     44  HN  ASN A 111       0.336  -1.451 -12.194  1.00 15.00      A    H
ATOM     45 HD21 ASN A 111       1.009  -4.766 -14.200  1.00 15.00      A    H
```

```
ATOM     46 HD22 ASN A 111       0.143  -5.881 -13.203  1.00 15.00      A    H
ATOM     47  N   SER A 112       3.969  -1.046 -10.490  1.00 15.00      A    N
ATOM     48  CA  SER A 112       5.080  -0.101 -10.528  1.00 15.00      A    C
ATOM     49  C   SER A 112       6.373  -0.805 -10.115  1.00 15.00      A    C
ATOM     50  O   SER A 112       6.326  -1.861  -9.482  1.00 15.00      A    O
ATOM     51  CB  SER A 112       4.801   1.086  -9.599  1.00 15.00      A    C
ATOM     52  OG  SER A 112       4.973   0.723  -8.243  1.00 15.00      A    O
ATOM     53  HN  SER A 112       3.785  -1.530  -9.657  1.00 15.00      A    H
ATOM     54  HG  SER A 112       5.513  -0.071  -8.190  1.00 15.00      A    H
ATOM     55  N   PRO A 113       7.544  -0.235 -10.470  1.00 15.00      A    N
ATOM     56  CA  PRO A 113       8.851  -0.820 -10.121  1.00 15.00      A    C
ATOM     57  C   PRO A 113       9.112  -0.829  -8.613  1.00 15.00      A    C
ATOM     58  O   PRO A 113      10.064  -1.452  -8.144  1.00 15.00      A    O
ATOM     59  CB  PRO A 113       9.861   0.100 -10.821  1.00 15.00      A    C
ATOM     60  CG  PRO A 113       9.066   0.851 -11.836  1.00 15.00      A    C
ATOM     61  CD  PRO A 113       7.694   1.004 -11.251  1.00 15.00      A    C
ATOM     62  N   HIS A 114       8.261  -0.136  -7.858  1.00 15.00      A    N
ATOM     63  CA  HIS A 114       8.410  -0.066  -6.410  1.00 15.00      A    C
ATOM     64  C   HIS A 114       7.488  -1.046  -5.689  1.00 15.00      A    C
ATOM     65  O   HIS A 114       7.911  -1.717  -4.747  1.00 15.00      A    O
ATOM     66  CB  HIS A 114       8.165   1.359  -5.905  1.00 15.00      A    C
ATOM     67  CG  HIS A 114       9.036   2.387  -6.562  1.00 15.00      A    C
ATOM     68  CD2 HIS A 114       8.718   3.312  -7.502  1.00 15.00      A    C
ATOM     69  ND1 HIS A 114      10.374   2.486  -6.264  1.00 15.00      A    N
ATOM     70  CE1 HIS A 114      10.843   3.459  -7.024  1.00 15.00      A    C
ATOM     71  NE2 HIS A 114       9.877   3.988  -7.788  1.00 15.00      A    N
ATOM     72  HN  HIS A 114       7.522   0.341  -8.287  1.00 15.00      A    H
ATOM     73  HE2 HIS A 114       9.977   4.723  -8.430  1.00 15.00      A    H
ATOM     74  N   ALA A 115       6.230  -1.128  -6.129  1.00 15.00      A    N
ATOM     75  CA  ALA A 115       5.263  -2.026  -5.497  1.00 15.00      A    C
ATOM     76  C   ALA A 115       4.101  -2.394  -6.416  1.00 15.00      A    C
ATOM     77  O   ALA A 115       3.745  -1.650  -7.331  1.00 15.00      A    O
ATOM     78  CB  ALA A 115       4.729  -1.410  -4.212  1.00 15.00      A    C
ATOM     79  HN  ALA A 115       5.949  -0.580  -6.891  1.00 15.00      A    H
ATOM     80  N   ASN A 116       3.510  -3.555  -6.148  1.00 15.00      A    N
ATOM     81  CA  ASN A 116       2.367  -4.052  -6.911  1.00 15.00      A    C
ATOM     82  C   ASN A 116       1.108  -4.001  -6.041  1.00 15.00      A    C
ATOM     83  O   ASN A 116       0.984  -4.747  -5.068  1.00 15.00      A    O
ATOM     84  CB  ASN A 116       2.642  -5.492  -7.371  1.00 15.00      A    C
ATOM     85  CG  ASN A 116       1.494  -6.139  -8.134  1.00 15.00      A    C
ATOM     86  ND2 ASN A 116       1.663  -7.415  -8.476  1.00 15.00      A    N
ATOM     87  OD1 ASN A 116       0.478  -5.507  -8.423  1.00 15.00      A    O
ATOM     88  HN  ASN A 116       3.857  -4.101  -5.410  1.00 15.00      A    H
ATOM     89 HD21 ASN A 116       2.502  -7.854  -8.224  1.00 15.00      A    H
ATOM     90 HD22 ASN A 116       0.940  -7.866  -8.964  1.00 15.00      A    H
ATOM     91  N   VAL A 117       0.180  -3.116  -6.393  1.00 15.00      A    N
ATOM     92  CA  VAL A 117      -1.057  -2.957  -5.632  1.00 15.00      A    C
```

```
ATOM     93  C   VAL A 117      -2.254  -3.556  -6.374  1.00 15.00      A    C
ATOM     94  O   VAL A 117      -2.472  -3.273  -7.548  1.00 15.00      A    O
ATOM     95  CB  VAL A 117      -1.339  -1.469  -5.322  1.00 15.00      A    C
ATOM     96  CG1 VAL A 117      -2.398  -1.333  -4.237  1.00 15.00      A    C
ATOM     97  CG2 VAL A 117      -0.062  -0.750  -4.912  1.00 15.00      A    C
ATOM     98  HN  VAL A 117       0.330  -2.558  -7.184  1.00 15.00      A    H
ATOM     99  N   GLU A 118      -3.029  -4.381  -5.671  1.00 15.00      A    N
ATOM    100  CA  GLU A 118      -4.206  -5.021  -6.253  1.00 15.00      A    C
ATOM    101  C   GLU A 118      -5.409  -4.937  -5.310  1.00 15.00      A    C
ATOM    102  O   GLU A 118      -5.379  -5.472  -4.203  1.00 15.00      A    O
ATOM    103  CB  GLU A 118      -3.903  -6.487  -6.577  1.00 15.00      A    C
ATOM    104  CG  GLU A 118      -5.053  -7.235  -7.233  1.00 15.00      A    C
ATOM    105  CD  GLU A 118      -4.813  -8.732  -7.312  1.00 15.00      A    C
ATOM    106  OE1 GLU A 118      -5.773  -9.469  -7.624  1.00 15.00      A    O
ATOM    107  OE2 GLU A 118      -3.670  -9.167  -7.062  1.00 15.00      A    O1-
ATOM    108  HN  GLU A 118      -2.799  -4.567  -4.736  1.00 15.00      A    H
ATOM    109  N   ALA A 119      -6.470  -4.271  -5.758  1.00 15.00      A    N
ATOM    110  CA  ALA A 119      -7.678  -4.123  -4.950  1.00 15.00      A    C
ATOM    111  C   ALA A 119      -8.887  -4.760  -5.627  1.00 15.00      A    C
ATOM    112  O   ALA A 119      -9.149  -4.517  -6.802  1.00 15.00      A    O
ATOM    113  CB  ALA A 119      -7.947  -2.654  -4.661  1.00 15.00      A    C
ATOM    114  HN  ALA A 119      -6.440  -3.873  -6.654  1.00 15.00      A    H
ATOM    115  N   LYS A 120      -9.622  -5.572  -4.874  1.00 15.00      A    N
ATOM    116  CA  LYS A 120     -10.811  -6.243  -5.395  1.00 15.00      A    C
ATOM    117  C   LYS A 120     -11.982  -6.117  -4.425  1.00 15.00      A    C
ATOM    118  O   LYS A 120     -11.785  -5.984  -3.221  1.00 15.00      A    O
ATOM    119  CB  LYS A 120     -10.520  -7.720  -5.678  1.00 15.00      A    C
ATOM    120  CG  LYS A 120      -9.861  -7.978  -7.024  1.00 15.00      A    C
ATOM    121  CD  LYS A 120      -9.473  -9.440  -7.185  1.00 15.00      A    C
ATOM    122  CE  LYS A 120      -8.912  -9.709  -8.572  1.00 15.00      A    C
ATOM    123  NZ  LYS A 120      -8.320 -11.073  -8.682  1.00 15.00      A    N1+
ATOM    124  HN  LYS A 120      -9.359  -5.725  -3.942  1.00 15.00      A    H
ATOM    125  HZ1 LYS A 120      -8.061 -11.271  -9.671  1.00 15.00      A    H
ATOM    126  HZ2 LYS A 120      -9.004 -11.789  -8.364  1.00 15.00      A    H
ATOM    127  HZ3 LYS A 120      -7.465 -11.140  -8.093  1.00 15.00      A    H
ATOM    128  N   ILE A 121     -13.200  -6.157  -4.957  1.00 15.00      A    N
ATOM    129  CA  ILE A 121     -14.395  -6.048  -4.129  1.00 15.00      A    C
ATOM    130  C   ILE A 121     -14.885  -7.422  -3.680  1.00 15.00      A    C
ATOM    131  O   ILE A 121     -15.226  -8.275  -4.502  1.00 15.00      A    O
ATOM    132  CB  ILE A 121     -15.538  -5.301  -4.852  1.00 15.00      A    C
ATOM    133  CG1 ILE A 121     -15.120  -3.861  -5.159  1.00 15.00      A    C
ATOM    134  CG2 ILE A 121     -16.813  -5.317  -4.014  1.00 15.00      A    C
ATOM    135  CD1 ILE A 121     -16.165  -3.060  -5.904  1.00 15.00      A    C
ATOM    136  HN  ILE A 121     -13.296  -6.262  -5.926  1.00 15.00      A    H
ATOM    137  N   SER A 122     -14.911  -7.628  -2.369  1.00 15.00      A    N
ATOM    138  CA  SER A 122     -15.367  -8.886  -1.797  1.00 15.00      A    C
ATOM    139  C   SER A 122     -16.526  -8.639  -0.835  1.00 15.00      A    C
```

```
ATOM    140  O    SER A 122     -16.325  -8.455   0.367  1.00 15.00      A    O
ATOM    141  CB   SER A 122     -14.217  -9.603  -1.084  1.00 15.00      A    C
ATOM    142  OG   SER A 122     -14.620 -10.875  -0.605  1.00 15.00      A    O
ATOM    143  HN   SER A 122     -14.616  -6.912  -1.770  1.00 15.00      A    H
ATOM    144  HG   SER A 122     -13.920 -11.514  -0.760  1.00 15.00      A    H
ATOM    145  N    GLY A 123     -17.739  -8.624  -1.382  1.00 15.00      A    N
ATOM    146  CA   GLY A 123     -18.923  -8.387  -0.574  1.00 15.00      A    C
ATOM    147  C    GLY A 123     -19.120  -6.912  -0.277  1.00 15.00      A    C
ATOM    148  O    GLY A 123     -19.396  -6.120  -1.179  1.00 15.00      A    O
ATOM    149  HN   GLY A 123     -17.831  -8.776  -2.345  1.00 15.00      A    H
ATOM    150  N    SER A 124     -18.971  -6.543   0.989  1.00 15.00      A    N
ATOM    151  CA   SER A 124     -19.114  -5.152   1.409  1.00 15.00      A    C
ATOM    152  C    SER A 124     -17.762  -4.590   1.833  1.00 15.00      A    C
ATOM    153  O    SER A 124     -17.671  -3.483   2.366  1.00 15.00      A    O
ATOM    154  CB   SER A 124     -20.112  -5.043   2.565  1.00 15.00      A    C
ATOM    155  OG   SER A 124     -21.406  -5.459   2.162  1.00 15.00      A    O
ATOM    156  HN   SER A 124     -18.753  -7.223   1.660  1.00 15.00      A    H
ATOM    157  HG   SER A 124     -22.053  -4.809   2.445  1.00 15.00      A    H
ATOM    158  N    ASN A 125     -16.713  -5.368   1.581  1.00 15.00      A    N
ATOM    159  CA   ASN A 125     -15.353  -4.982   1.937  1.00 15.00      A    C
ATOM    160  C    ASN A 125     -14.458  -4.959   0.699  1.00 15.00      A    C
ATOM    161  O    ASN A 125     -14.911  -5.247  -0.408  1.00 15.00      A    O
ATOM    162  CB   ASN A 125     -14.797  -5.971   2.966  1.00 15.00      A    C
ATOM    163  CG   ASN A 125     -15.683  -6.105   4.193  1.00 15.00      A    C
ATOM    164  ND2  ASN A 125     -15.996  -7.346   4.566  1.00 15.00      A    N
ATOM    165  OD1  ASN A 125     -16.095  -5.110   4.789  1.00 15.00      A    O
ATOM    166  HN   ASN A 125     -16.860  -6.229   1.137  1.00 15.00      A    H
ATOM    167  HD21 ASN A 125     -15.642  -8.092   4.037  1.00 15.00      A    H
ATOM    168  HD22 ASN A 125     -16.569  -7.463   5.354  1.00 15.00      A    H
ATOM    169  N    VAL A 126     -13.187  -4.601   0.889  1.00 15.00      A    N
ATOM    170  CA   VAL A 126     -12.227  -4.546  -0.214  1.00 15.00      A    C
ATOM    171  C    VAL A 126     -10.919  -5.246   0.150  1.00 15.00      A    C
ATOM    172  O    VAL A 126     -10.269  -4.900   1.139  1.00 15.00      A    O
ATOM    173  CB   VAL A 126     -11.927  -3.092  -0.654  1.00 15.00      A    C
ATOM    174  CG1  VAL A 126     -10.796  -3.055  -1.672  1.00 15.00      A    C
ATOM    175  CG2  VAL A 126     -13.171  -2.437  -1.232  1.00 15.00      A    C
ATOM    176  HN   VAL A 126     -12.887  -4.374   1.795  1.00 15.00      A    H
ATOM    177  N    VAL A 127     -10.541  -6.230  -0.661  1.00 15.00      A    N
ATOM    178  CA   VAL A 127      -9.309  -6.981  -0.447  1.00 15.00      A    C
ATOM    179  C    VAL A 127      -8.129  -6.286  -1.125  1.00 15.00      A    C
ATOM    180  O    VAL A 127      -8.023  -6.270  -2.351  1.00 15.00      A    O
ATOM    181  CB   VAL A 127      -9.426  -8.422  -0.986  1.00 15.00      A    C
ATOM    182  CG1  VAL A 127      -8.148  -9.204  -0.727  1.00 15.00      A    C
ATOM    183  CG2  VAL A 127     -10.619  -9.131  -0.367  1.00 15.00      A    C
ATOM    184  HN   VAL A 127     -11.108  -6.456  -1.428  1.00 15.00      A    H
ATOM    185  N    LEU A 128      -7.249  -5.712  -0.315  1.00 15.00      A    N
ATOM    186  CA   LEU A 128      -6.073  -5.018  -0.824  1.00 15.00      A    C
```

ATOM	187	C	LEU	A	128	-4.817	-5.871	-0.655	1.00	15.00	A	C
ATOM	188	O	LEU	A	128	-4.509	-6.331	0.440	1.00	15.00	A	O
ATOM	189	CB	LEU	A	128	-5.901	-3.676	-0.103	1.00	15.00	A	C
ATOM	190	CG	LEU	A	128	-4.811	-2.744	-0.647	1.00	15.00	A	C
ATOM	191	CD1	LEU	A	128	-5.176	-2.249	-2.038	1.00	15.00	A	C
ATOM	192	CD2	LEU	A	128	-4.584	-1.574	0.297	1.00	15.00	A	C
ATOM	193	HN	LEU	A	128	-7.396	-5.757	0.654	1.00	15.00	A	H
ATOM	194	N	ARG	A	129	-4.104	-6.086	-1.753	1.00	15.00	A	N
ATOM	195	CA	ARG	A	129	-2.874	-6.877	-1.734	1.00	15.00	A	C
ATOM	196	C	ARG	A	129	-1.704	-6.032	-2.230	1.00	15.00	A	C
ATOM	197	O	ARG	A	129	-1.705	-5.577	-3.371	1.00	15.00	A	O
ATOM	198	CB	ARG	A	129	-3.022	-8.126	-2.612	1.00	15.00	A	C
ATOM	199	CG	ARG	A	129	-4.147	-9.063	-2.199	1.00	15.00	A	C
ATOM	200	CD	ARG	A	129	-4.278	-10.227	-3.174	1.00	15.00	A	C
ATOM	201	NE	ARG	A	129	-5.349	-11.151	-2.794	1.00	15.00	A	N
ATOM	202	CZ	ARG	A	129	-5.350	-12.453	-3.089	1.00	15.00	A	C
ATOM	203	NH1	ARG	A	129	-4.341	-12.986	-3.766	1.00	15.00	A	N1+
ATOM	204	NH2	ARG	A	129	-6.362	-13.222	-2.702	1.00	15.00	A	N
ATOM	205	HN	ARG	A	129	-4.410	-5.699	-2.601	1.00	15.00	A	H
ATOM	206	HE	ARG	A	129	-6.106	-10.782	-2.294	1.00	15.00	A	H
ATOM	207	HH11	ARG	A	129	-4.345	-14.001	-3.995	1.00	15.00	A	H
ATOM	208	HH12	ARG	A	129	-3.544	-12.389	-4.069	1.00	15.00	A	H
ATOM	209	HH21	ARG	A	129	-6.363	-14.237	-2.933	1.00	15.00	A	H
ATOM	210	HH22	ARG	A	129	-7.155	-12.811	-2.168	1.00	15.00	A	H
ATOM	211	N	VAL	A	130	-0.705	-5.822	-1.373	1.00	15.00	A	N
ATOM	212	CA	VAL	A	130	0.454	-5.006	-1.744	1.00	15.00	A	C
ATOM	213	C	VAL	A	130	1.767	-5.790	-1.685	1.00	15.00	A	C
ATOM	214	O	VAL	A	130	2.227	-6.173	-0.612	1.00	15.00	A	O
ATOM	215	CB	VAL	A	130	0.576	-3.748	-0.854	1.00	15.00	A	C
ATOM	216	CG1	VAL	A	130	1.680	-2.833	-1.365	1.00	15.00	A	C
ATOM	217	CG2	VAL	A	130	-0.746	-3.001	-0.781	1.00	15.00	A	C
ATOM	218	HN	VAL	A	130	-0.745	-6.223	-0.478	1.00	15.00	A	H
ATOM	219	N	VAL	A	131	2.370	-6.015	-2.849	1.00	15.00	A	N
ATOM	220	CA	VAL	A	131	3.638	-6.736	-2.932	1.00	15.00	A	C
ATOM	221	C	VAL	A	131	4.779	-5.764	-3.223	1.00	15.00	A	C
ATOM	222	O	VAL	A	131	4.873	-5.216	-4.320	1.00	15.00	A	O
ATOM	223	CB	VAL	A	131	3.611	-7.821	-4.029	1.00	15.00	A	C
ATOM	224	CG1	VAL	A	131	4.869	-8.677	-3.969	1.00	15.00	A	C
ATOM	225	CG2	VAL	A	131	2.367	-8.688	-3.906	1.00	15.00	A	C
ATOM	226	HN	VAL	A	131	1.951	-5.687	-3.674	1.00	15.00	A	H
ATOM	227	N	SER	A	132	5.647	-5.557	-2.239	1.00	15.00	A	N
ATOM	228	CA	SER	A	132	6.770	-4.636	-2.393	1.00	15.00	A	C
ATOM	229	C	SER	A	132	8.034	-5.184	-1.741	1.00	15.00	A	C
ATOM	230	O	SER	A	132	8.151	-6.383	-1.504	1.00	15.00	A	O
ATOM	231	CB	SER	A	132	6.414	-3.283	-1.776	1.00	15.00	A	C
ATOM	232	OG	SER	A	132	6.026	-3.429	-0.420	1.00	15.00	A	O
ATOM	233	HN	SER	A	132	5.533	-6.034	-1.392	1.00	15.00	A	H

ATOM	234	HG	SER	A	132	5.760	-4.337	-0.258	1.00	15.00	A	H
ATOM	235	N	ARG	A	133	8.983	-4.292	-1.464	1.00	15.00	A	N
ATOM	236	CA	ARG	A	133	10.236	-4.675	-0.827	1.00	15.00	A	C
ATOM	237	C	ARG	A	133	10.211	-4.324	0.661	1.00	15.00	A	C
ATOM	238	O	ARG	A	133	9.240	-3.746	1.152	1.00	15.00	A	O
ATOM	239	CB	ARG	A	133	11.158	-3.478	-1.078	1.00	15.00	A	C
ATOM	240	CG	ARG	A	133	11.570	-3.305	-2.534	1.00	15.00	A	C
ATOM	241	CD	ARG	A	133	12.579	-2.180	-2.697	1.00	15.00	A	C
ATOM	242	NE	ARG	A	133	13.093	-2.098	-4.064	1.00	15.00	A	N
ATOM	243	CZ	ARG	A	133	14.314	-1.666	-4.375	1.00	15.00	A	C
ATOM	244	NH1	ARG	A	133	15.150	-1.275	-3.422	1.00	15.00	A	N1+
ATOM	245	NH2	ARG	A	133	14.701	-1.633	-5.644	1.00	15.00	A	N
ATOM	246	HN	ARG	A	133	8.835	-3.351	-1.695	1.00	15.00	A	H
ATOM	247	HE	ARG	A	133	12.494	-2.377	-4.789	1.00	15.00	A	H
ATOM	248	HH11	ARG	A	133	16.104	-0.939	-3.671	1.00	15.00	A	H
ATOM	249	HH12	ARG	A	133	14.854	-1.303	-2.425	1.00	15.00	A	H
ATOM	250	HH21	ARG	A	133	15.655	-1.296	-5.887	1.00	15.00	A	H
ATOM	251	HH22	ARG	A	133	14.052	-1.942	-6.394	1.00	15.00	A	H
ATOM	252	N	ARG	A	134	11.277	-4.679	1.375	1.00	15.00	A	N
ATOM	253	CA	ARG	A	134	11.366	-4.405	2.808	1.00	15.00	A	C
ATOM	254	C	ARG	A	134	11.715	-2.945	3.086	1.00	15.00	A	C
ATOM	255	O	ARG	A	134	12.864	-2.529	2.933	1.00	15.00	A	O
ATOM	256	CB	ARG	A	134	12.463	-3.718	3.628	1.00	15.00	A	C
ATOM	257	CG	ARG	A	134	13.099	-4.608	4.683	1.00	15.00	A	C
ATOM	258	CD	ARG	A	134	13.892	-3.795	5.692	1.00	15.00	A	C
ATOM	259	NE	ARG	A	134	14.481	-4.641	6.728	1.00	15.00	A	N
ATOM	260	CZ	ARG	A	134	14.462	-4.350	8.028	1.00	15.00	A	C
ATOM	261	NH1	ARG	A	134	13.886	-3.232	8.455	1.00	15.00	A	N1+
ATOM	262	NH2	ARG	A	134	15.022	-5.176	8.899	1.00	15.00	A	N
ATOM	263	HN	ARG	A	134	12.019	-5.138	0.930	1.00	15.00	A	H
ATOM	264	HE	ARG	A	134	14.915	-5.472	6.439	1.00	15.00	A	H
ATOM	265	HH11	ARG	A	134	13.445	-2.580	7.776	1.00	15.00	A	H
ATOM	266	HH12	ARG	A	134	13.875	-3.009	9.471	1.00	15.00	A	H
ATOM	267	HH21	ARG	A	134	15.008	-4.948	9.915	1.00	15.00	A	H
ATOM	268	HH22	ARG	A	134	15.475	-6.052	8.570	1.00	15.00	A	H
ATOM	269	N	ILE	A	135	10.712	-2.170	3.493	1.00	15.00	A	N
ATOM	270	CA	ILE	A	135	10.905	-0.757	3.803	1.00	15.00	A	C
ATOM	271	C	ILE	A	135	10.510	-0.472	5.252	1.00	15.00	A	C
ATOM	272	O	ILE	A	135	9.453	-0.904	5.713	1.00	15.00	A	O
ATOM	273	CB	ILE	A	135	10.096	0.153	2.850	1.00	15.00	A	C
ATOM	274	CG1	ILE	A	135	10.424	-0.178	1.390	1.00	15.00	A	C
ATOM	275	CG2	ILE	A	135	10.382	1.621	3.138	1.00	15.00	A	C
ATOM	276	CD1	ILE	A	135	9.558	0.548	0.383	1.00	15.00	A	C
ATOM	277	HN	ILE	A	135	9.818	-2.560	3.586	1.00	15.00	A	H
ATOM	278	N	VAL	A	136	11.370	0.253	5.966	1.00	15.00	A	N
ATOM	279	CA	VAL	A	136	11.123	0.584	7.370	1.00	15.00	A	C
ATOM	280	C	VAL	A	136	10.059	1.670	7.528	1.00	15.00	A	C

ATOM	281	O	VAL	A	136	10.213	2.786	7.030	1.00	15.00	A	O
ATOM	282	CB	VAL	A	136	12.417	1.028	8.085	1.00	15.00	A	C
ATOM	283	CG1	VAL	A	136	12.165	1.252	9.570	1.00	15.00	A	C
ATOM	284	CG2	VAL	A	136	13.524	0.005	7.878	1.00	15.00	A	C
ATOM	285	HN	VAL	A	136	12.192	0.573	5.538	1.00	15.00	A	H
ATOM	286	N	GLY	A	137	8.977	1.327	8.229	1.00	15.00	A	N
ATOM	287	CA	GLY	A	137	7.900	2.277	8.463	1.00	15.00	A	C
ATOM	288	C	GLY	A	137	6.939	2.391	7.295	1.00	15.00	A	C
ATOM	289	O	GLY	A	137	6.124	3.311	7.247	1.00	15.00	A	O
ATOM	290	HN	GLY	A	137	8.910	0.419	8.591	1.00	15.00	A	H
ATOM	291	N	GLN	A	138	7.030	1.457	6.354	1.00	15.00	A	N
ATOM	292	CA	GLN	A	138	6.159	1.466	5.184	1.00	15.00	A	C
ATOM	293	C	GLN	A	138	4.739	1.041	5.543	1.00	15.00	A	C
ATOM	294	O	GLN	A	138	3.771	1.539	4.969	1.00	15.00	A	O
ATOM	295	CB	GLN	A	138	6.719	0.572	4.077	1.00	15.00	A	C
ATOM	296	CG	GLN	A	138	5.938	0.649	2.775	1.00	15.00	A	C
ATOM	297	CD	GLN	A	138	5.832	2.064	2.231	1.00	15.00	A	C
ATOM	298	NE2	GLN	A	138	4.691	2.374	1.624	1.00	15.00	A	N
ATOM	299	OE1	GLN	A	138	6.756	2.869	2.363	1.00	15.00	A	O
ATOM	300	HN	GLN	A	138	7.700	0.749	6.445	1.00	15.00	A	H
ATOM	301	HE21	GLN	A	138	3.999	1.682	1.562	1.00	15.00	A	H
ATOM	302	HE22	GLN	A	138	4.586	3.280	1.264	1.00	15.00	A	H
ATOM	303	N	LEU	A	139	4.629	0.116	6.495	1.00	15.00	A	N
ATOM	304	CA	LEU	A	139	3.332	-0.378	6.945	1.00	15.00	A	C
ATOM	305	C	LEU	A	139	2.520	0.749	7.570	1.00	15.00	A	C
ATOM	306	O	LEU	A	139	1.321	0.870	7.325	1.00	15.00	A	O
ATOM	307	CB	LEU	A	139	3.518	-1.509	7.960	1.00	15.00	A	C
ATOM	308	CG	LEU	A	139	2.240	-2.057	8.600	1.00	15.00	A	C
ATOM	309	CD1	LEU	A	139	1.427	-2.854	7.591	1.00	15.00	A	C
ATOM	310	CD2	LEU	A	139	2.568	-2.900	9.823	1.00	15.00	A	C
ATOM	311	HN	LEU	A	139	5.443	-0.240	6.908	1.00	15.00	A	H
ATOM	312	N	VAL	A	140	3.193	1.578	8.368	1.00	15.00	A	N
ATOM	313	CA	VAL	A	140	2.551	2.707	9.032	1.00	15.00	A	C
ATOM	314	C	VAL	A	140	2.062	3.730	8.007	1.00	15.00	A	C
ATOM	315	O	VAL	A	140	1.008	4.338	8.183	1.00	15.00	A	O
ATOM	316	CB	VAL	A	140	3.507	3.396	10.034	1.00	15.00	A	C
ATOM	317	CG1	VAL	A	140	2.771	4.450	10.851	1.00	15.00	A	C
ATOM	318	CG2	VAL	A	140	4.155	2.366	10.948	1.00	15.00	A	C
ATOM	319	HN	VAL	A	140	4.150	1.425	8.511	1.00	15.00	A	H
ATOM	320	N	LYS	A	141	2.833	3.907	6.934	1.00	15.00	A	N
ATOM	321	CA	LYS	A	141	2.475	4.844	5.875	1.00	15.00	A	C
ATOM	322	C	LYS	A	141	1.214	4.396	5.142	1.00	15.00	A	C
ATOM	323	O	LYS	A	141	0.349	5.212	4.835	1.00	15.00	A	O
ATOM	324	CB	LYS	A	141	3.627	5.008	4.884	1.00	15.00	A	C
ATOM	325	CG	LYS	A	141	4.835	5.725	5.457	1.00	15.00	A	C
ATOM	326	CD	LYS	A	141	5.928	5.882	4.414	1.00	15.00	A	C
ATOM	327	CE	LYS	A	141	7.118	6.639	4.977	1.00	15.00	A	C

ATOM	328	NZ	LYS	A	141	8.158	6.882	3.942	1.00	15.00	A	N1+
ATOM	329	HN	LYS	A	141	3.666	3.396	6.856	1.00	15.00	A	H
ATOM	330	HZ1	LYS	A	141	7.752	7.409	3.142	1.00	15.00	A	H
ATOM	331	HZ2	LYS	A	141	8.534	5.978	3.591	1.00	15.00	A	H
ATOM	332	HZ3	LYS	A	141	8.942	7.436	4.346	1.00	15.00	A	H
ATOM	333	N	ILE	A	142	1.116	3.095	4.867	1.00	15.00	A	N
ATOM	334	CA	ILE	A	142	-0.048	2.542	4.174	1.00	15.00	A	C
ATOM	335	C	ILE	A	142	-1.302	2.662	5.039	1.00	15.00	A	C
ATOM	336	O	ILE	A	142	-2.384	2.962	4.535	1.00	15.00	A	O
ATOM	337	CB	ILE	A	142	0.166	1.069	3.759	1.00	15.00	A	C
ATOM	338	CG1	ILE	A	142	1.419	0.943	2.888	1.00	15.00	A	C
ATOM	339	CG2	ILE	A	142	-1.054	0.543	3.010	1.00	15.00	A	C
ATOM	340	CD1	ILE	A	142	1.849	-0.486	2.629	1.00	15.00	A	C
ATOM	341	HN	ILE	A	142	1.842	2.493	5.136	1.00	15.00	A	H
ATOM	342	N	ILE	A	143	-1.145	2.438	6.344	1.00	15.00	A	N
ATOM	343	CA	ILE	A	143	-2.264	2.536	7.276	1.00	15.00	A	C
ATOM	344	C	ILE	A	143	-2.747	3.983	7.396	1.00	15.00	A	C
ATOM	345	O	ILE	A	143	-3.948	4.237	7.452	1.00	15.00	A	O
ATOM	346	CB	ILE	A	143	-1.914	1.967	8.669	1.00	15.00	A	C
ATOM	347	CG1	ILE	A	143	-1.570	0.478	8.552	1.00	15.00	A	C
ATOM	348	CG2	ILE	A	143	-3.070	2.169	9.644	1.00	15.00	A	C
ATOM	349	CD1	ILE	A	143	-1.050	-0.140	9.833	1.00	15.00	A	C
ATOM	350	HN	ILE	A	143	-0.257	2.199	6.686	1.00	15.00	A	H
ATOM	351	N	SER	A	144	-1.802	4.926	7.418	1.00	15.00	A	N
ATOM	352	CA	SER	A	144	-2.135	6.347	7.508	1.00	15.00	A	C
ATOM	353	C	SER	A	144	-2.877	6.820	6.258	1.00	15.00	A	C
ATOM	354	O	SER	A	144	-3.740	7.694	6.334	1.00	15.00	A	O
ATOM	355	CB	SER	A	144	-0.879	7.190	7.725	1.00	15.00	A	C
ATOM	356	OG	SER	A	144	-0.243	6.854	8.943	1.00	15.00	A	O
ATOM	357	HN	SER	A	144	-0.859	4.660	7.384	1.00	15.00	A	H
ATOM	358	HG	SER	A	144	0.655	7.194	8.939	1.00	15.00	A	H
ATOM	359	N	VAL	A	145	-2.533	6.239	5.106	1.00	15.00	A	N
ATOM	360	CA	VAL	A	145	-3.182	6.589	3.846	1.00	15.00	A	C
ATOM	361	C	VAL	A	145	-4.609	6.042	3.821	1.00	15.00	A	C
ATOM	362	O	VAL	A	145	-5.528	6.708	3.348	1.00	15.00	A	O
ATOM	363	CB	VAL	A	145	-2.394	6.055	2.625	1.00	15.00	A	C
ATOM	364	CG1	VAL	A	145	-3.186	6.237	1.338	1.00	15.00	A	C
ATOM	365	CG2	VAL	A	145	-1.049	6.757	2.510	1.00	15.00	A	C
ATOM	366	HN	VAL	A	145	-1.827	5.558	5.106	1.00	15.00	A	H
ATOM	367	N	LEU	A	146	-4.783	4.830	4.349	1.00	15.00	A	N
ATOM	368	CA	LEU	A	146	-6.096	4.195	4.401	1.00	15.00	A	C
ATOM	369	C	LEU	A	146	-7.042	4.954	5.326	1.00	15.00	A	C
ATOM	370	O	LEU	A	146	-8.181	5.230	4.961	1.00	15.00	A	O
ATOM	371	CB	LEU	A	146	-5.977	2.733	4.844	1.00	15.00	A	C
ATOM	372	CG	LEU	A	146	-5.298	1.783	3.855	1.00	15.00	A	C
ATOM	373	CD1	LEU	A	146	-5.034	0.430	4.499	1.00	15.00	A	C
ATOM	374	CD2	LEU	A	146	-6.139	1.625	2.597	1.00	15.00	A	C

ATOM	375	HN	LEU	A	146	-4.009	4.350	4.709	1.00	15.00	A	H
ATOM	376	N	GLU	A	147	-6.558	5.301	6.521	1.00	15.00	A	N
ATOM	377	CA	GLU	A	147	-7.363	6.037	7.498	1.00	15.00	A	C
ATOM	378	C	GLU	A	147	-7.737	7.426	6.979	1.00	15.00	A	C
ATOM	379	O	GLU	A	147	-8.803	7.951	7.303	1.00	15.00	A	O
ATOM	380	CB	GLU	A	147	-6.624	6.156	8.833	1.00	15.00	A	C
ATOM	381	CG	GLU	A	147	-6.308	4.823	9.492	1.00	15.00	A	C
ATOM	382	CD	GLU	A	147	-5.467	4.973	10.746	1.00	15.00	A	C
ATOM	383	OE1	GLU	A	147	-5.746	4.261	11.733	1.00	15.00	A	O
ATOM	384	OE2	GLU	A	147	-4.532	5.802	10.740	1.00	15.00	A	O1-
ATOM	385	HN	GLU	A	147	-5.637	5.053	6.752	1.00	15.00	A	H
ATOM	386	N	LYS	A	148	-6.850	8.009	6.173	1.00	15.00	A	N
ATOM	387	CA	LYS	A	148	-7.076	9.330	5.592	1.00	15.00	A	C
ATOM	388	C	LYS	A	148	-8.134	9.274	4.486	1.00	15.00	A	C
ATOM	389	O	LYS	A	148	-8.831	10.258	4.232	1.00	15.00	A	O
ATOM	390	CB	LYS	A	148	-5.761	9.889	5.037	1.00	15.00	A	C
ATOM	391	CG	LYS	A	148	-5.861	11.292	4.459	1.00	15.00	A	C
ATOM	392	CD	LYS	A	148	-4.602	11.660	3.693	1.00	15.00	A	C
ATOM	393	CE	LYS	A	148	-4.767	12.979	2.956	1.00	15.00	A	C
ATOM	394	NZ	LYS	A	148	-3.570	13.308	2.134	1.00	15.00	A	N1+
ATOM	395	HN	LYS	A	148	-6.015	7.540	5.969	1.00	15.00	A	H
ATOM	396	HZ1	LYS	A	148	-3.391	12.552	1.443	1.00	15.00	A	H
ATOM	397	HZ2	LYS	A	148	-2.734	13.410	2.744	1.00	15.00	A	H
ATOM	398	HZ3	LYS	A	148	-3.721	14.201	1.622	1.00	15.00	A	H
ATOM	399	N	LEU	A	149	-8.252	8.114	3.835	1.00	15.00	A	N
ATOM	400	CA	LEU	A	149	-9.225	7.931	2.756	1.00	15.00	A	C
ATOM	401	C	LEU	A	149	-10.536	7.342	3.281	1.00	15.00	A	C
ATOM	402	O	LEU	A	149	-11.366	6.874	2.499	1.00	15.00	A	O
ATOM	403	CB	LEU	A	149	-8.653	7.037	1.650	1.00	15.00	A	C
ATOM	404	CG	LEU	A	149	-7.342	7.495	1.002	1.00	15.00	A	C
ATOM	405	CD1	LEU	A	149	-6.950	6.562	-0.133	1.00	15.00	A	C
ATOM	406	CD2	LEU	A	149	-7.444	8.933	0.515	1.00	15.00	A	C
ATOM	407	HN	LEU	A	149	-7.676	7.363	4.088	1.00	15.00	A	H
ATOM	408	N	SER	A	150	-10.703	7.368	4.607	1.00	15.00	A	N
ATOM	409	CA	SER	A	150	-11.906	6.852	5.269	1.00	15.00	A	C
ATOM	410	C	SER	A	150	-12.034	5.328	5.169	1.00	15.00	A	C
ATOM	411	O	SER	A	150	-13.139	4.797	5.058	1.00	15.00	A	O
ATOM	412	CB	SER	A	150	-13.174	7.546	4.754	1.00	15.00	A	C
ATOM	413	OG	SER	A	150	-13.092	8.952	4.921	1.00	15.00	A	O
ATOM	414	HN	SER	A	150	-9.990	7.750	5.164	1.00	15.00	A	H
ATOM	415	HG	SER	A	150	-13.694	9.227	5.618	1.00	15.00	A	H
ATOM	416	N	PHE	A	151	-10.904	4.629	5.220	1.00	15.00	A	N
ATOM	417	CA	PHE	A	151	-10.899	3.169	5.155	1.00	15.00	A	C
ATOM	418	C	PHE	A	151	-10.561	2.562	6.511	1.00	15.00	A	C
ATOM	419	O	PHE	A	151	-9.555	2.917	7.127	1.00	15.00	A	O
ATOM	420	CB	PHE	A	151	-9.911	2.664	4.101	1.00	15.00	A	C
ATOM	421	CG	PHE	A	151	-10.423	2.755	2.695	1.00	15.00	A	C

```
ATOM    422  CD1 PHE A 151     -11.311   1.811   2.205  1.00 15.00      A    C
ATOM    423  CD2 PHE A 151     -10.015   3.781   1.863  1.00 15.00      A    C
ATOM    424  CE1 PHE A 151     -11.783   1.890   0.908  1.00 15.00      A    C
ATOM    425  CE2 PHE A 151     -10.481   3.868   0.565  1.00 15.00      A    C
ATOM    426  CZ  PHE A 151     -11.367   2.920   0.087  1.00 15.00      A    C
ATOM    427  HN  PHE A 151     -10.050   5.105   5.297  1.00 15.00      A    H
ATOM    428  N   GLN A 152     -11.405   1.646   6.970  1.00 15.00      A    N
ATOM    429  CA  GLN A 152     -11.190   0.988   8.251  1.00 15.00      A    C
ATOM    430  C   GLN A 152     -10.563  -0.390   8.056  1.00 15.00      A    C
ATOM    431  O   GLN A 152     -11.136  -1.255   7.395  1.00 15.00      A    O
ATOM    432  CB  GLN A 152     -12.506   0.864   9.024  1.00 15.00      A    C
ATOM    433  CG  GLN A 152     -12.380   0.128  10.350  1.00 15.00      A    C
ATOM    434  CD  GLN A 152     -13.704  -0.024  11.068  1.00 15.00      A    C
ATOM    435  NE2 GLN A 152     -13.647  -0.087  12.395  1.00 15.00      A    N
ATOM    436  OE1 GLN A 152     -14.764  -0.088  10.442  1.00 15.00      A    O
ATOM    437  HN  GLN A 152     -12.189   1.403   6.431  1.00 15.00      A    H
ATOM    438 HE21 GLN A 152     -12.766  -0.034  12.822  1.00 15.00      A    H
ATOM    439 HE22 GLN A 152     -14.486  -0.186  12.892  1.00 15.00      A    H
ATOM    440  N   VAL A 153      -9.385  -0.583   8.637  1.00 15.00      A    N
ATOM    441  CA  VAL A 153      -8.682  -1.856   8.537  1.00 15.00      A    C
ATOM    442  C   VAL A 153      -9.304  -2.890   9.471  1.00 15.00      A    C
ATOM    443  O   VAL A 153      -9.299  -2.718  10.690  1.00 15.00      A    O
ATOM    444  CB  VAL A 153      -7.184  -1.704   8.877  1.00 15.00      A    C
ATOM    445  CG1 VAL A 153      -6.430  -2.996   8.600  1.00 15.00      A    C
ATOM    446  CG2 VAL A 153      -6.568  -0.546   8.104  1.00 15.00      A    C
ATOM    447  HN  VAL A 153      -8.977   0.149   9.142  1.00 15.00      A    H
ATOM    448  N   LEU A 154      -9.849  -3.956   8.894  1.00 15.00      A    N
ATOM    449  CA  LEU A 154     -10.470  -5.016   9.683  1.00 15.00      A    C
ATOM    450  C   LEU A 154      -9.485  -6.157   9.937  1.00 15.00      A    C
ATOM    451  O   LEU A 154      -9.447  -6.724  11.029  1.00 15.00      A    O
ATOM    452  CB  LEU A 154     -11.724  -5.552   8.987  1.00 15.00      A    C
ATOM    453  CG  LEU A 154     -12.739  -4.513   8.501  1.00 15.00      A    C
ATOM    454  CD1 LEU A 154     -13.753  -5.159   7.570  1.00 15.00      A    C
ATOM    455  CD2 LEU A 154     -13.439  -3.846   9.677  1.00 15.00      A    C
ATOM    456  HN  LEU A 154      -9.834  -4.032   7.918  1.00 15.00      A    H
ATOM    457  N   HIS A 155      -8.685  -6.482   8.920  1.00 15.00      A    N
ATOM    458  CA  HIS A 155      -7.698  -7.553   9.031  1.00 15.00      A    C
ATOM    459  C   HIS A 155      -6.397  -7.181   8.319  1.00 15.00      A    C
ATOM    460  O   HIS A 155      -6.410  -6.519   7.280  1.00 15.00      A    O
ATOM    461  CB  HIS A 155      -8.265  -8.868   8.475  1.00 15.00      A    C
ATOM    462  CG  HIS A 155      -7.354 -10.052   8.632  1.00 15.00      A    C
ATOM    463  CD2 HIS A 155      -7.141 -10.840   9.717  1.00 15.00      A    C
ATOM    464  ND1 HIS A 155      -6.571 -10.496   7.592  1.00 15.00      A    N
ATOM    465  CE1 HIS A 155      -5.903 -11.534   8.058  1.00 15.00      A    C
ATOM    466  NE2 HIS A 155      -6.215 -11.780   9.338  1.00 15.00      A    N
ATOM    467  HN  HIS A 155      -8.761  -5.988   8.077  1.00 15.00      A    H
ATOM    468  HE2 HIS A 155      -5.856 -12.499   9.898  1.00 15.00      A    H
```

```
ATOM    469  N   LEU A 156      -5.277  -7.613   8.894  1.00 15.00      A    N
ATOM    470  CA  LEU A 156      -3.963  -7.337   8.326  1.00 15.00      A    C
ATOM    471  C   LEU A 156      -3.026  -8.536   8.488  1.00 15.00      A    C
ATOM    472  O   LEU A 156      -2.764  -8.990   9.603  1.00 15.00      A    O
ATOM    473  CB  LEU A 156      -3.360  -6.078   8.966  1.00 15.00      A    C
ATOM    474  CG  LEU A 156      -1.860  -5.830   8.749  1.00 15.00      A    C
ATOM    475  CD1 LEU A 156      -1.513  -5.796   7.265  1.00 15.00      A    C
ATOM    476  CD2 LEU A 156      -1.433  -4.535   9.427  1.00 15.00      A    C
ATOM    477  HN  LEU A 156      -5.338  -8.130   9.723  1.00 15.00      A    H
ATOM    478  N   ASN A 157      -2.532  -9.042   7.361  1.00 15.00      A    N
ATOM    479  CA  ASN A 157      -1.619 -10.182   7.352  1.00 15.00      A    C
ATOM    480  C   ASN A 157      -0.309  -9.806   6.657  1.00 15.00      A    C
ATOM    481  O   ASN A 157      -0.310  -9.389   5.500  1.00 15.00      A    O
ATOM    482  CB  ASN A 157      -2.275 -11.383   6.658  1.00 15.00      A    C
ATOM    483  CG  ASN A 157      -1.353 -12.583   6.533  1.00 15.00      A    C
ATOM    484  ND2 ASN A 157      -1.278 -13.388   7.588  1.00 15.00      A    N
ATOM    485  OD1 ASN A 157      -0.716 -12.785   5.497  1.00 15.00      A    O
ATOM    486  HN  ASN A 157      -2.784  -8.630   6.506  1.00 15.00      A    H
ATOM    487 HD21 ASN A 157      -1.813 -13.167   8.379  1.00 15.00      A    H
ATOM    488 HD22 ASN A 157      -0.689 -14.171   7.534  1.00 15.00      A    H
ATOM    489  N   ILE A 158       0.806  -9.954   7.372  1.00 15.00      A    N
ATOM    490  CA  ILE A 158       2.119  -9.612   6.825  1.00 15.00      A    C
ATOM    491  C   ILE A 158       2.986 -10.851   6.594  1.00 15.00      A    C
ATOM    492  O   ILE A 158       3.418 -11.506   7.542  1.00 15.00      A    O
ATOM    493  CB  ILE A 158       2.879  -8.633   7.748  1.00 15.00      A    C
ATOM    494  CG1 ILE A 158       2.015  -7.409   8.067  1.00 15.00      A    C
ATOM    495  CG2 ILE A 158       4.195  -8.204   7.111  1.00 15.00      A    C
ATOM    496  CD1 ILE A 158       2.540  -6.579   9.218  1.00 15.00      A    C
ATOM    497  HN  ILE A 158       0.744 -10.302   8.286  1.00 15.00      A    H
ATOM    498  N   SER A 159       3.238 -11.160   5.326  1.00 15.00      A    N
ATOM    499  CA  SER A 159       4.069 -12.304   4.961  1.00 15.00      A    C
ATOM    500  C   SER A 159       5.314 -11.838   4.212  1.00 15.00      A    C
ATOM    501  O   SER A 159       5.213 -11.161   3.192  1.00 15.00      A    O
ATOM    502  CB  SER A 159       3.278 -13.290   4.098  1.00 15.00      A    C
ATOM    503  OG  SER A 159       2.136 -13.770   4.786  1.00 15.00      A    O
ATOM    504  HN  SER A 159       2.851 -10.605   4.615  1.00 15.00      A    H
ATOM    505  HG  SER A 159       2.357 -13.927   5.705  1.00 15.00      A    H
ATOM    506  N   SER A 160       6.490 -12.196   4.717  1.00 15.00      A    N
ATOM    507  CA  SER A 160       7.739 -11.788   4.081  1.00 15.00      A    C
ATOM    508  C   SER A 160       8.515 -12.973   3.513  1.00 15.00      A    C
ATOM    509  O   SER A 160       8.561 -14.048   4.113  1.00 15.00      A    O
ATOM    510  CB  SER A 160       8.613 -10.995   5.058  1.00 15.00      A    C
ATOM    511  OG  SER A 160       8.953 -11.776   6.190  1.00 15.00      A    O
ATOM    512  HN  SER A 160       6.520 -12.745   5.529  1.00 15.00      A    H
ATOM    513  HG  SER A 160       9.108 -12.683   5.920  1.00 15.00      A    H
ATOM    514  N   MET A 161       9.125 -12.760   2.349  1.00 15.00      A    N
ATOM    515  CA  MET A 161       9.916 -13.790   1.685  1.00 15.00      A    C
```

```
ATOM    516  C   MET A 161      11.138 -13.172   1.008  1.00 15.00      A    C
ATOM    517  O   MET A 161      11.003 -12.416   0.045  1.00 15.00      A    O
ATOM    518  CB  MET A 161       9.069 -14.544   0.654  1.00 15.00      A    C
ATOM    519  CG  MET A 161       9.737 -15.792   0.096  1.00 15.00      A    C
ATOM    520  SD  MET A 161       8.844 -16.498  -1.303  1.00 15.00      A    S
ATOM    521  CE  MET A 161       9.066 -15.212  -2.528  1.00 15.00      A    C
ATOM    522  HN  MET A 161       9.038 -11.881   1.923  1.00 15.00      A    H
ATOM    523  N   GLU A 162      12.323 -13.505   1.527  1.00 15.00      A    N
ATOM    524  CA  GLU A 162      13.592 -12.998   0.997  1.00 15.00      A    C
ATOM    525  C   GLU A 162      13.783 -11.494   1.197  1.00 15.00      A    C
ATOM    526  O   GLU A 162      14.153 -11.047   2.284  1.00 15.00      A    O
ATOM    527  CB  GLU A 162      14.795 -12.196   1.510  1.00 15.00      A    C
ATOM    528  CG  GLU A 162      15.613 -12.905   2.577  1.00 15.00      A    C
ATOM    529  CD  GLU A 162      16.841 -12.114   2.981  1.00 15.00      A    C
ATOM    530  OE1 GLU A 162      17.894 -12.279   2.329  1.00 15.00      A    O
ATOM    531  OE2 GLU A 162      16.750 -11.327   3.947  1.00 15.00      A    O1-
ATOM    532  HN  GLU A 162      12.344 -14.114   2.293  1.00 15.00      A    H
ATOM    533  N   GLU A 163      13.525 -10.719   0.147  1.00 15.00      A    N
ATOM    534  CA  GLU A 163      13.676  -9.269   0.208  1.00 15.00      A    C
ATOM    535  C   GLU A 163      12.356  -8.554  -0.074  1.00 15.00      A    C
ATOM    536  O   GLU A 163      12.297  -7.325  -0.061  1.00 15.00      A    O
ATOM    537  CB  GLU A 163      14.742  -8.802  -0.790  1.00 15.00      A    C
ATOM    538  CG  GLU A 163      16.139  -9.343  -0.521  1.00 15.00      A    C
ATOM    539  CD  GLU A 163      17.161  -8.850  -1.529  1.00 15.00      A    C
ATOM    540  OE1 GLU A 163      17.214  -9.410  -2.645  1.00 15.00      A    O
ATOM    541  OE2 GLU A 163      17.908  -7.903  -1.203  1.00 15.00      A    O1-
ATOM    542  HN  GLU A 163      13.228 -11.132  -0.690  1.00 15.00      A    H
ATOM    543  N   THR A 164      11.297  -9.323  -0.325  1.00 15.00      A    N
ATOM    544  CA  THR A 164       9.988  -8.744  -0.621  1.00 15.00      A    C
ATOM    545  C   THR A 164       9.007  -8.906   0.538  1.00 15.00      A    C
ATOM    546  O   THR A 164       9.105  -9.851   1.319  1.00 15.00      A    O
ATOM    547  CB  THR A 164       9.369  -9.361  -1.889  1.00 15.00      A    C
ATOM    548  CG2 THR A 164      10.231  -9.065  -3.107  1.00 15.00      A    C
ATOM    549  OG1 THR A 164       9.240 -10.781  -1.727  1.00 15.00      A    O
ATOM    550  HN  THR A 164      11.398 -10.297  -0.313  1.00 15.00      A    H
ATOM    551  HG1 THR A 164       8.900 -10.976  -0.849  1.00 15.00      A    H
ATOM    552  N   VAL A 165       8.067  -7.965   0.640  1.00 15.00      A    N
ATOM    553  CA  VAL A 165       7.046  -7.990   1.687  1.00 15.00      A    C
ATOM    554  C   VAL A 165       5.656  -7.775   1.083  1.00 15.00      A    C
ATOM    555  O   VAL A 165       5.434  -6.825   0.330  1.00 15.00      A    O
ATOM    556  CB  VAL A 165       7.293  -6.918   2.775  1.00 15.00      A    C
ATOM    557  CG1 VAL A 165       6.277  -7.051   3.898  1.00 15.00      A    C
ATOM    558  CG2 VAL A 165       8.704  -7.019   3.330  1.00 15.00      A    C
ATOM    559  HN  VAL A 165       8.058  -7.232  -0.011  1.00 15.00      A    H
ATOM    560  N   LEU A 166       4.730  -8.665   1.425  1.00 15.00      A    N
ATOM    561  CA  LEU A 166       3.363  -8.598   0.928  1.00 15.00      A    C
ATOM    562  C   LEU A 166       2.415  -8.053   1.999  1.00 15.00      A    C
```

ATOM	563	O	LEU	A	166	2.266	-8.640	3.071	1.00	15.00	A	O
ATOM	564	CB	LEU	A	166	2.912	-9.993	0.476	1.00	15.00	A	C
ATOM	565	CG	LEU	A	166	1.417	-10.187	0.204	1.00	15.00	A	C
ATOM	566	CD1	LEU	A	166	0.967	-9.352	-0.987	1.00	15.00	A	C
ATOM	567	CD2	LEU	A	166	1.105	-11.658	-0.019	1.00	15.00	A	C
ATOM	568	HN	LEU	A	166	4.976	-9.390	2.035	1.00	15.00	A	H
ATOM	569	N	TYR	A	167	1.778	-6.926	1.693	1.00	15.00	A	N
ATOM	570	CA	TYR	A	167	0.848	-6.291	2.619	1.00	15.00	A	C
ATOM	571	C	TYR	A	167	-0.604	-6.556	2.216	1.00	15.00	A	C
ATOM	572	O	TYR	A	167	-1.129	-5.931	1.293	1.00	15.00	A	O
ATOM	573	CB	TYR	A	167	1.102	-4.783	2.680	1.00	15.00	A	C
ATOM	574	CG	TYR	A	167	2.472	-4.390	3.194	1.00	15.00	A	C
ATOM	575	CD1	TYR	A	167	2.798	-4.523	4.539	1.00	15.00	A	C
ATOM	576	CD2	TYR	A	167	3.435	-3.877	2.333	1.00	15.00	A	C
ATOM	577	CE1	TYR	A	167	4.045	-4.154	5.011	1.00	15.00	A	C
ATOM	578	CE2	TYR	A	167	4.684	-3.508	2.796	1.00	15.00	A	C
ATOM	579	CZ	TYR	A	167	4.984	-3.649	4.135	1.00	15.00	A	C
ATOM	580	OH	TYR	A	167	6.226	-3.280	4.599	1.00	15.00	A	O
ATOM	581	HN	TYR	A	167	1.941	-6.509	0.820	1.00	15.00	A	H
ATOM	582	HH	TYR	A	167	6.472	-2.435	4.218	1.00	15.00	A	H
ATOM	583	N	PHE	A	168	-1.246	-7.482	2.919	1.00	15.00	A	N
ATOM	584	CA	PHE	A	168	-2.636	-7.831	2.648	1.00	15.00	A	C
ATOM	585	C	PHE	A	168	-3.573	-6.986	3.510	1.00	15.00	A	C
ATOM	586	O	PHE	A	168	-3.559	-7.084	4.736	1.00	15.00	A	O
ATOM	587	CB	PHE	A	168	-2.866	-9.325	2.907	1.00	15.00	A	C
ATOM	588	CG	PHE	A	168	-4.202	-9.837	2.447	1.00	15.00	A	C
ATOM	589	CD1	PHE	A	168	-5.188	-10.159	3.365	1.00	15.00	A	C
ATOM	590	CD2	PHE	A	168	-4.469	-10.001	1.097	1.00	15.00	A	C
ATOM	591	CE1	PHE	A	168	-6.417	-10.635	2.947	1.00	15.00	A	C
ATOM	592	CE2	PHE	A	168	-5.694	-10.475	0.672	1.00	15.00	A	C
ATOM	593	CZ	PHE	A	168	-6.669	-10.793	1.598	1.00	15.00	A	C
ATOM	594	HN	PHE	A	168	-0.771	-7.941	3.642	1.00	15.00	A	H
ATOM	595	N	PHE	A	169	-4.385	-6.158	2.860	1.00	15.00	A	N
ATOM	596	CA	PHE	A	169	-5.315	-5.283	3.567	1.00	15.00	A	C
ATOM	597	C	PHE	A	169	-6.770	-5.560	3.208	1.00	15.00	A	C
ATOM	598	O	PHE	A	169	-7.112	-5.750	2.043	1.00	15.00	A	O
ATOM	599	CB	PHE	A	169	-5.008	-3.811	3.269	1.00	15.00	A	C
ATOM	600	CG	PHE	A	169	-3.735	-3.295	3.874	1.00	15.00	A	C
ATOM	601	CD1	PHE	A	169	-3.717	-2.798	5.167	1.00	15.00	A	C
ATOM	602	CD2	PHE	A	169	-2.560	-3.295	3.142	1.00	15.00	A	C
ATOM	603	CE1	PHE	A	169	-2.548	-2.310	5.720	1.00	15.00	A	C
ATOM	604	CE2	PHE	A	169	-1.388	-2.810	3.690	1.00	15.00	A	C
ATOM	605	CZ	PHE	A	169	-1.381	-2.318	4.981	1.00	15.00	A	C
ATOM	606	HN	PHE	A	169	-4.359	-6.134	1.881	1.00	15.00	A	H
ATOM	607	N	VAL	A	170	-7.614	-5.589	4.231	1.00	15.00	A	N
ATOM	608	CA	VAL	A	170	-9.050	-5.781	4.055	1.00	15.00	A	C
ATOM	609	C	VAL	A	170	-9.746	-4.547	4.625	1.00	15.00	A	C

ATOM	610	O	VAL	A	170	-10.171	-4.530	5.779	1.00	15.00	A	O
ATOM	611	CB	VAL	A	170	-9.560	-7.047	4.781	1.00	15.00	A	C
ATOM	612	CG1	VAL	A	170	-11.043	-7.271	4.507	1.00	15.00	A	C
ATOM	613	CG2	VAL	A	170	-8.756	-8.267	4.356	1.00	15.00	A	C
ATOM	614	HN	VAL	A	170	-7.260	-5.474	5.139	1.00	15.00	A	H
ATOM	615	N	VAL	A	171	-9.829	-3.503	3.812	1.00	15.00	A	N
ATOM	616	CA	VAL	A	171	-10.417	-2.238	4.242	1.00	15.00	A	C
ATOM	617	C	VAL	A	171	-11.896	-2.084	3.903	1.00	15.00	A	C
ATOM	618	O	VAL	A	171	-12.408	-2.685	2.961	1.00	15.00	A	O
ATOM	619	CB	VAL	A	171	-9.643	-1.030	3.669	1.00	15.00	A	C
ATOM	620	CG1	VAL	A	171	-8.263	-0.934	4.294	1.00	15.00	A	C
ATOM	621	CG2	VAL	A	171	-9.536	-1.119	2.153	1.00	15.00	A	C
ATOM	622	HN	VAL	A	171	-9.490	-3.586	2.898	1.00	15.00	A	H
ATOM	623	N	LYS	A	172	-12.565	-1.261	4.702	1.00	15.00	A	N
ATOM	624	CA	LYS	A	172	-13.975	-0.949	4.516	1.00	15.00	A	C
ATOM	625	C	LYS	A	172	-14.082	0.470	3.963	1.00	15.00	A	C
ATOM	626	O	LYS	A	172	-13.425	1.385	4.465	1.00	15.00	A	O
ATOM	627	CB	LYS	A	172	-14.710	-1.044	5.861	1.00	15.00	A	C
ATOM	628	CG	LYS	A	172	-16.179	-0.650	5.818	1.00	15.00	A	C
ATOM	629	CD	LYS	A	172	-16.767	-0.568	7.220	1.00	15.00	A	C
ATOM	630	CE	LYS	A	172	-18.220	-0.118	7.194	1.00	15.00	A	C
ATOM	631	NZ	LYS	A	172	-18.799	-0.016	8.563	1.00	15.00	A	N1+
ATOM	632	HN	LYS	A	172	-12.089	-0.848	5.454	1.00	15.00	A	H
ATOM	633	HZ1	LYS	A	172	-18.257	0.668	9.131	1.00	15.00	A	H
ATOM	634	HZ2	LYS	A	172	-18.770	-0.941	9.036	1.00	15.00	A	H
ATOM	635	HZ3	LYS	A	172	-19.789	0.302	8.511	1.00	15.00	A	H
ATOM	636	N	ILE	A	173	-14.892	0.648	2.924	1.00	15.00	A	N
ATOM	637	CA	ILE	A	173	-15.064	1.961	2.303	1.00	15.00	A	C
ATOM	638	C	ILE	A	173	-15.872	2.901	3.196	1.00	15.00	A	C
ATOM	639	O	ILE	A	173	-16.999	2.591	3.585	1.00	15.00	A	O
ATOM	640	CB	ILE	A	173	-15.756	1.853	0.926	1.00	15.00	A	C
ATOM	641	CG1	ILE	A	173	-15.089	0.771	0.073	1.00	15.00	A	C
ATOM	642	CG2	ILE	A	173	-15.724	3.195	0.205	1.00	15.00	A	C
ATOM	643	CD1	ILE	A	173	-15.862	0.424	-1.183	1.00	15.00	A	C
ATOM	644	HN	ILE	A	173	-15.384	-0.119	2.566	1.00	15.00	A	H
ATOM	645	N	GLY	A	174	-15.283	4.052	3.517	1.00	15.00	A	N
ATOM	646	CA	GLY	A	174	-15.959	5.030	4.352	1.00	15.00	A	C
ATOM	647	C	GLY	A	174	-17.026	5.794	3.588	1.00	15.00	A	C
ATOM	648	O	GLY	A	174	-17.065	5.756	2.357	1.00	15.00	A	O
ATOM	649	HN	GLY	A	174	-14.378	4.236	3.187	1.00	15.00	A	H
ATOM	650	N	LEU	A	175	-17.893	6.491	4.317	1.00	15.00	A	N
ATOM	651	CA	LEU	A	175	-18.971	7.262	3.701	1.00	15.00	A	C
ATOM	652	C	LEU	A	175	-18.454	8.548	3.059	1.00	15.00	A	C
ATOM	653	O	LEU	A	175	-19.128	9.145	2.220	1.00	15.00	A	O
ATOM	654	CB	LEU	A	175	-20.054	7.590	4.734	1.00	15.00	A	C
ATOM	655	CG	LEU	A	175	-20.729	6.394	5.409	1.00	15.00	A	C
ATOM	656	CD1	LEU	A	175	-21.643	6.858	6.533	1.00	15.00	A	C

ATOM	657	CD2	LEU	A	175	-21.505	5.568	4.393	1.00	15.00	A	C
ATOM	658	HN	LEU	A	175	-17.810	6.485	5.292	1.00	15.00	A	H
ATOM	659	N	GLU	A	176	-17.255	8.968	3.455	1.00	15.00	A	N
ATOM	660	CA	GLU	A	176	-16.659	10.187	2.916	1.00	15.00	A	C
ATOM	661	C	GLU	A	176	-15.627	9.885	1.829	1.00	15.00	A	C
ATOM	662	O	GLU	A	176	-14.865	10.763	1.426	1.00	15.00	A	O
ATOM	663	CB	GLU	A	176	-16.023	11.015	4.036	1.00	15.00	A	C
ATOM	664	CG	GLU	A	176	-16.995	11.410	5.137	1.00	15.00	A	C
ATOM	665	CD	GLU	A	176	-16.333	12.183	6.260	1.00	15.00	A	C
ATOM	666	OE1	GLU	A	176	-17.062	12.823	7.048	1.00	15.00	A	O
ATOM	667	OE2	GLU	A	176	-15.086	12.151	6.354	1.00	15.00	A	O1-
ATOM	668	HN	GLU	A	176	-16.762	8.449	4.124	1.00	15.00	A	H
ATOM	669	N	CYS	A	177	-15.611	8.642	1.351	1.00	15.00	A	N
ATOM	670	CA	CYS	A	177	-14.674	8.236	0.309	1.00	15.00	A	C
ATOM	671	C	CYS	A	177	-15.248	8.503	-1.081	1.00	15.00	A	C
ATOM	672	O	CYS	A	177	-16.292	7.964	-1.445	1.00	15.00	A	O
ATOM	673	CB	CYS	A	177	-14.304	6.757	0.454	1.00	15.00	A	C
ATOM	674	SG	CYS	A	177	-13.135	6.157	-0.790	1.00	15.00	A	S
ATOM	675	HN	CYS	A	177	-16.243	7.984	1.708	1.00	15.00	A	H
ATOM	676	HG	CYS	A	177	-12.163	5.511	-0.162	1.00	15.00	A	H
ATOM	677	N	HIS	A	178	-14.550	9.336	-1.850	1.00	15.00	A	N
ATOM	678	CA	HIS	A	178	-14.987	9.683	-3.201	1.00	15.00	A	C
ATOM	679	C	HIS	A	178	-13.955	9.245	-4.241	1.00	15.00	A	C
ATOM	680	O	HIS	A	178	-14.018	9.651	-5.402	1.00	15.00	A	O
ATOM	681	CB	HIS	A	178	-15.226	11.196	-3.312	1.00	15.00	A	C
ATOM	682	CG	HIS	A	178	-16.159	11.749	-2.273	1.00	15.00	A	C
ATOM	683	CD2	HIS	A	178	-15.882	12.516	-1.186	1.00	15.00	A	C
ATOM	684	ND1	HIS	A	178	-17.511	11.501	-2.317	1.00	15.00	A	N
ATOM	685	CE1	HIS	A	178	-18.027	12.115	-1.267	1.00	15.00	A	C
ATOM	686	NE2	HIS	A	178	-17.080	12.740	-0.555	1.00	15.00	A	N
ATOM	687	HN	HIS	A	178	-13.724	9.727	-1.501	1.00	15.00	A	H
ATOM	688	HE2	HIS	A	178	-17.217	13.259	0.267	1.00	15.00	A	H
ATOM	689	N	LEU	A	179	-13.006	8.416	-3.814	1.00	15.00	A	N
ATOM	690	CA	LEU	A	179	-11.956	7.929	-4.702	1.00	15.00	A	C
ATOM	691	C	LEU	A	179	-12.274	6.536	-5.236	1.00	15.00	A	C
ATOM	692	O	LEU	A	179	-12.938	5.739	-4.572	1.00	15.00	A	O
ATOM	693	CB	LEU	A	179	-10.608	7.922	-3.980	1.00	15.00	A	C
ATOM	694	CG	LEU	A	179	-10.116	9.279	-3.470	1.00	15.00	A	C
ATOM	695	CD1	LEU	A	179	-9.727	9.182	-2.005	1.00	15.00	A	C
ATOM	696	CD2	LEU	A	179	-8.942	9.769	-4.305	1.00	15.00	A	C
ATOM	697	HN	LEU	A	179	-13.015	8.122	-2.880	1.00	15.00	A	H
ATOM	698	N	SER	A	180	-11.789	6.254	-6.443	1.00	15.00	A	N
ATOM	699	CA	SER	A	180	-12.008	4.958	-7.077	1.00	15.00	A	C
ATOM	700	C	SER	A	180	-10.876	3.989	-6.742	1.00	15.00	A	C
ATOM	701	O	SER	A	180	-9.951	4.332	-6.003	1.00	15.00	A	O
ATOM	702	CB	SER	A	180	-12.129	5.120	-8.598	1.00	15.00	A	C
ATOM	703	OG	SER	A	180	-10.919	5.595	-9.164	1.00	15.00	A	O

```
ATOM    704  HN  SER A 180     -11.272   6.936  -6.920  1.00 15.00      A    H
ATOM    705  HG  SER A 180     -10.723   5.099  -9.962  1.00 15.00      A    H
ATOM    706  N   LEU A 181     -10.951   2.780  -7.295  1.00 15.00      A    N
ATOM    707  CA  LEU A 181      -9.928   1.764  -7.059  1.00 15.00      A    C
ATOM    708  C   LEU A 181      -8.627   2.129  -7.760  1.00 15.00      A    C
ATOM    709  O   LEU A 181      -7.543   1.764  -7.303  1.00 15.00      A    O
ATOM    710  CB  LEU A 181     -10.408   0.392  -7.535  1.00 15.00      A    C
ATOM    711  CG  LEU A 181     -11.612  -0.196  -6.801  1.00 15.00      A    C
ATOM    712  CD1 LEU A 181     -11.953  -1.567  -7.361  1.00 15.00      A    C
ATOM    713  CD2 LEU A 181     -11.345  -0.277  -5.305  1.00 15.00      A    C
ATOM    714  HN  LEU A 181     -11.710   2.566  -7.873  1.00 15.00      A    H
ATOM    715  N   GLU A 182      -8.743   2.849  -8.872  1.00 15.00      A    N
ATOM    716  CA  GLU A 182      -7.579   3.271  -9.639  1.00 15.00      A    C
ATOM    717  C   GLU A 182      -6.822   4.373  -8.906  1.00 15.00      A    C
ATOM    718  O   GLU A 182      -5.594   4.385  -8.890  1.00 15.00      A    O
ATOM    719  CB  GLU A 182      -7.994   3.756 -11.030  1.00 15.00      A    C
ATOM    720  CG  GLU A 182      -8.476   2.661 -11.973  1.00 15.00      A    C
ATOM    721  CD  GLU A 182      -9.892   2.200 -11.680  1.00 15.00      A    C
ATOM    722  OE1 GLU A 182     -10.168   0.992 -11.843  1.00 15.00      A    O
ATOM    723  OE2 GLU A 182     -10.724   3.047 -11.285  1.00 15.00      A    O1-
ATOM    724  HN  GLU A 182      -9.637   3.104  -9.183  1.00 15.00      A    H
ATOM    725  N   GLU A 183      -7.568   5.293  -8.294  1.00 15.00      A    N
ATOM    726  CA  GLU A 183      -6.972   6.401  -7.551  1.00 15.00      A    C
ATOM    727  C   GLU A 183      -6.395   5.928  -6.219  1.00 15.00      A    C
ATOM    728  O   GLU A 183      -5.487   6.550  -5.671  1.00 15.00      A    O
ATOM    729  CB  GLU A 183      -8.003   7.504  -7.309  1.00 15.00      A    C
ATOM    730  CG  GLU A 183      -8.596   8.089  -8.580  1.00 15.00      A    C
ATOM    731  CD  GLU A 183      -9.585   9.200  -8.301  1.00 15.00      A    C
ATOM    732  OE1 GLU A 183      -9.227  10.378  -8.513  1.00 15.00      A    O
ATOM    733  OE2 GLU A 183     -10.716   8.894  -7.867  1.00 15.00      A    O1-
ATOM    734  HN  GLU A 183      -8.545   5.227  -8.346  1.00 15.00      A    H
ATOM    735  N   LEU A 184      -6.942   4.829  -5.702  1.00 15.00      A    N
ATOM    736  CA  LEU A 184      -6.484   4.259  -4.439  1.00 15.00      A    C
ATOM    737  C   LEU A 184      -5.126   3.588  -4.613  1.00 15.00      A    C
ATOM    738  O   LEU A 184      -4.225   3.773  -3.799  1.00 15.00      A    O
ATOM    739  CB  LEU A 184      -7.510   3.254  -3.902  1.00 15.00      A    C
ATOM    740  CG  LEU A 184      -7.110   2.468  -2.648  1.00 15.00      A    C
ATOM    741  CD1 LEU A 184      -6.977   3.391  -1.446  1.00 15.00      A    C
ATOM    742  CD2 LEU A 184      -8.112   1.359  -2.369  1.00 15.00      A    C
ATOM    743  HN  LEU A 184      -7.676   4.391  -6.183  1.00 15.00      A    H
ATOM    744  N   THR A 185      -4.987   2.815  -5.689  1.00 15.00      A    N
ATOM    745  CA  THR A 185      -3.737   2.117  -5.978  1.00 15.00      A    C
ATOM    746  C   THR A 185      -2.622   3.089  -6.357  1.00 15.00      A    C
ATOM    747  O   THR A 185      -1.445   2.798  -6.153  1.00 15.00      A    O
ATOM    748  CB  THR A 185      -3.908   1.065  -7.097  1.00 15.00      A    C
ATOM    749  CG2 THR A 185      -4.836  -0.053  -6.646  1.00 15.00      A    C
ATOM    750  OG1 THR A 185      -4.442   1.685  -8.274  1.00 15.00      A    O
```

```
ATOM    751  HN  THR A 185      -5.743   2.711  -6.304  1.00 15.00      A    H
ATOM    752  HG1 THR A 185      -5.286   2.093  -8.067  1.00 15.00      A    H
ATOM    753  N   LEU A 186      -3.000   4.242  -6.912  1.00 15.00      A    N
ATOM    754  CA  LEU A 186      -2.028   5.259  -7.310  1.00 15.00      A    C
ATOM    755  C   LEU A 186      -1.506   6.026  -6.100  1.00 15.00      A    C
ATOM    756  O   LEU A 186      -0.324   6.355  -6.029  1.00 15.00      A    O
ATOM    757  CB  LEU A 186      -2.639   6.236  -8.318  1.00 15.00      A    C
ATOM    758  CG  LEU A 186      -2.867   5.701  -9.733  1.00 15.00      A    C
ATOM    759  CD1 LEU A 186      -3.637   6.711 -10.570  1.00 15.00      A    C
ATOM    760  CD2 LEU A 186      -1.544   5.350 -10.398  1.00 15.00      A    C
ATOM    761  HN  LEU A 186      -3.954   4.411  -7.060  1.00 15.00      A    H
ATOM    762  N   GLU A 187      -2.395   6.299  -5.146  1.00 15.00      A    N
ATOM    763  CA  GLU A 187      -2.029   7.031  -3.937  1.00 15.00      A    C
ATOM    764  C   GLU A 187      -1.160   6.175  -3.017  1.00 15.00      A    C
ATOM    765  O   GLU A 187      -0.245   6.683  -2.366  1.00 15.00      A    O
ATOM    766  CB  GLU A 187      -3.281   7.516  -3.200  1.00 15.00      A    C
ATOM    767  CG  GLU A 187      -3.020   8.589  -2.151  1.00 15.00      A    C
ATOM    768  CD  GLU A 187      -2.449   9.866  -2.740  1.00 15.00      A    C
ATOM    769  OE1 GLU A 187      -1.649  10.531  -2.050  1.00 15.00      A    O
ATOM    770  OE2 GLU A 187      -2.803  10.201  -3.890  1.00 15.00      A    O1-
ATOM    771  HN  GLU A 187      -3.320   5.999  -5.258  1.00 15.00      A    H
ATOM    772  N   VAL A 188      -1.454   4.875  -2.967  1.00 15.00      A    N
ATOM    773  CA  VAL A 188      -0.687   3.946  -2.140  1.00 15.00      A    C
ATOM    774  C   VAL A 188       0.701   3.722  -2.748  1.00 15.00      A    C
ATOM    775  O   VAL A 188       1.667   3.447  -2.037  1.00 15.00      A    O
ATOM    776  CB  VAL A 188      -1.421   2.591  -1.972  1.00 15.00      A    C
ATOM    777  CG1 VAL A 188      -0.551   1.574  -1.248  1.00 15.00      A    C
ATOM    778  CG2 VAL A 188      -2.731   2.785  -1.223  1.00 15.00      A    C
ATOM    779  HN  VAL A 188      -2.204   4.533  -3.498  1.00 15.00      A    H
ATOM    780  N   GLN A 189       0.792   3.866  -4.071  1.00 15.00      A    N
ATOM    781  CA  GLN A 189       2.053   3.689  -4.783  1.00 15.00      A    C
ATOM    782  C   GLN A 189       2.972   4.903  -4.599  1.00 15.00      A    C
ATOM    783  O   GLN A 189       4.197   4.768  -4.607  1.00 15.00      A    O
ATOM    784  CB  GLN A 189       1.786   3.431  -6.273  1.00 15.00      A    C
ATOM    785  CG  GLN A 189       3.016   3.044  -7.083  1.00 15.00      A    C
ATOM    786  CD  GLN A 189       3.753   4.245  -7.649  1.00 15.00      A    C
ATOM    787  NE2 GLN A 189       5.074   4.133  -7.751  1.00 15.00      A    N
ATOM    788  OE1 GLN A 189       3.144   5.262  -7.983  1.00 15.00      A    O
ATOM    789  HN  GLN A 189      -0.013   4.093  -4.581  1.00 15.00      A    H
ATOM    790 HE21 GLN A 189       5.492   3.295  -7.462  1.00 15.00      A    H
ATOM    791 HE22 GLN A 189       5.578   4.895  -8.109  1.00 15.00      A    H
ATOM    792  N   LYS A 190       2.370   6.080  -4.424  1.00 15.00      A    N
ATOM    793  CA  LYS A 190       3.126   7.325  -4.248  1.00 15.00      A    C
ATOM    794  C   LYS A 190       3.913   7.358  -2.936  1.00 15.00      A    C
ATOM    795  O   LYS A 190       4.889   8.098  -2.814  1.00 15.00      A    O
ATOM    796  CB  LYS A 190       2.195   8.541  -4.310  1.00 15.00      A    C
ATOM    797  CG  LYS A 190       1.547   8.785  -5.661  1.00 15.00      A    C
```

```
ATOM    798  CD  LYS A 190       0.482   9.867  -5.563  1.00 15.00      A    C
ATOM    799  CE  LYS A 190      -0.350   9.953  -6.832  1.00 15.00      A    C
ATOM    800  NZ  LYS A 190      -1.475  10.920  -6.691  1.00 15.00      A    N1+
ATOM    801  HN  LYS A 190       1.392   6.115  -4.417  1.00 15.00      A    H
ATOM    802  HZ1 LYS A 190      -1.105  11.876  -6.514  1.00 15.00      A    H
ATOM    803  HZ2 LYS A 190      -2.086  10.645  -5.894  1.00 15.00      A    H
ATOM    804  HZ3 LYS A 190      -2.045  10.935  -7.560  1.00 15.00      A    H
ATOM    805  N   SER A 191       3.490   6.555  -1.958  1.00 15.00      A    N
ATOM    806  CA  SER A 191       4.156   6.517  -0.653  1.00 15.00      A    C
ATOM    807  C   SER A 191       5.492   5.768  -0.701  1.00 15.00      A    C
ATOM    808  O   SER A 191       6.242   5.765   0.276  1.00 15.00      A    O
ATOM    809  CB  SER A 191       3.237   5.914   0.415  1.00 15.00      A    C
ATOM    810  OG  SER A 191       3.041   4.526   0.207  1.00 15.00      A    O
ATOM    811  HN  SER A 191       2.718   5.972  -2.116  1.00 15.00      A    H
ATOM    812  HG  SER A 191       2.160   4.279   0.497  1.00 15.00      A    H
ATOM    813  N   PHE A 192       5.782   5.142  -1.838  1.00 15.00      A    N
ATOM    814  CA  PHE A 192       7.029   4.396  -2.013  1.00 15.00      A    C
ATOM    815  C   PHE A 192       8.076   5.231  -2.748  1.00 15.00      A    C
ATOM    816  O   PHE A 192       9.263   4.903  -2.738  1.00 15.00      A    O
ATOM    817  CB  PHE A 192       6.772   3.114  -2.812  1.00 15.00      A    C
ATOM    818  CG  PHE A 192       5.993   2.062  -2.078  1.00 15.00      A    C
ATOM    819  CD1 PHE A 192       6.647   1.064  -1.378  1.00 15.00      A    C
ATOM    820  CD2 PHE A 192       4.607   2.064  -2.098  1.00 15.00      A    C
ATOM    821  CE1 PHE A 192       5.936   0.086  -0.709  1.00 15.00      A    C
ATOM    822  CE2 PHE A 192       3.888   1.090  -1.430  1.00 15.00      A    C
ATOM    823  CZ  PHE A 192       4.554   0.099  -0.735  1.00 15.00      A    C
ATOM    824  HN  PHE A 192       5.142   5.181  -2.580  1.00 15.00      A    H
ATOM    825  N   VAL A 193       7.631   6.314  -3.377  1.00 15.00      A    N
ATOM    826  CA  VAL A 193       8.523   7.182  -4.144  1.00 15.00      A    C
ATOM    827  C   VAL A 193       9.150   8.280  -3.282  1.00 15.00      A    C
ATOM    828  O   VAL A 193      10.238   8.773  -3.587  1.00 15.00      A    O
ATOM    829  CB  VAL A 193       7.777   7.831  -5.333  1.00 15.00      A    C
ATOM    830  CG1 VAL A 193       8.756   8.458  -6.315  1.00 15.00      A    C
ATOM    831  CG2 VAL A 193       6.891   6.814  -6.038  1.00 15.00      A    C
ATOM    832  HN  VAL A 193       6.680   6.538  -3.323  1.00 15.00      A    H
ATOM    833  N   SER A 194       8.474   8.648  -2.197  1.00 15.00      A    N
ATOM    834  CA  SER A 194       8.959   9.704  -1.309  1.00 15.00      A    C
ATOM    835  C   SER A 194      10.021   9.216  -0.318  1.00 15.00      A    C
ATOM    836  O   SER A 194      10.377   9.937   0.615  1.00 15.00      A    O
ATOM    837  CB  SER A 194       7.791  10.337  -0.552  1.00 15.00      A    C
ATOM    838  OG  SER A 194       6.788  10.786  -1.446  1.00 15.00      A    O
ATOM    839  HN  SER A 194       7.627   8.203  -1.988  1.00 15.00      A    H
ATOM    840  HG  SER A 194       7.200  11.197  -2.209  1.00 15.00      A    H
TER
ATOM    841  CAK UNK B   1      15.819  -2.001   2.889  1.00 15.00      B    C
ATOM    842  CAL UNK B   1      16.479   0.012   2.061  1.00 15.00      B    C
ATOM    843  CAM UNK B   1      16.983  -2.168   2.149  1.00 15.00      B    C
```

```
ATOM    844  CAN UNK B   1      16.888   1.369   2.637  1.00 15.00       B    C
ATOM    845  CAO UNK B   1      14.030   1.622   3.249  1.00 15.00       B    C
ATOM    846  CAP UNK B   1      17.587   1.426   3.839  1.00 15.00       B    C
ATOM    847  CAQ UNK B   1      17.751  -0.458  -1.409  1.00 15.00       B    C
ATOM    848  CAR UNK B   1      17.497  -3.400   1.760  1.00 15.00       B    C
ATOM    849  CAS UNK B   1      18.617  -3.457   0.939  1.00 15.00       B    C
ATOM    850  CAT UNK B   1      18.740  -0.167  -2.341  1.00 15.00       B    C
ATOM    851  CAU UNK B   1      16.582   2.542   1.957  1.00 15.00       B    C
ATOM    852  CAV UNK B   1      17.164  -1.718  -1.387  1.00 15.00       B    C
ATOM    853  CAW UNK B   1      16.885  -4.574   2.184  1.00 15.00       B    C
ATOM    854  CAX UNK B   1      17.676   3.827   3.676  1.00 15.00       B    C
ATOM    855  CAY UNK B   1      16.976   3.771   2.476  1.00 15.00       B    C
ATOM    856  CAZ UNK B   1      14.343   2.574   4.212  1.00 15.00       B    C
ATOM    857  NAH UNK B   1      15.486  -0.715   2.881  1.00 15.00       B    N
ATOM    858  NAI UNK B   1      17.446  -1.166   1.899  1.00 15.00       B    N
ATOM    859  NAJ UNK B   1      16.041   0.166   0.666  1.00 15.00       B    N
ATOM    860  OAC UNK B   1      15.202  -2.896   3.463  1.00 15.00       B    O
ATOM    861  OAD UNK B   1      16.760   1.972  -0.996  1.00 15.00       B    O
ATOM    862  OAE UNK B   1      12.948  -0.797   3.138  1.00 15.00       B    O
ATOM    863  OAF UNK B   1      18.413   1.140   0.603  1.00 15.00       B    O
ATOM    864  OAG UNK B   1      14.304  -0.260   5.101  1.00 15.00       B    O
ATOM    865  SAA UNK B   1      17.246   0.760  -0.259  1.00 15.00       B    S
ATOM    866  SAB UNK B   1      14.176  -0.082   3.616  1.00 15.00       B    S
ATOM    867  CBA UNK B   1      17.980   2.654   4.359  1.00 15.00       B    C
ATOM    868  CBB UNK B   1      18.513  -5.863   0.964  1.00 15.00       B    C
ATOM    869  CBC UNK B   1      13.583   2.023   1.996  1.00 15.00       B    C
ATOM    870  CBD UNK B   1      19.125  -4.688   0.541  1.00 15.00       B    C
ATOM    871  CBE UNK B   1      17.393  -5.806   1.785  1.00 15.00       B    C
ATOM    872  CBF UNK B   1      18.555  -2.395  -3.232  1.00 15.00       B    C
ATOM    873  CBG UNK B   1      19.143  -1.136  -3.253  1.00 15.00       B    C
ATOM    874  CBH UNK B   1      13.449   3.375   1.703  1.00 15.00       B    C
ATOM    875  CBI UNK B   1      14.210   3.927   3.920  1.00 15.00       B    C
ATOM    876  CBJ UNK B   1      13.766   4.327   2.665  1.00 15.00       B    C
ATOM    877  CBK UNK B   1      17.566  -2.688  -2.300  1.00 15.00       B    C
ATOM    878  HAA UNK B   1      17.812  -0.980   0.877  1.00 15.00       B    H
ATOM    879  HAB UNK B   1      15.253   0.779   0.620  1.00 15.00       B    H
END
```

PROCHECK

Ramachandran Plot
yog2

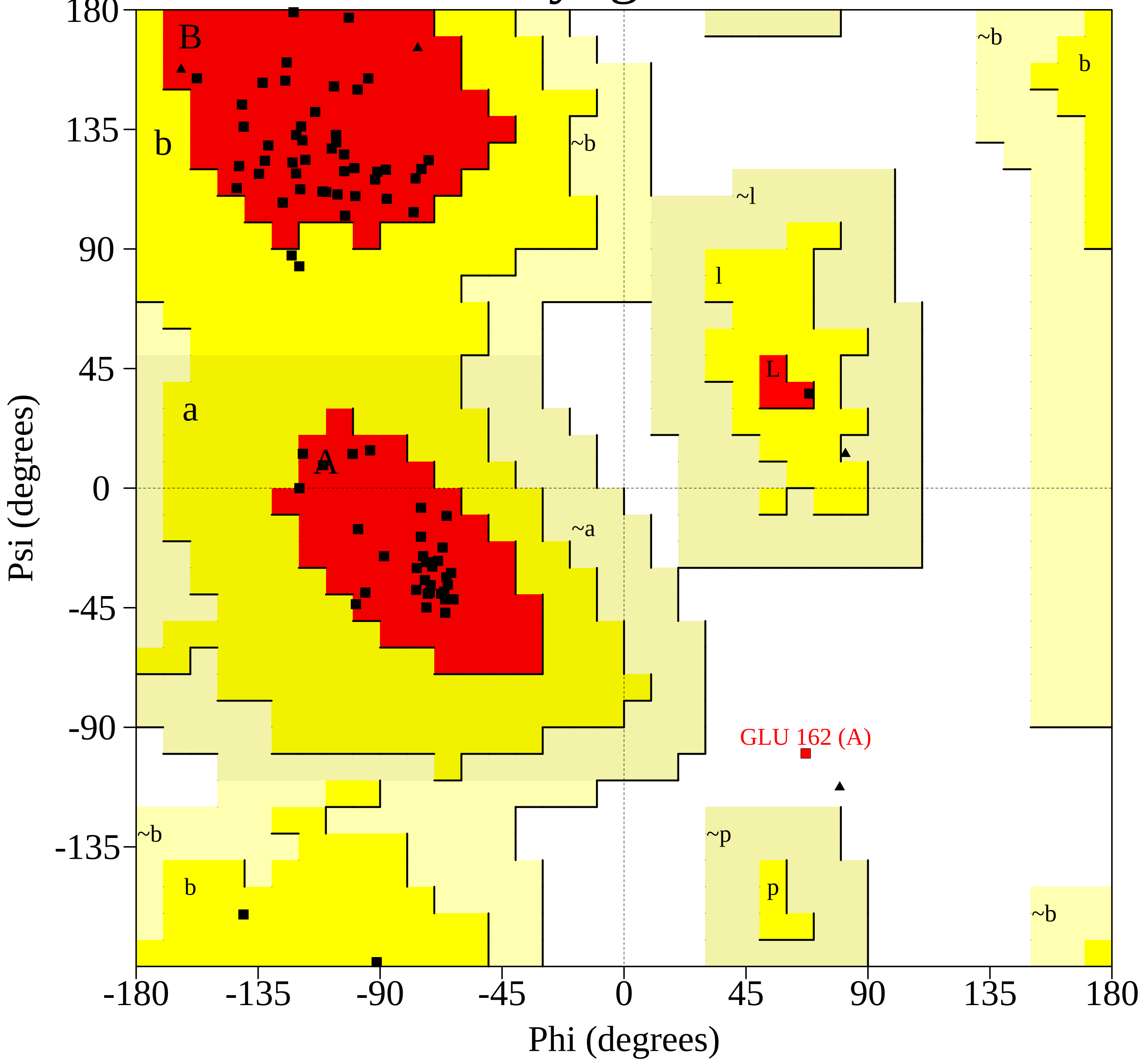

Plot statistics

Residues in most favoured regions [A,B,L]	77	93.9%
Residues in additional allowed regions [a,b,l,p]	4	4.9%
Residues in generously allowed regions [~a,~b,~l,~p]	0	0.0%
Residues in disallowed regions	1	1.2%
	----	------
Number of non-glycine and non-proline residues	82	100.0%
Number of end-residues (excl. Gly and Pro)	3	
Number of glycine residues (shown as triangles)	4	
Number of proline residues	1	

Total number of residues	90	

Based on an analysis of 118 structures of resolution of at least 2.0 Angstroms and R-factor no greater than 20%, a good quality model would be expected to have over 90% in the most favoured regions.

yog2_01.ps

Ramachandran plots for all residue types

yog2

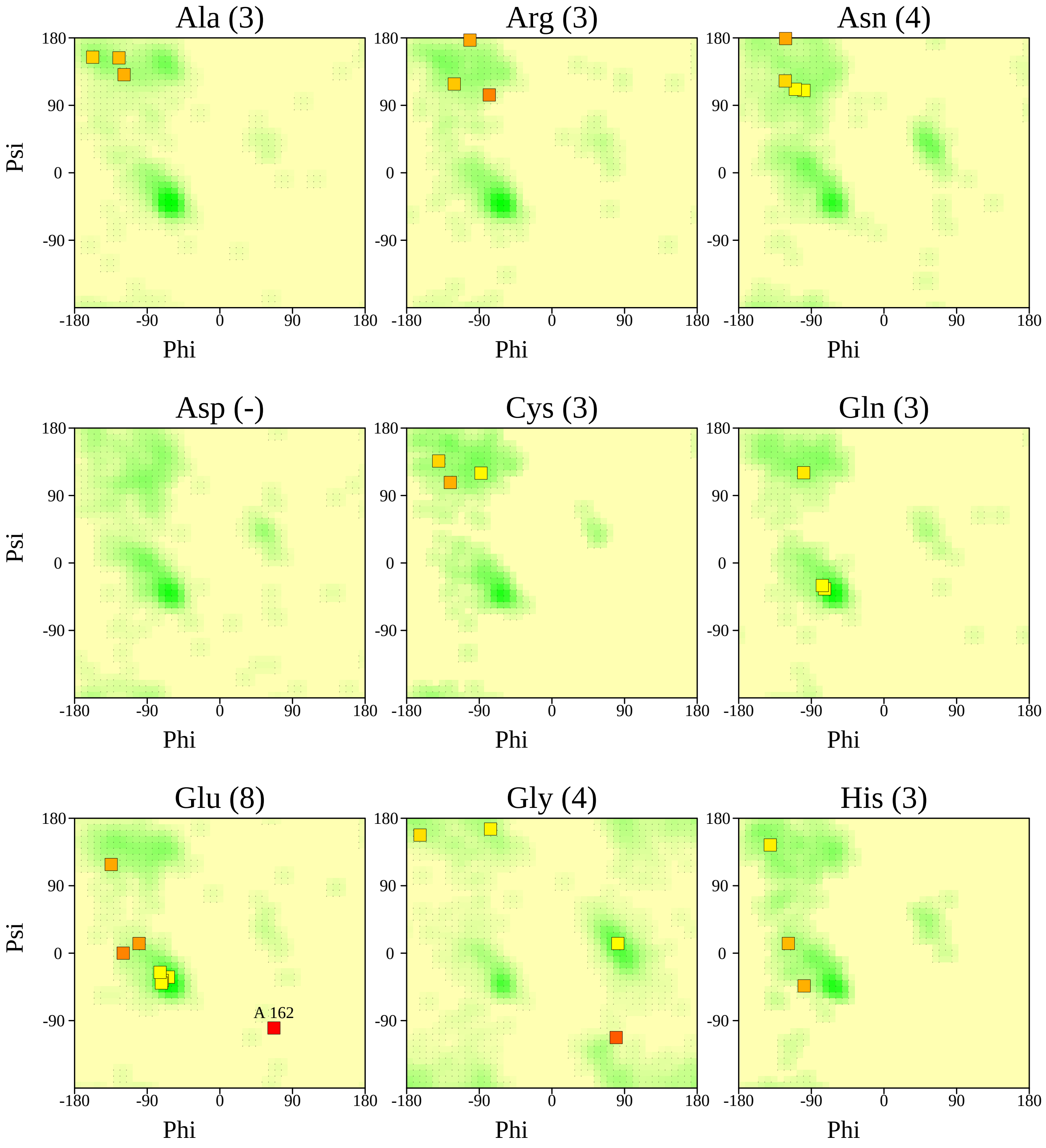

Numbers of residues are shown in brackets. Those in unfavourable conformations (score < -3.00) are labelled.
Shading shows favourable conformations as obtained from an analysis of 163 structures at resolution 2.0A or better.

Ramachandran plots for all residue types

yog2

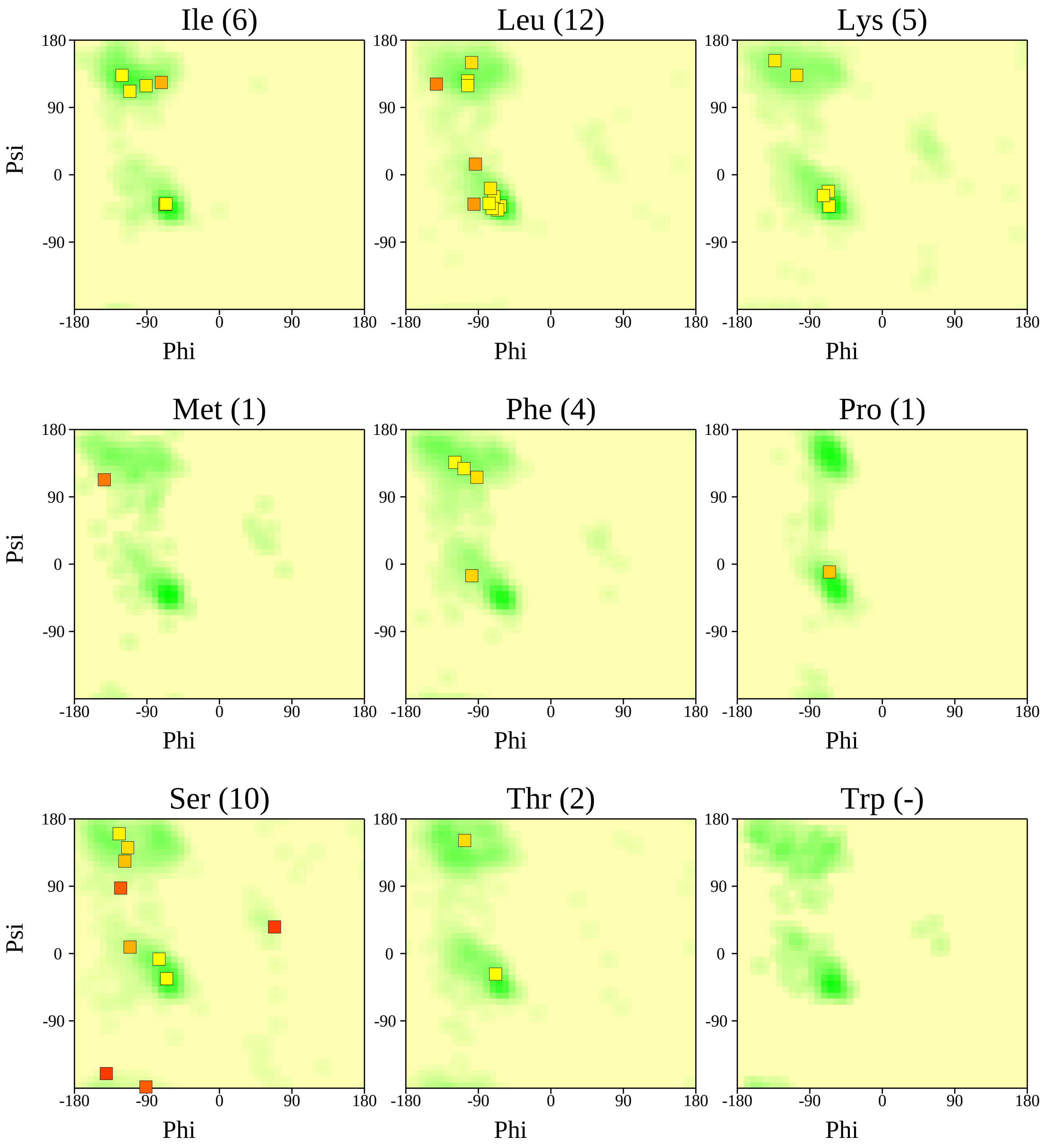

Numbers of residues are shown in brackets. Those in unfavourable conformations (score < -3.00) are labelled.
Shading shows favourable conformations as obtained from an analysis of 163 structures at resolution 2.0A or better.

Ramachandran plots for all residue types

yog2

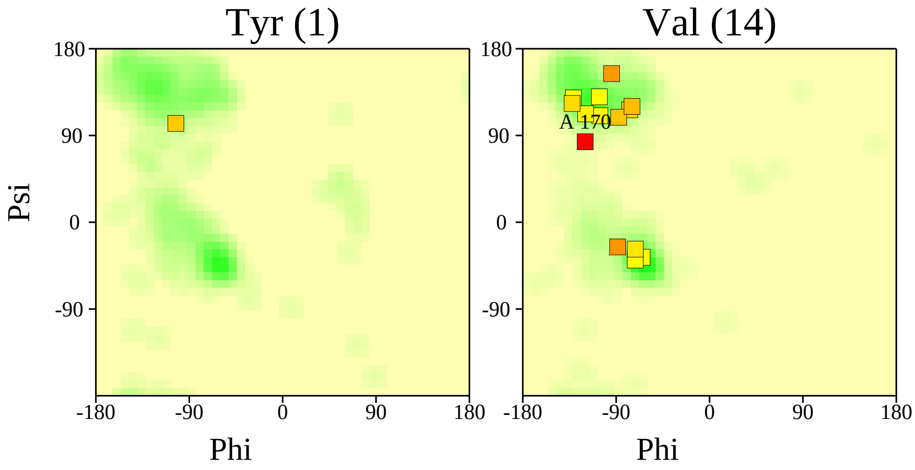

Numbers of residues are shown in brackets. Those in unfavourable conformations (score < -3.00) are labelled.
Shading shows favourable conformations as obtained from an analysis of 163 structures at resolution 2.0A or better.

Chi1-Chi2 plots

yog2

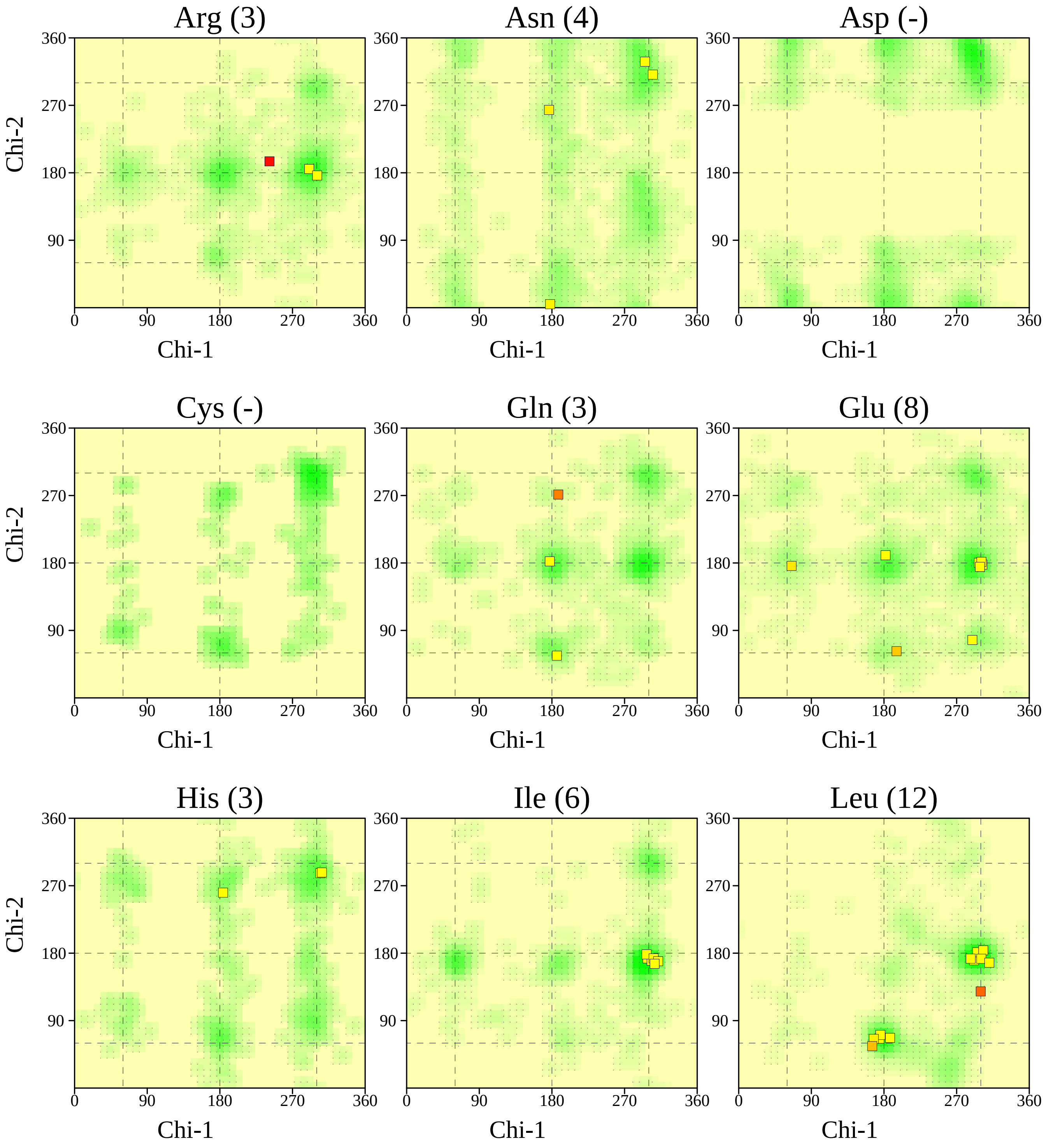

Numbers of residues are shown in brackets. Those in unfavourable conformations (score < -3.00) are labelled.
Shading shows favourable conformations as obtained from an analysis of 163 structures at resolution 2.0A or better.

Chi1-Chi2 plots

yog2

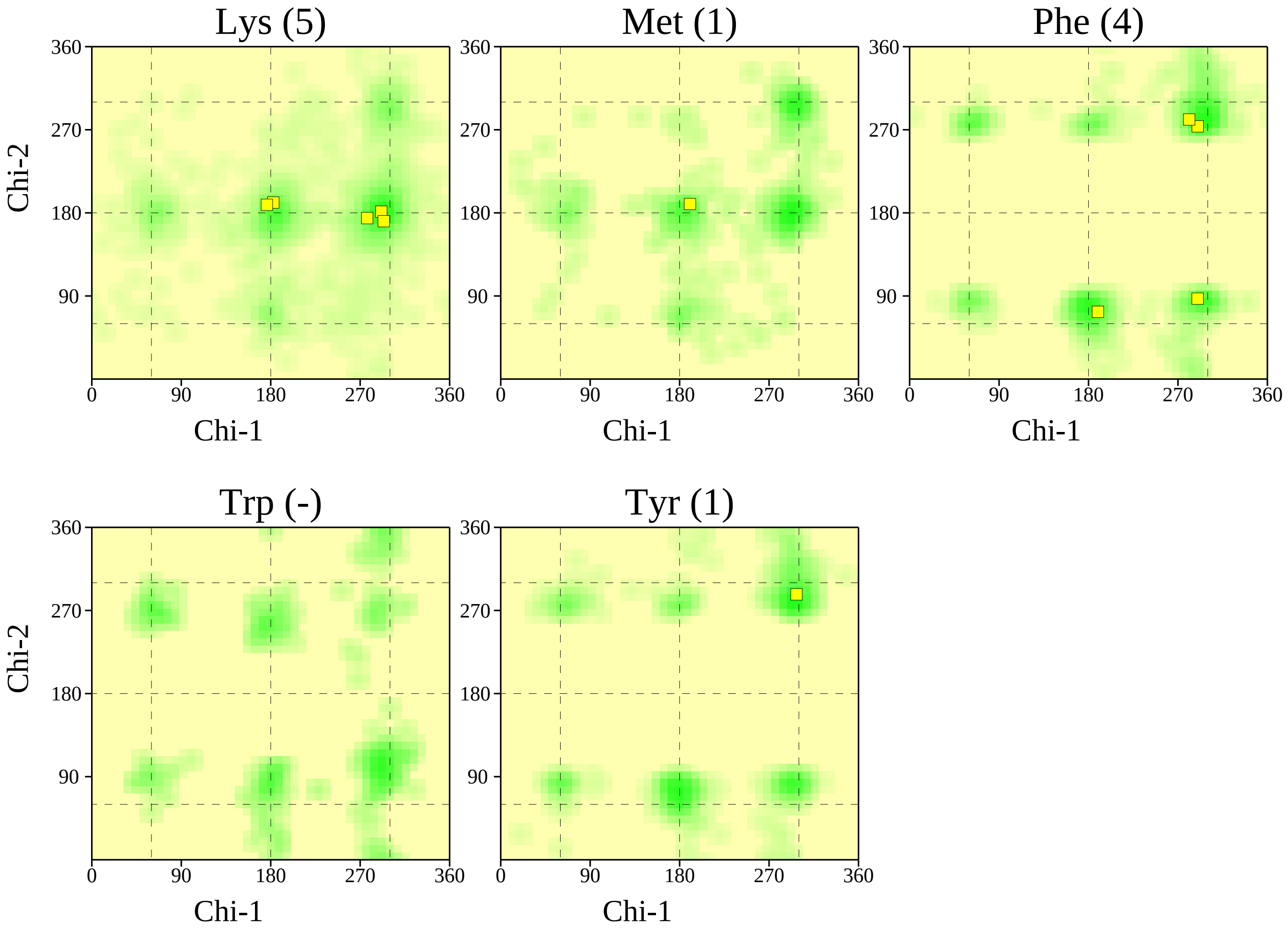

Numbers of residues are shown in brackets. Those in unfavourable conformations (score < -3.00) are labelled.
Shading shows favourable conformations as obtained from an analysis of 163 structures at resolution 2.0A or better.

PROCHECK

Main-chain parameters
yog2

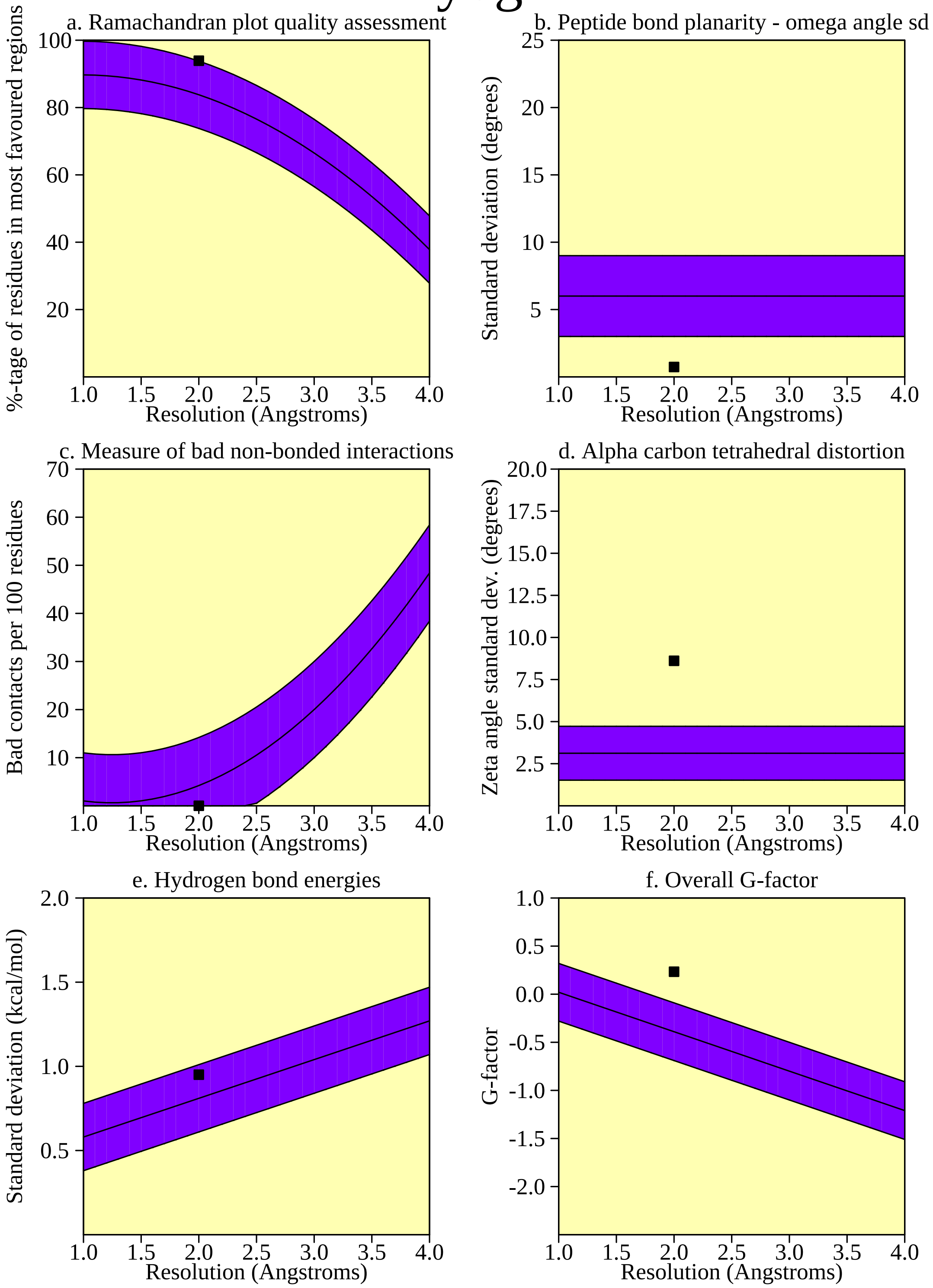

Plot statistics

Stereochemical parameter	No. of data pts	Parameter value	Comparison values: Typical value	Comparison values: Band width	No. of band widths from mean	
a. %-tage residues in A, B, L	82	93.9	83.8	10.0	1.0	BETTER
b. Omega angle st dev	88	0.7	6.0	3.0	-1.8	BETTER
c. Bad contacts / 100 residues	0	0.0	4.2	10.0	-0.4	Inside
d. Zeta angle st dev	85	8.6	3.1	1.6	3.4	WORSE
e. H-bond energy st dev	66	1.0	0.8	0.2	0.7	Inside
f. Overall G-factor	90	0.2	-0.4	0.3	2.1	BETTER

yog2_04.ps

PROCHECK

Side-chain parameters

yog2

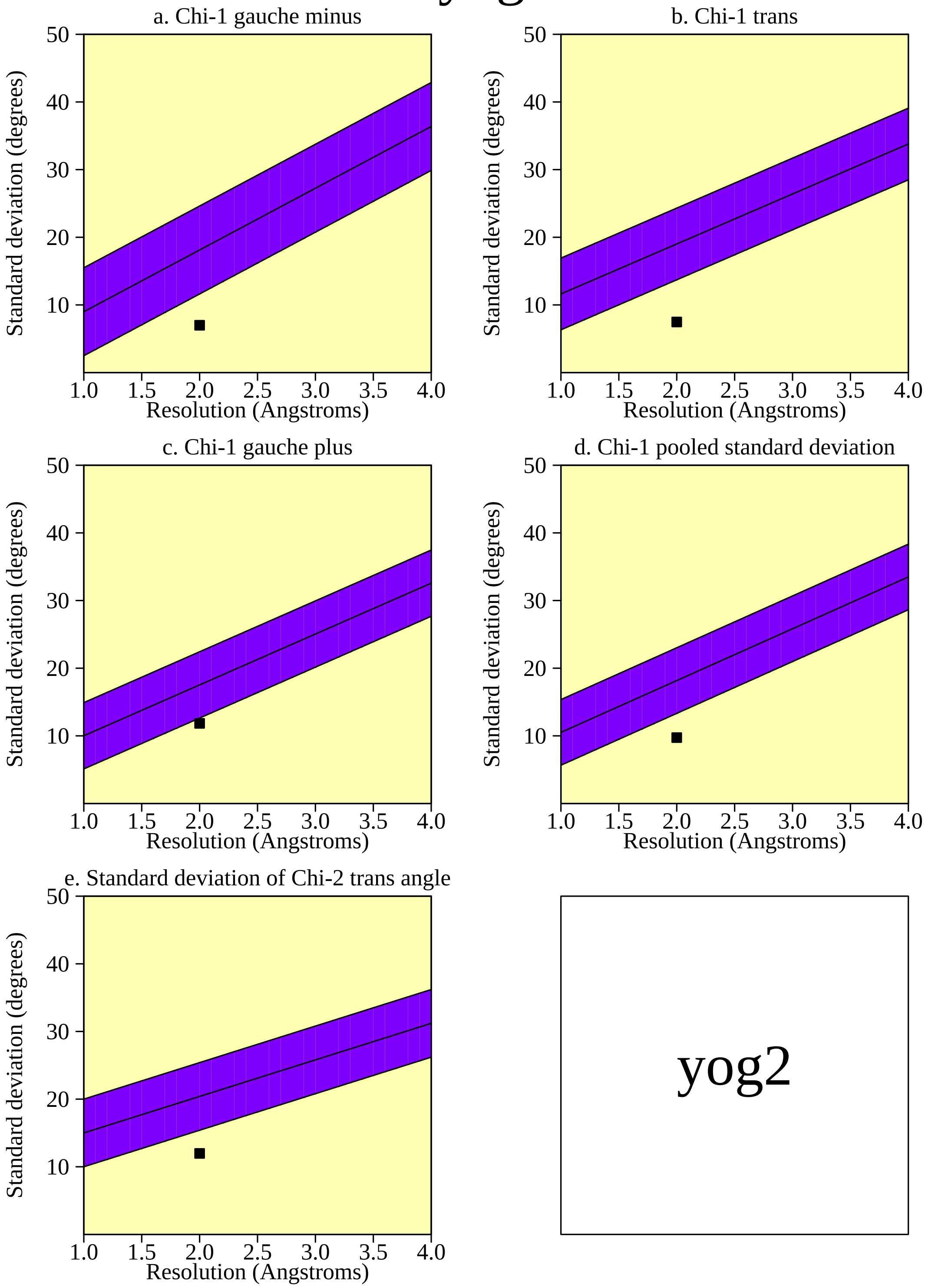

Plot statistics

Stereochemical parameter	No. of data pts	Parameter value	Comparison values: Typical value	Comparison values: Band width	No. of band widths from mean	
a. Chi-1 gauche minus st dev	9	7.0	18.1	6.5	-1.7	BETTER
b. Chi-1 trans st dev	34	7.5	19.0	5.3	-2.2	BETTER
c. Chi-1 gauche plus st dev	38	11.8	17.5	4.9	-1.2	BETTER
d. Chi-1 pooled st dev	81	9.7	18.2	4.8	-1.7	BETTER
e. Chi-2 trans st dev	29	12.0	20.4	5.0	-1.7	BETTER

Main-chain bond lengths

yog2

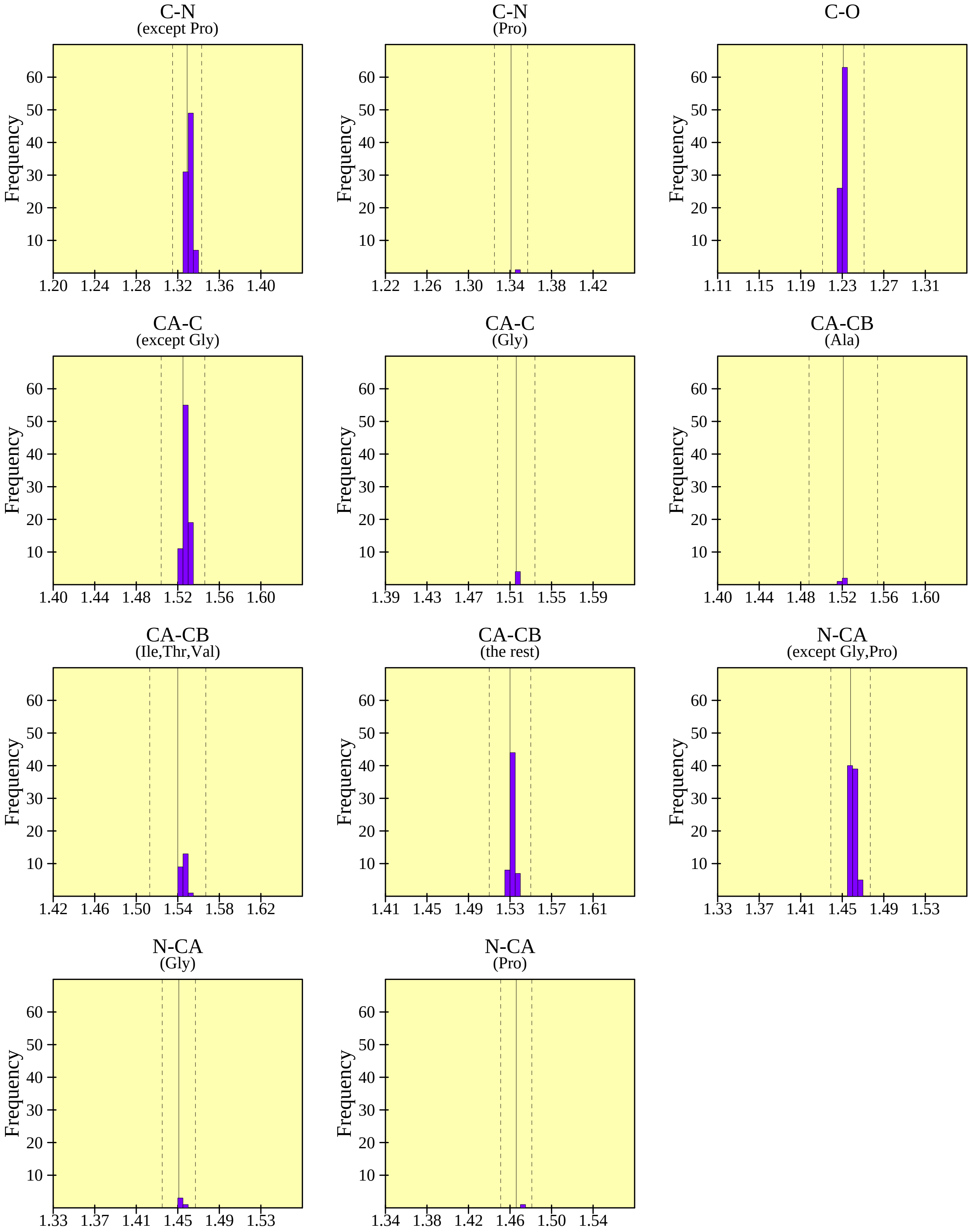

Black bars > 2.0 st. devs. from mean.

Solid and dashed lines represent the mean and standard deviation values as per Engh & Huber small-molecule data.

MUTE_ACT_Stomidazolone (b form)

ATOM	1	N	VAL	A	106	14.501	5.411	-11.175	1.00	15.00	A	N
ATOM	2	HN	VAL	A	106	15.461	5.604	-11.143	1.00	15.00	A	H
ATOM	3	CA	VAL	A	106	14.048	4.029	-11.054	1.00	15.00	A	C
ATOM	4	CB	VAL	A	106	15.166	3.111	-10.513	1.00	15.00	A	C
ATOM	5	CG1	VAL	A	106	14.720	1.656	-10.521	1.00	15.00	A	C
ATOM	6	CG2	VAL	A	106	16.443	3.284	-11.322	1.00	15.00	A	C
ATOM	7	C	VAL	A	106	12.827	3.934	-10.139	1.00	15.00	A	C
ATOM	8	O	VAL	A	106	12.924	4.155	-8.930	1.00	15.00	A	O
ATOM	9	N	GLY	A	107	11.679	3.602	-10.725	1.00	15.00	A	N
ATOM	10	HN	GLY	A	107	11.666	3.428	-11.689	1.00	15.00	A	H
ATOM	11	CA	GLY	A	107	10.455	3.486	-9.956	1.00	15.00	A	C
ATOM	12	C	GLY	A	107	9.387	2.690	-10.680	1.00	15.00	A	C
ATOM	13	O	GLY	A	107	9.416	2.571	-11.905	1.00	15.00	A	O
ATOM	14	N	ALA	A	108	8.440	2.149	-9.920	1.00	15.00	A	N
ATOM	15	HN	ALA	A	108	8.472	2.287	-8.950	1.00	15.00	A	H
ATOM	16	CA	ALA	A	108	7.356	1.357	-10.488	1.00	15.00	A	C
ATOM	17	CB	ALA	A	108	7.463	-0.088	-10.031	1.00	15.00	A	C
ATOM	18	C	ALA	A	108	5.999	1.932	-10.100	1.00	15.00	A	C
ATOM	19	O	ALA	A	108	5.852	2.534	-9.036	1.00	15.00	A	O
ATOM	20	N	CYS	A	109	5.011	1.742	-10.968	1.00	15.00	A	N
ATOM	21	HN	CYS	A	109	5.192	1.257	-11.800	1.00	15.00	A	H
ATOM	22	CA	CYS	A	109	3.665	2.240	-10.713	1.00	15.00	A	C
ATOM	23	CB	CYS	A	109	3.431	3.577	-11.424	1.00	15.00	A	C
ATOM	24	SG	CYS	A	109	3.658	3.526	-13.218	1.00	15.00	A	S
ATOM	25	HG	CYS	A	109	4.957	3.589	-13.467	1.00	15.00	A	H
ATOM	26	C	CYS	A	109	2.602	1.221	-11.121	1.00	15.00	A	C
ATOM	27	O	CYS	A	109	2.688	0.607	-12.184	1.00	15.00	A	O
ATOM	28	N	CYS	A	110	1.605	1.048	-10.259	1.00	15.00	A	N
ATOM	29	HN	CYS	A	110	1.605	1.562	-9.425	1.00	15.00	A	H
ATOM	30	CA	CYS	A	110	0.514	0.119	-10.515	1.00	15.00	A	C
ATOM	31	CB	CYS	A	110	0.585	-1.075	-9.562	1.00	15.00	A	C
ATOM	32	SG	CYS	A	110	-0.697	-2.317	-9.840	1.00	15.00	A	S
ATOM	33	HG	CYS	A	110	-1.842	-1.681	-10.037	1.00	15.00	A	H
ATOM	34	C	CYS	A	110	-0.827	0.830	-10.366	1.00	15.00	A	C
ATOM	35	O	CYS	A	110	-1.238	1.176	-9.259	1.00	15.00	A	O
ATOM	36	N	ASN	A	111	-1.499	1.050	-11.490	1.00	15.00	A	N
ATOM	37	HN	ASN	A	111	-1.122	0.742	-12.340	1.00	15.00	A	H
ATOM	38	CA	ASN	A	111	-2.786	1.734	-11.490	1.00	15.00	A	C
ATOM	39	CB	ASN	A	111	-2.850	2.746	-12.637	1.00	15.00	A	C
ATOM	40	CG	ASN	A	111	-1.824	3.856	-12.499	1.00	15.00	A	C
ATOM	41	OD1	ASN	A	111	-1.484	4.267	-11.392	1.00	15.00	A	O
ATOM	42	ND2	ASN	A	111	-1.325	4.347	-13.628	1.00	15.00	A	N
ATOM	43	HD21	ASN	A	111	-1.641	3.971	-14.475	1.00	15.00	A	H
ATOM	44	HD22	ASN	A	111	-0.660	5.065	-13.567	1.00	15.00	A	H
ATOM	45	C	ASN	A	111	-3.948	0.752	-11.595	1.00	15.00	A	C

```
ATOM     46  O   ASN A 111      -4.148   0.117 -12.632  1.00 15.00      A    O
ATOM     47  N   SER A 112      -4.709   0.632 -10.512  1.00 15.00      A    N
ATOM     48  HN  SER A 112      -4.489   1.158  -9.714  1.00 15.00      A    H
ATOM     49  CA  SER A 112      -5.861  -0.261 -10.476  1.00 15.00      A    C
ATOM     50  CB  SER A 112      -5.619  -1.401  -9.479  1.00 15.00      A    C
ATOM     51  OG  SER A 112      -5.804  -0.965  -8.145  1.00 15.00      A    O
ATOM     52  HG  SER A 112      -6.257  -0.118  -8.146  1.00 15.00      A    H
ATOM     53  C   SER A 112      -7.120   0.521 -10.096  1.00 15.00      A    C
ATOM     54  O   SER A 112      -7.023   1.632  -9.570  1.00 15.00      A    O
ATOM     55  N   PRO A 113      -8.320  -0.040 -10.361  1.00 15.00      A    N
ATOM     56  CA  PRO A 113      -9.596   0.619 -10.036  1.00 15.00      A    C
ATOM     57  CB  PRO A 113     -10.657  -0.342 -10.593  1.00 15.00      A    C
ATOM     58  CG  PRO A 113      -9.925  -1.212 -11.557  1.00 15.00      A    C
ATOM     59  CD  PRO A 113      -8.532  -1.342 -11.016  1.00 15.00      A    C
ATOM     60  C   PRO A 113      -9.804   0.806  -8.533  1.00 15.00      A    C
ATOM     61  O   PRO A 113     -10.715   1.517  -8.111  1.00 15.00      A    O
ATOM     62  N   HIS A 114      -8.954   0.169  -7.730  1.00 15.00      A    N
ATOM     63  HN  HIS A 114      -8.244  -0.380  -8.123  1.00 15.00      A    H
ATOM     64  CA  HIS A 114      -9.058   0.267  -6.279  1.00 15.00      A    C
ATOM     65  CB  HIS A 114      -8.933  -1.112  -5.623  1.00 15.00      A    C
ATOM     66  CG  HIS A 114     -10.003  -2.078  -6.031  1.00 15.00      A    C
ATOM     67  ND1 HIS A 114     -11.335  -1.789  -5.854  1.00 15.00      A    N
ATOM     68  CD2 HIS A 114      -9.884  -3.300  -6.608  1.00 15.00      A    C
ATOM     69  CE1 HIS A 114     -11.996  -2.831  -6.323  1.00 15.00      A    C
ATOM     70  NE2 HIS A 114     -11.160  -3.769  -6.789  1.00 15.00      A    N
ATOM     71  HE2 HIS A 114     -11.412  -4.631  -7.182  1.00 15.00      A    H
ATOM     72  C   HIS A 114      -8.030   1.231  -5.688  1.00 15.00      A    C
ATOM     73  O   HIS A 114      -8.385   2.115  -4.907  1.00 15.00      A    O
ATOM     74  N   ALA A 115      -6.758   1.067  -6.061  1.00 15.00      A    N
ATOM     75  HN  ALA A 115      -6.529   0.359  -6.697  1.00 15.00      A    H
ATOM     76  CA  ALA A 115      -5.704   1.930  -5.529  1.00 15.00      A    C
ATOM     77  CB  ALA A 115      -5.191   1.378  -4.209  1.00 15.00      A    C
ATOM     78  C   ALA A 115      -4.535   2.133  -6.492  1.00 15.00      A    C
ATOM     79  O   ALA A 115      -4.248   1.288  -7.339  1.00 15.00      A    O
ATOM     80  N   ASN A 116      -3.863   3.272  -6.331  1.00 15.00      A    N
ATOM     81  HN  ASN A 116      -4.166   3.904  -5.644  1.00 15.00      A    H
ATOM     82  CA  ASN A 116      -2.698   3.625  -7.137  1.00 15.00      A    C
ATOM     83  CB  ASN A 116      -2.863   5.050  -7.688  1.00 15.00      A    C
ATOM     84  CG  ASN A 116      -1.676   5.548  -8.499  1.00 15.00      A    C
ATOM     85  OD1 ASN A 116      -0.721   4.814  -8.756  1.00 15.00      A    O
ATOM     86  ND2 ASN A 116      -1.737   6.808  -8.919  1.00 15.00      A    N
ATOM     87 HD21 ASN A 116      -2.532   7.335  -8.687  1.00 15.00      A    H
ATOM     88 HD22 ASN A 116      -0.986   7.161  -9.444  1.00 15.00      A    H
ATOM     89  C   ASN A 116      -1.438   3.528  -6.272  1.00 15.00      A    C
ATOM     90  O   ASN A 116      -1.249   4.309  -5.340  1.00 15.00      A    O
ATOM     91  N   VAL A 117      -0.583   2.559  -6.584  1.00 15.00      A    N
ATOM     92  HN  VAL A 117      -0.782   1.973  -7.343  1.00 15.00      A    H
```

```
ATOM     93  CA  VAL A 117       0.647   2.347  -5.824  1.00 15.00      A    C
ATOM     94  CB  VAL A 117       0.800   0.869  -5.404  1.00 15.00      A    C
ATOM     95  CG1 VAL A 117       1.900   0.716  -4.366  1.00 15.00      A    C
ATOM     96  CG2 VAL A 117      -0.515   0.317  -4.870  1.00 15.00      A    C
ATOM     97  C   VAL A 117       1.883   2.782  -6.615  1.00 15.00      A    C
ATOM     98  O   VAL A 117       2.084   2.361  -7.751  1.00 15.00      A    O
ATOM     99  N   GLU A 118       2.711   3.622  -5.997  1.00 15.00      A    N
ATOM    100  HN  GLU A 118       2.492   3.922  -5.090  1.00 15.00      A    H
ATOM    101  CA  GLU A 118       3.930   4.115  -6.634  1.00 15.00      A    C
ATOM    102  CB  GLU A 118       3.750   5.583  -7.034  1.00 15.00      A    C
ATOM    103  CG  GLU A 118       4.946   6.196  -7.746  1.00 15.00      A    C
ATOM    104  CD  GLU A 118       4.816   7.697  -7.927  1.00 15.00      A    C
ATOM    105  OE1 GLU A 118       5.821   8.339  -8.300  1.00 15.00      A    O
ATOM    106  OE2 GLU A 118       3.712   8.234  -7.692  1.00 15.00      A    O
ATOM    107  C   GLU A 118       5.133   3.974  -5.699  1.00 15.00      A    C
ATOM    108  O   GLU A 118       5.143   4.531  -4.603  1.00 15.00      A    O
ATOM    109  N   ALA A 119       6.146   3.232  -6.140  1.00 15.00      A    N
ATOM    110  HN  ALA A 119       6.087   2.819  -7.027  1.00 15.00      A    H
ATOM    111  CA  ALA A 119       7.347   3.023  -5.334  1.00 15.00      A    C
ATOM    112  CB  ALA A 119       7.495   1.554  -4.968  1.00 15.00      A    C
ATOM    113  C   ALA A 119       8.599   3.524  -6.049  1.00 15.00      A    C
ATOM    114  O   ALA A 119       8.847   3.176  -7.202  1.00 15.00      A    O
ATOM    115  N   LYS A 120       9.386   4.341  -5.354  1.00 15.00      A    N
ATOM    116  HN  LYS A 120       9.134   4.578  -4.437  1.00 15.00      A    H
ATOM    117  CA  LYS A 120      10.617   4.893  -5.917  1.00 15.00      A    C
ATOM    118  CB  LYS A 120      10.425   6.363  -6.299  1.00 15.00      A    C
ATOM    119  CG  LYS A 120       9.717   6.575  -7.627  1.00 15.00      A    C
ATOM    120  CD  LYS A 120       9.444   8.049  -7.880  1.00 15.00      A    C
ATOM    121  CE  LYS A 120       8.902   8.273  -9.282  1.00 15.00      A    C
ATOM    122  NZ  LYS A 120       8.429   9.671  -9.479  1.00 15.00      A    N
ATOM    123  HZ1 LYS A 120       8.168   9.825 -10.474  1.00 15.00      A    H
ATOM    124  HZ2 LYS A 120       9.180  10.343  -9.221  1.00 15.00      A    H
ATOM    125  HZ3 LYS A 120       7.597   9.853  -8.881  1.00 15.00      A    H
ATOM    126  C   LYS A 120      11.777   4.758  -4.938  1.00 15.00      A    C
ATOM    127  O   LYS A 120      11.577   4.739  -3.726  1.00 15.00      A    O
ATOM    128  N   ILE A 121      12.991   4.666  -5.471  1.00 15.00      A    N
ATOM    129  HN  ILE A 121      13.089   4.685  -6.446  1.00 15.00      A    H
ATOM    130  CA  ILE A 121      14.182   4.538  -4.638  1.00 15.00      A    C
ATOM    131  CB  ILE A 121      15.266   3.663  -5.307  1.00 15.00      A    C
ATOM    132  CG1 ILE A 121      14.743   2.239  -5.517  1.00 15.00      A    C
ATOM    133  CG2 ILE A 121      16.541   3.645  -4.470  1.00 15.00      A    C
ATOM    134  CD1 ILE A 121      15.702   1.333  -6.260  1.00 15.00      A    C
ATOM    135  C   ILE A 121      14.770   5.906  -4.299  1.00 15.00      A    C
ATOM    136  O   ILE A 121      15.130   6.678  -5.188  1.00 15.00      A    O
ATOM    137  N   SER A 122      14.856   6.194  -3.005  1.00 15.00      A    N
ATOM    138  HN  SER A 122      14.540   5.538  -2.351  1.00 15.00      A    H
ATOM    139  CA  SER A 122      15.409   7.454  -2.529  1.00 15.00      A    C
```

```
ATOM    140  CB  SER A 122      14.323   8.302  -1.861  1.00 15.00      A    C
ATOM    141  OG  SER A 122      14.822   9.575  -1.489  1.00 15.00      A    O
ATOM    142  HG  SER A 122      14.161  10.245  -1.677  1.00 15.00      A    H
ATOM    143  C   SER A 122      16.558   7.187  -1.560  1.00 15.00      A    C
ATOM    144  O   SER A 122      16.354   7.072  -0.349  1.00 15.00      A    O
ATOM    145  N   GLY A 123      17.765   7.073  -2.111  1.00 15.00      A    N
ATOM    146  HN  GLY A 123      17.859   7.177  -3.081  1.00 15.00      A    H
ATOM    147  CA  GLY A 123      18.937   6.799  -1.298  1.00 15.00      A    C
ATOM    148  C   GLY A 123      19.011   5.335  -0.904  1.00 15.00      A    C
ATOM    149  O   GLY A 123      19.164   4.461  -1.759  1.00 15.00      A    O
ATOM    150  N   SER A 124      18.892   5.067   0.392  1.00 15.00      A    N
ATOM    151  HN  SER A 124      18.776   5.806   1.023  1.00 15.00      A    H
ATOM    152  CA  SER A 124      18.927   3.699   0.899  1.00 15.00      A    C
ATOM    153  CB  SER A 124      19.927   3.580   2.052  1.00 15.00      A    C
ATOM    154  OG  SER A 124      21.240   3.892   1.622  1.00 15.00      A    O
ATOM    155  HG  SER A 124      21.848   3.221   1.939  1.00 15.00      A    H
ATOM    156  C   SER A 124      17.538   3.273   1.366  1.00 15.00      A    C
ATOM    157  O   SER A 124      17.367   2.211   1.968  1.00 15.00      A    O
ATOM    158  N   ASN A 125      16.548   4.113   1.074  1.00 15.00      A    N
ATOM    159  HN  ASN A 125      16.753   4.930   0.574  1.00 15.00      A    H
ATOM    160  CA  ASN A 125      15.168   3.853   1.464  1.00 15.00      A    C
ATOM    161  CB  ASN A 125      14.692   4.945   2.428  1.00 15.00      A    C
ATOM    162  CG  ASN A 125      15.605   5.110   3.629  1.00 15.00      A    C
ATOM    163  OD1 ASN A 125      15.945   4.138   4.304  1.00 15.00      A    O
ATOM    164  ND2 ASN A 125      16.021   6.346   3.891  1.00 15.00      A    N
ATOM    165 HD21 ASN A 125      15.721   7.070   3.303  1.00 15.00      A    H
ATOM    166 HD22 ASN A 125      16.612   6.482   4.662  1.00 15.00      A    H
ATOM    167  C   ASN A 125      14.256   3.812   0.240  1.00 15.00      A    C
ATOM    168  O   ASN A 125      14.706   4.024  -0.886  1.00 15.00      A    O
ATOM    169  N   VAL A 126      12.974   3.530   0.469  1.00 15.00      A    N
ATOM    170  HN  VAL A 126      12.682   3.361   1.388  1.00 15.00      A    H
ATOM    171  CA  VAL A 126      11.993   3.465  -0.615  1.00 15.00      A    C
ATOM    172  CB  VAL A 126      11.566   2.012  -0.931  1.00 15.00      A    C
ATOM    173  CG1 VAL A 126      10.448   1.987  -1.965  1.00 15.00      A    C
ATOM    174  CG2 VAL A 126      12.752   1.198  -1.424  1.00 15.00      A    C
ATOM    175  C   VAL A 126      10.752   4.296  -0.288  1.00 15.00      A    C
ATOM    176  O   VAL A 126      10.094   4.078   0.731  1.00 15.00      A    O
ATOM    177  N   VAL A 127      10.444   5.247  -1.163  1.00 15.00      A    N
ATOM    178  HN  VAL A 127      11.013   5.369  -1.952  1.00 15.00      A    H
ATOM    179  CA  VAL A 127       9.279   6.109  -0.989  1.00 15.00      A    C
ATOM    180  CB  VAL A 127       9.500   7.495  -1.632  1.00 15.00      A    C
ATOM    181  CG1 VAL A 127       8.271   8.375  -1.458  1.00 15.00      A    C
ATOM    182  CG2 VAL A 127      10.726   8.170  -1.039  1.00 15.00      A    C
ATOM    183  C   VAL A 127       8.038   5.460  -1.600  1.00 15.00      A    C
ATOM    184  O   VAL A 127       7.915   5.354  -2.821  1.00 15.00      A    O
ATOM    185  N   LEU A 128       7.126   5.023  -0.740  1.00 15.00      A    N
ATOM    186  HN  LEU A 128       7.285   5.135   0.221  1.00 15.00      A    H
```

```
ATOM   187  CA  LEU A 128     5.896   4.380  -1.184  1.00 15.00     A  C
ATOM   188  CB  LEU A 128     5.642   3.105  -0.374  1.00 15.00     A  C
ATOM   189  CG  LEU A 128     4.466   2.235  -0.828  1.00 15.00     A  C
ATOM   190  CD1 LEU A 128     4.737   1.638  -2.200  1.00 15.00     A  C
ATOM   191  CD2 LEU A 128     4.180   1.139   0.189  1.00 15.00     A  C
ATOM   192  C   LEU A 128     4.704   5.331  -1.067  1.00 15.00     A  C
ATOM   193  O   LEU A 128     4.438   5.883  -0.002  1.00 15.00     A  O
ATOM   194  N   ARG A 129     3.999   5.523  -2.176  1.00 15.00     A  N
ATOM   195  HN  ARG A 129     4.271   5.062  -2.998  1.00 15.00     A  H
ATOM   196  CA  ARG A 129     2.828   6.397  -2.208  1.00 15.00     A  C
ATOM   197  CB  ARG A 129     3.051   7.560  -3.181  1.00 15.00     A  C
ATOM   198  CG  ARG A 129     4.244   8.444  -2.849  1.00 15.00     A  C
ATOM   199  CD  ARG A 129     4.433   9.531  -3.897  1.00 15.00     A  C
ATOM   200  NE  ARG A 129     5.572  10.398  -3.593  1.00 15.00     A  N
ATOM   201  HE  ARG A 129     6.311  10.009  -3.079  1.00 15.00     A  H
ATOM   202  CZ  ARG A 129     5.666  11.668  -3.981  1.00 15.00     A  C
ATOM   203  NH1 ARG A 129     4.689  12.228  -4.683  1.00 15.00     A  N
ATOM   204 HH11 ARG A 129     4.766  13.221  -4.982  1.00 15.00     A  H
ATOM   205 HH12 ARG A 129     3.848  11.671  -4.938  1.00 15.00     A  H
ATOM   206  NH2 ARG A 129     6.735  12.384  -3.658  1.00 15.00     A  N
ATOM   207 HH21 ARG A 129     6.808  13.377  -3.958  1.00 15.00     A  H
ATOM   208 HH22 ARG A 129     7.502  11.950  -3.105  1.00 15.00     A  H
ATOM   209  C   ARG A 129     1.596   5.600  -2.624  1.00 15.00     A  C
ATOM   210  O   ARG A 129     1.556   5.040  -3.718  1.00 15.00     A  O
ATOM   211  N   VAL A 130     0.593   5.546  -1.750  1.00 15.00     A  N
ATOM   212  HN  VAL A 130     0.674   6.020  -0.895  1.00 15.00     A  H
ATOM   213  CA  VAL A 130    -0.627   4.794  -2.040  1.00 15.00     A  C
ATOM   214  CB  VAL A 130    -0.823   3.620  -1.055  1.00 15.00     A  C
ATOM   215  CG1 VAL A 130    -2.010   2.766  -1.471  1.00 15.00     A  C
ATOM   216  CG2 VAL A 130     0.437   2.773  -0.958  1.00 15.00     A  C
ATOM   217  C   VAL A 130    -1.874   5.681  -2.015  1.00 15.00     A  C
ATOM   218  O   VAL A 130    -2.257   6.202  -0.970  1.00 15.00     A  O
ATOM   219  N   VAL A 131    -2.506   5.839  -3.174  1.00 15.00     A  N
ATOM   220  HN  VAL A 131    -2.149   5.401  -3.975  1.00 15.00     A  H
ATOM   221  CA  VAL A 131    -3.718   6.648  -3.285  1.00 15.00     A  C
ATOM   222  CB  VAL A 131    -3.625   7.667  -4.442  1.00 15.00     A  C
ATOM   223  CG1 VAL A 131    -4.844   8.579  -4.456  1.00 15.00     A  C
ATOM   224  CG2 VAL A 131    -2.345   8.484  -4.340  1.00 15.00     A  C
ATOM   225  C   VAL A 131    -4.935   5.751  -3.507  1.00 15.00     A  C
ATOM   226  O   VAL A 131    -5.088   5.150  -4.569  1.00 15.00     A  O
ATOM   227  N   SER A 132    -5.799   5.670  -2.502  1.00 15.00     A  N
ATOM   228  HN  SER A 132    -5.633   6.185  -1.685  1.00 15.00     A  H
ATOM   229  CA  SER A 132    -6.993   4.833  -2.588  1.00 15.00     A  C
ATOM   230  CB  SER A 132    -6.722   3.479  -1.932  1.00 15.00     A  C
ATOM   231  OG  SER A 132    -6.252   3.642  -0.605  1.00 15.00     A  O
ATOM   232  HG  SER A 132    -6.076   4.572  -0.440  1.00 15.00     A  H
ATOM   233  C   SER A 132    -8.186   5.500  -1.914  1.00 15.00     A  C
```

```
ATOM 234 O SER A 132 -8.186 6.707 -1.691 1.00 15.00 A O
ATOM 235 N ARG A 133 -9.208 4.705 -1.605 1.00 15.00 A N
ATOM 236 HN ARG A 133 -9.155 3.752 -1.830 1.00 15.00 A H
ATOM 237 CA ARG A 133 -10.404 5.210 -0.942 1.00 15.00 A C
ATOM 238 CB ARG A 133 -11.666 4.606 -1.564 1.00 15.00 A C
ATOM 239 CG ARG A 133 -11.942 5.070 -2.988 1.00 15.00 A C
ATOM 240 CD ARG A 133 -13.221 4.453 -3.529 1.00 15.00 A C
ATOM 241 NE ARG A 133 -13.561 4.952 -4.860 1.00 15.00 A N
ATOM 242 HE ARG A 133 -12.826 5.269 -5.426 1.00 15.00 A H
ATOM 243 CZ ARG A 133 -14.804 5.000 -5.338 1.00 15.00 A C
ATOM 244 NH1 ARG A 133 -15.823 4.578 -4.598 1.00 15.00 A N
ATOM 245 HH11 ARG A 133 -16.791 4.616 -4.976 1.00 15.00 A H
ATOM 246 HH12 ARG A 133 -15.653 4.210 -3.640 1.00 15.00 A H
ATOM 247 NH2 ARG A 133 -15.028 5.466 -6.560 1.00 15.00 A N
ATOM 248 HH21 ARG A 133 -15.999 5.503 -6.933 1.00 15.00 A H
ATOM 249 HH22 ARG A 133 -14.234 5.796 -7.145 1.00 15.00 A H
ATOM 250 C ARG A 133 -10.348 4.915 0.557 1.00 15.00 A C
ATOM 251 O ARG A 133 -9.348 4.394 1.054 1.00 15.00 A O
ATOM 252 N ARG A 134 -11.417 5.249 1.274 1.00 15.00 A N
ATOM 253 HN ARG A 134 -12.187 5.658 0.826 1.00 15.00 A H
ATOM 254 CA ARG A 134 -11.469 5.020 2.716 1.00 15.00 A C
ATOM 255 CB ARG A 134 -12.400 6.025 3.399 1.00 15.00 A C
ATOM 256 CG ARG A 134 -12.002 7.475 3.179 1.00 15.00 A C
ATOM 257 CD ARG A 134 -12.677 8.401 4.175 1.00 15.00 A C
ATOM 258 NE ARG A 134 -12.232 9.784 4.011 1.00 15.00 A N
ATOM 259 HE ARG A 134 -12.063 10.098 3.098 1.00 15.00 A H
ATOM 260 CZ ARG A 134 -12.043 10.630 5.022 1.00 15.00 A C
ATOM 261 NH1 ARG A 134 -12.262 10.242 6.272 1.00 15.00 A N
ATOM 262 HH11 ARG A 134 -12.580 9.271 6.467 1.00 15.00 A H
ATOM 263 HH12 ARG A 134 -12.113 10.907 7.057 1.00 15.00 A H
ATOM 264 NH2 ARG A 134 -11.636 11.869 4.780 1.00 15.00 A N
ATOM 265 HH21 ARG A 134 -11.488 12.532 5.569 1.00 15.00 A H
ATOM 266 HH22 ARG A 134 -11.462 12.179 3.803 1.00 15.00 A H
ATOM 267 C ARG A 134 -11.885 3.590 3.047 1.00 15.00 A C
ATOM 268 O ARG A 134 -13.067 3.249 2.995 1.00 15.00 A O
ATOM 269 N ILE A 135 -10.899 2.759 3.382 1.00 15.00 A N
ATOM 270 HN ILE A 135 -9.979 3.094 3.395 1.00 15.00 A H
ATOM 271 CA ILE A 135 -11.148 1.362 3.728 1.00 15.00 A C
ATOM 272 CB ILE A 135 -10.355 0.400 2.815 1.00 15.00 A C
ATOM 273 CG1 ILE A 135 -10.554 0.775 1.342 1.00 15.00 A C
ATOM 274 CG2 ILE A 135 -10.774 -1.044 3.063 1.00 15.00 A C
ATOM 275 CD1 ILE A 135 -9.742 -0.059 0.375 1.00 15.00 A C
ATOM 276 C ILE A 135 -10.773 1.100 5.187 1.00 15.00 A C
ATOM 277 O ILE A 135 -9.703 1.504 5.644 1.00 15.00 A O
ATOM 278 N VAL A 136 -11.661 0.423 5.912 1.00 15.00 A N
ATOM 279 HN VAL A 136 -12.492 0.124 5.488 1.00 15.00 A H
ATOM 280 CA VAL A 136 -11.430 0.115 7.322 1.00 15.00 A C
```

ATOM	281	CB	VAL	A	136	-12.748	-0.215	8.054	1.00	15.00	A	C
ATOM	282	CG1	VAL	A	136	-12.504	-0.420	9.543	1.00	15.00	A	C
ATOM	283	CG2	VAL	A	136	-13.776	0.883	7.826	1.00	15.00	A	C
ATOM	284	C	VAL	A	136	-10.448	-1.045	7.496	1.00	15.00	A	C
ATOM	285	O	VAL	A	136	-10.685	-2.153	7.012	1.00	15.00	A	O
ATOM	286	N	GLY	A	137	-9.343	-0.773	8.188	1.00	15.00	A	N
ATOM	287	HN	GLY	A	137	-9.209	0.132	8.540	1.00	15.00	A	H
ATOM	288	CA	GLY	A	137	-8.340	-1.796	8.429	1.00	15.00	A	C
ATOM	289	C	GLY	A	137	-7.391	-1.990	7.262	1.00	15.00	A	C
ATOM	290	O	GLY	A	137	-6.608	-2.940	7.247	1.00	15.00	A	O
ATOM	291	N	GLN	A	138	-7.454	-1.090	6.284	1.00	15.00	A	N
ATOM	292	HN	GLN	A	138	-8.097	-0.354	6.351	1.00	15.00	A	H
ATOM	293	CA	GLN	A	138	-6.593	-1.178	5.111	1.00	15.00	A	C
ATOM	294	CB	GLN	A	138	-7.099	-0.265	3.993	1.00	15.00	A	C
ATOM	295	CG	GLN	A	138	-6.335	-0.418	2.688	1.00	15.00	A	C
ATOM	296	CD	GLN	A	138	-6.333	-1.848	2.174	1.00	15.00	A	C
ATOM	297	OE1	GLN	A	138	-7.319	-2.574	2.316	1.00	15.00	A	O
ATOM	298	NE2	GLN	A	138	-5.215	-2.267	1.591	1.00	15.00	A	N
ATOM	299	HE21	GLN	A	138	-4.464	-1.640	1.524	1.00	15.00	A	H
ATOM	300	HE22	GLN	A	138	-5.186	-3.186	1.251	1.00	15.00	A	H
ATOM	301	C	GLN	A	138	-5.143	-0.849	5.455	1.00	15.00	A	C
ATOM	302	O	GLN	A	138	-4.218	-1.437	4.895	1.00	15.00	A	O
ATOM	303	N	LEU	A	139	-4.956	0.094	6.377	1.00	15.00	A	N
ATOM	304	HN	LEU	A	139	-5.737	0.528	6.778	1.00	15.00	A	H
ATOM	305	CA	LEU	A	139	-3.621	0.497	6.807	1.00	15.00	A	C
ATOM	306	CB	LEU	A	139	-3.711	1.653	7.804	1.00	15.00	A	C
ATOM	307	CG	LEU	A	139	-2.388	2.115	8.420	1.00	15.00	A	C
ATOM	308	CD1	LEU	A	139	-1.533	2.835	7.388	1.00	15.00	A	C
ATOM	309	CD2	LEU	A	139	-2.636	2.995	9.633	1.00	15.00	A	C
ATOM	310	C	LEU	A	139	-2.887	-0.679	7.441	1.00	15.00	A	C
ATOM	311	O	LEU	A	139	-1.701	-0.890	7.189	1.00	15.00	A	O
ATOM	312	N	VAL	A	140	-3.612	-1.445	8.255	1.00	15.00	A	N
ATOM	313	HN	VAL	A	140	-4.553	-1.221	8.404	1.00	15.00	A	H
ATOM	314	CA	VAL	A	140	-3.049	-2.610	8.929	1.00	15.00	A	C
ATOM	315	CB	VAL	A	140	-4.043	-3.213	9.948	1.00	15.00	A	C
ATOM	316	CG1	VAL	A	140	-3.384	-4.320	10.759	1.00	15.00	A	C
ATOM	317	CG2	VAL	A	140	-4.588	-2.129	10.868	1.00	15.00	A	C
ATOM	318	C	VAL	A	140	-2.647	-3.680	7.915	1.00	15.00	A	C
ATOM	319	O	VAL	A	140	-1.632	-4.351	8.085	1.00	15.00	A	O
ATOM	320	N	LYS	A	141	-3.450	-3.825	6.859	1.00	15.00	A	N
ATOM	321	HN	LYS	A	141	-4.249	-3.262	6.786	1.00	15.00	A	H
ATOM	322	CA	LYS	A	141	-3.174	-4.801	5.809	1.00	15.00	A	C
ATOM	323	CB	LYS	A	141	-4.342	-4.885	4.825	1.00	15.00	A	C
ATOM	324	CG	LYS	A	141	-5.604	-5.495	5.407	1.00	15.00	A	C
ATOM	325	CD	LYS	A	141	-6.703	-5.585	4.363	1.00	15.00	A	C
ATOM	326	CE	LYS	A	141	-7.959	-6.215	4.939	1.00	15.00	A	C
ATOM	327	NZ	LYS	A	141	-9.016	-6.386	3.906	1.00	15.00	A	N

```
ATOM    328  HZ1 LYS A 141      -8.663  -6.978   3.127  1.00 15.00      A    H
ATOM    329  HZ2 LYS A 141      -9.296  -5.460   3.522  1.00 15.00      A    H
ATOM    330  HZ3 LYS A 141      -9.853  -6.842   4.322  1.00 15.00      A    H
ATOM    331  C   LYS A 141      -1.888  -4.460   5.061  1.00 15.00      A    C
ATOM    332  O   LYS A 141      -1.087  -5.343   4.765  1.00 15.00      A    O
ATOM    333  N   ILE A 142      -1.697  -3.174   4.763  1.00 15.00      A    N
ATOM    334  HN  ILE A 142      -2.376  -2.517   5.025  1.00 15.00      A    H
ATOM    335  CA  ILE A 142      -0.501  -2.719   4.056  1.00 15.00      A    C
ATOM    336  CB  ILE A 142      -0.609  -1.236   3.636  1.00 15.00      A    C
ATOM    337  CG1 ILE A 142      -1.853  -1.019   2.770  1.00 15.00      A    C
ATOM    338  CG2 ILE A 142       0.642  -0.800   2.883  1.00 15.00      A    C
ATOM    339  CD1 ILE A 142      -2.179   0.438   2.519  1.00 15.00      A    C
ATOM    340  C   ILE A 142       0.747  -2.923   4.912  1.00 15.00      A    C
ATOM    341  O   ILE A 142       1.794  -3.328   4.406  1.00 15.00      A    O
ATOM    342  N   ILE A 143       0.627  -2.648   6.211  1.00 15.00      A    N
ATOM    343  HN  ILE A 143      -0.233  -2.323   6.554  1.00 15.00      A    H
ATOM    344  CA  ILE A 143       1.743  -2.817   7.136  1.00 15.00      A    C
ATOM    345  CB  ILE A 143       1.443  -2.204   8.522  1.00 15.00      A    C
ATOM    346  CG1 ILE A 143       1.233  -0.692   8.391  1.00 15.00      A    C
ATOM    347  CG2 ILE A 143       2.573  -2.499   9.503  1.00 15.00      A    C
ATOM    348  CD1 ILE A 143       0.767  -0.020   9.666  1.00 15.00      A    C
ATOM    349  C   ILE A 143       2.116  -4.294   7.275  1.00 15.00      A    C
ATOM    350  O   ILE A 143       3.296  -4.636   7.322  1.00 15.00      A    O
ATOM    351  N   SER A 144       1.105  -5.162   7.324  1.00 15.00      A    N
ATOM    352  HN  SER A 144       0.183  -4.826   7.295  1.00 15.00      A    H
ATOM    353  CA  SER A 144       1.332  -6.602   7.435  1.00 15.00      A    C
ATOM    354  CB  SER A 144       0.015  -7.343   7.661  1.00 15.00      A    C
ATOM    355  OG  SER A 144      -0.586  -6.956   8.883  1.00 15.00      A    O
ATOM    356  HG  SER A 144      -1.505  -7.234   8.890  1.00 15.00      A    H
ATOM    357  C   SER A 144       2.030  -7.144   6.190  1.00 15.00      A    C
ATOM    358  O   SER A 144       2.836  -8.069   6.278  1.00 15.00      A    O
ATOM    359  N   VAL A 145       1.710  -6.563   5.031  1.00 15.00      A    N
ATOM    360  HN  VAL A 145       1.044  -5.843   5.025  1.00 15.00      A    H
ATOM    361  CA  VAL A 145       2.323  -6.972   3.770  1.00 15.00      A    C
ATOM    362  CB  VAL A 145       1.570  -6.388   2.551  1.00 15.00      A    C
ATOM    363  CG1 VAL A 145       2.356  -6.598   1.264  1.00 15.00      A    C
ATOM    364  CG2 VAL A 145       0.190  -7.016   2.427  1.00 15.00      A    C
ATOM    365  C   VAL A 145       3.787  -6.536   3.733  1.00 15.00      A    C
ATOM    366  O   VAL A 145       4.653  -7.284   3.286  1.00 15.00      A    O
ATOM    367  N   LEU A 146       4.051  -5.324   4.221  1.00 15.00      A    N
ATOM    368  HN  LEU A 146       3.314  -4.777   4.564  1.00 15.00      A    H
ATOM    369  CA  LEU A 146       5.407  -4.786   4.256  1.00 15.00      A    C
ATOM    370  CB  LEU A 146       5.393  -3.315   4.680  1.00 15.00      A    C
ATOM    371  CG  LEU A 146       4.781  -2.330   3.679  1.00 15.00      A    C
ATOM    372  CD1 LEU A 146       4.611  -0.959   4.315  1.00 15.00      A    C
ATOM    373  CD2 LEU A 146       5.638  -2.237   2.425  1.00 15.00      A    C
ATOM    374  C   LEU A 146       6.304  -5.600   5.186  1.00 15.00      A    C
```

ATOM	375	O	LEU	A	146	7.418	-5.961	4.816	1.00	15.00	A	O
ATOM	376	N	GLU	A	147	5.806	-5.895	6.390	1.00	15.00	A	N
ATOM	377	HN	GLU	A	147	4.908	-5.579	6.625	1.00	15.00	A	H
ATOM	378	CA	GLU	A	147	6.563	-6.673	7.371	1.00	15.00	A	C
ATOM	379	CB	GLU	A	147	5.824	-6.719	8.711	1.00	15.00	A	C
ATOM	380	CG	GLU	A	147	5.631	-5.359	9.362	1.00	15.00	A	C
ATOM	381	CD	GLU	A	147	4.786	-5.427	10.619	1.00	15.00	A	C
ATOM	382	OE1	GLU	A	147	5.099	-4.697	11.583	1.00	15.00	A	O
ATOM	383	OE2	GLU	A	147	3.812	-6.210	10.639	1.00	15.00	A	O
ATOM	384	C	GLU	A	147	6.832	-8.091	6.869	1.00	15.00	A	C
ATOM	385	O	GLU	A	147	7.871	-8.679	7.174	1.00	15.00	A	O
ATOM	386	N	LYS	A	148	5.887	-8.630	6.097	1.00	15.00	A	N
ATOM	387	HN	LYS	A	148	5.078	-8.111	5.905	1.00	15.00	A	H
ATOM	388	CA	LYS	A	148	6.013	-9.973	5.536	1.00	15.00	A	C
ATOM	389	CB	LYS	A	148	4.656	-10.446	4.999	1.00	15.00	A	C
ATOM	390	CG	LYS	A	148	4.655	-11.856	4.430	1.00	15.00	A	C
ATOM	391	CD	LYS	A	148	3.364	-12.145	3.682	1.00	15.00	A	C
ATOM	392	CE	LYS	A	148	3.430	-13.481	2.962	1.00	15.00	A	C
ATOM	393	NZ	LYS	A	148	2.207	-13.732	2.149	1.00	15.00	A	N
ATOM	394	HZ1	LYS	A	148	2.081	-12.972	1.450	1.00	15.00	A	H
ATOM	395	HZ2	LYS	A	148	1.370	-13.765	2.764	1.00	15.00	A	H
ATOM	396	HZ3	LYS	A	148	2.292	-14.640	1.648	1.00	15.00	A	H
ATOM	397	C	LYS	A	148	7.064	-10.010	4.422	1.00	15.00	A	C
ATOM	398	O	LYS	A	148	7.697	-11.040	4.189	1.00	15.00	A	O
ATOM	399	N	LEU	A	149	7.250	-8.876	3.743	1.00	15.00	A	N
ATOM	400	HN	LEU	A	149	6.720	-8.086	3.980	1.00	15.00	A	H
ATOM	401	CA	LEU	A	149	8.222	-8.780	2.656	1.00	15.00	A	C
ATOM	402	CB	LEU	A	149	7.694	-7.875	1.538	1.00	15.00	A	C
ATOM	403	CG	LEU	A	149	6.332	-8.243	0.936	1.00	15.00	A	C
ATOM	404	CD1	LEU	A	149	6.001	-7.326	-0.232	1.00	15.00	A	C
ATOM	405	CD2	LEU	A	149	6.294	-9.703	0.505	1.00	15.00	A	C
ATOM	406	C	LEU	A	149	9.578	-8.274	3.157	1.00	15.00	A	C
ATOM	407	O	LEU	A	149	10.431	-7.874	2.361	1.00	15.00	A	O
ATOM	408	N	SER	A	150	9.756	-8.295	4.482	1.00	15.00	A	N
ATOM	409	HN	SER	A	150	9.023	-8.616	5.048	1.00	15.00	A	H
ATOM	410	CA	SER	A	150	11.000	-7.862	5.127	1.00	15.00	A	C
ATOM	411	CB	SER	A	150	12.206	-8.660	4.613	1.00	15.00	A	C
ATOM	412	OG	SER	A	150	12.022	-10.051	4.818	1.00	15.00	A	O
ATOM	413	HG	SER	A	150	12.617	-10.354	5.508	1.00	15.00	A	H
ATOM	414	C	SER	A	150	11.248	-6.354	5.013	1.00	15.00	A	C
ATOM	415	O	SER	A	150	12.393	-5.914	4.921	1.00	15.00	A	O
ATOM	416	N	PHE	A	151	10.175	-5.565	5.033	1.00	15.00	A	N
ATOM	417	HN	PHE	A	151	9.285	-5.970	5.098	1.00	15.00	A	H
ATOM	418	CA	PHE	A	151	10.290	-4.111	4.953	1.00	15.00	A	C
ATOM	419	CB	PHE	A	151	9.333	-3.538	3.905	1.00	15.00	A	C
ATOM	420	CG	PHE	A	151	9.809	-3.689	2.490	1.00	15.00	A	C
ATOM	421	CD1	PHE	A	151	10.755	-2.823	1.967	1.00	15.00	A	C

```
ATOM    422  CD2 PHE A 151       9.305  -4.693   1.681  1.00 15.00      A    C
ATOM    423  CE1 PHE A 151      11.193  -2.956   0.663  1.00 15.00      A    C
ATOM    424  CE2 PHE A 151       9.737  -4.833   0.377  1.00 15.00      A    C
ATOM    425  CZ  PHE A 151      10.682  -3.963  -0.134  1.00 15.00      A    C
ATOM    426  C   PHE A 151      10.012  -3.468   6.306  1.00 15.00      A    C
ATOM    427  O   PHE A 151       8.993  -3.747   6.938  1.00 15.00      A    O
ATOM    428  N   GLN A 152      10.922  -2.606   6.745  1.00 15.00      A    N
ATOM    429  HN  GLN A 152      11.716  -2.426   6.197  1.00 15.00      A    H
ATOM    430  CA  GLN A 152      10.766  -1.918   8.020  1.00 15.00      A    C
ATOM    431  CB  GLN A 152      12.092  -1.882   8.786  1.00 15.00      A    C
ATOM    432  CG  GLN A 152      12.026  -1.113  10.098  1.00 15.00      A    C
ATOM    433  CD  GLN A 152      13.363  -1.039  10.808  1.00 15.00      A    C
ATOM    434  OE1 GLN A 152      14.420  -1.066  10.177  1.00 15.00      A    O
ATOM    435  NE2 GLN A 152      13.320  -0.936  12.132  1.00 15.00      A    N
ATOM    436 HE21 GLN A 152      12.440  -0.915  12.565  1.00 15.00      A    H
ATOM    437 HE22 GLN A 152      14.167  -0.887  12.622  1.00 15.00      A    H
ATOM    438  C   GLN A 152      10.241  -0.502   7.810  1.00 15.00      A    C
ATOM    439  O   GLN A 152      10.874   0.311   7.136  1.00 15.00      A    O
ATOM    440  N   VAL A 153       9.079  -0.215   8.386  1.00 15.00      A    N
ATOM    441  HN  VAL A 153       8.618  -0.909   8.901  1.00 15.00      A    H
ATOM    442  CA  VAL A 153       8.471   1.106   8.268  1.00 15.00      A    C
ATOM    443  CB  VAL A 153       6.971   1.073   8.634  1.00 15.00      A    C
ATOM    444  CG1 VAL A 153       6.318   2.421   8.359  1.00 15.00      A    C
ATOM    445  CG2 VAL A 153       6.253  -0.035   7.877  1.00 15.00      A    C
ATOM    446  C   VAL A 153       9.187   2.113   9.165  1.00 15.00      A    C
ATOM    447  O   VAL A 153       9.192   1.973  10.388  1.00 15.00      A    O
ATOM    448  N   LEU A 154       9.796   3.122   8.551  1.00 15.00      A    N
ATOM    449  HN  LEU A 154       9.766   3.175   7.573  1.00 15.00      A    H
ATOM    450  CA  LEU A 154      10.510   4.152   9.298  1.00 15.00      A    C
ATOM    451  CB  LEU A 154      11.772   4.588   8.547  1.00 15.00      A    C
ATOM    452  CG  LEU A 154      12.730   3.477   8.105  1.00 15.00      A    C
ATOM    453  CD1 LEU A 154      13.798   4.039   7.180  1.00 15.00      A    C
ATOM    454  CD2 LEU A 154      13.369   2.801   9.311  1.00 15.00      A    C
ATOM    455  C   LEU A 154       9.612   5.359   9.554  1.00 15.00      A    C
ATOM    456  O   LEU A 154       9.632   5.939  10.640  1.00 15.00      A    O
ATOM    457  N   HIS A 155       8.822   5.731   8.549  1.00 15.00      A    N
ATOM    458  HN  HIS A 155       8.848   5.225   7.710  1.00 15.00      A    H
ATOM    459  CA  HIS A 155       7.917   6.871   8.665  1.00 15.00      A    C
ATOM    460  CB  HIS A 155       8.575   8.138   8.098  1.00 15.00      A    C
ATOM    461  CG  HIS A 155       7.751   9.385   8.248  1.00 15.00      A    C
ATOM    462  ND1 HIS A 155       6.975   9.857   7.217  1.00 15.00      A    N
ATOM    463  CD2 HIS A 155       7.621  10.210   9.318  1.00 15.00      A    C
ATOM    464  CE1 HIS A 155       6.391  10.950   7.674  1.00 15.00      A    C
ATOM    465  NE2 HIS A 155       6.752  11.202   8.939  1.00 15.00      A    N
ATOM    466  HE2 HIS A 155       6.453  11.955   9.491  1.00 15.00      A    H
ATOM    467  C   HIS A 155       6.592   6.593   7.958  1.00 15.00      A    C
ATOM    468  O   HIS A 155       6.558   5.949   6.910  1.00 15.00      A    O
```

```
ATOM    469  N   LEU A 156       5.504   7.087   8.545  1.00 15.00       A   N
ATOM    470  HN  LEU A 156       5.602   7.586   9.382  1.00 15.00       A   H
ATOM    471  CA  LEU A 156       4.173   6.905   7.981  1.00 15.00       A   C
ATOM    472  CB  LEU A 156       3.477   5.700   8.628  1.00 15.00       A   C
ATOM    473  CG  LEU A 156       1.958   5.585   8.438  1.00 15.00       A   C
ATOM    474  CD1 LEU A 156       1.588   5.538   6.961  1.00 15.00       A   C
ATOM    475  CD2 LEU A 156       1.421   4.362   9.166  1.00 15.00       A   C
ATOM    476  C   LEU A 156       3.326   8.168   8.137  1.00 15.00       A   C
ATOM    477  O   LEU A 156       3.098   8.646   9.249  1.00 15.00       A   O
ATOM    478  N   ASN A 157       2.867   8.700   7.006  1.00 15.00       A   N
ATOM    479  HN  ASN A 157       3.090   8.267   6.155  1.00 15.00       A   H
ATOM    480  CA  ASN A 157       2.036   9.899   6.990  1.00 15.00       A   C
ATOM    481  CB  ASN A 157       2.759  11.044   6.269  1.00 15.00       A   C
ATOM    482  CG  ASN A 157       1.913  12.300   6.153  1.00 15.00       A   C
ATOM    483  OD1 ASN A 157       1.257  12.528   5.136  1.00 15.00       A   O
ATOM    484  ND2 ASN A 157       1.920  13.121   7.199  1.00 15.00       A   N
ATOM    485 HD21 ASN A 157       2.462  12.876   7.978  1.00 15.00       A   H
ATOM    486 HD22 ASN A 157       1.380  13.939   7.148  1.00 15.00       A   H
ATOM    487  C   ASN A 157       0.695   9.602   6.321  1.00 15.00       A   C
ATOM    488  O   ASN A 157       0.649   9.155   5.175  1.00 15.00       A   O
ATOM    489  N   ILE A 158      -0.395   9.847   7.044  1.00 15.00       A   N
ATOM    490  HN  ILE A 158      -0.298  10.208   7.949  1.00 15.00       A   H
ATOM    491  CA  ILE A 158      -1.734   9.590   6.518  1.00 15.00       A   C
ATOM    492  CB  ILE A 158      -2.553   8.686   7.466  1.00 15.00       A   C
ATOM    493  CG1 ILE A 158      -1.774   7.411   7.804  1.00 15.00       A   C
ATOM    494  CG2 ILE A 158      -3.899   8.339   6.841  1.00 15.00       A   C
ATOM    495  CD1 ILE A 158      -2.359   6.621   8.955  1.00 15.00       A   C
ATOM    496  C   ILE A 158      -2.506  10.885   6.272  1.00 15.00       A   C
ATOM    497  O   ILE A 158      -2.870  11.592   7.212  1.00 15.00       A   O
ATOM    498  N   SER A 159      -2.755  11.185   5.000  1.00 15.00       A   N
ATOM    499  HN  SER A 159      -2.427  10.585   4.297  1.00 15.00       A   H
ATOM    500  CA  SER A 159      -3.502  12.383   4.620  1.00 15.00       A   C
ATOM    501  CB  SER A 159      -2.650  13.287   3.725  1.00 15.00       A   C
ATOM    502  OG  SER A 159      -1.486  13.726   4.403  1.00 15.00       A   O
ATOM    503  HG  SER A 159      -1.707  13.941   5.311  1.00 15.00       A   H
ATOM    504  C   SER A 159      -4.787  11.993   3.897  1.00 15.00       A   C
ATOM    505  O   SER A 159      -4.751  11.268   2.907  1.00 15.00       A   O
ATOM    506  N   SER A 160      -5.924  12.471   4.391  1.00 15.00       A   N
ATOM    507  HN  SER A 160      -5.901  13.053   5.180  1.00 15.00       A   H
ATOM    508  CA  SER A 160      -7.206  12.143   3.778  1.00 15.00       A   C
ATOM    509  CB  SER A 160      -8.119  11.427   4.778  1.00 15.00       A   C
ATOM    510  OG  SER A 160      -8.411  12.258   5.887  1.00 15.00       A   O
ATOM    511  HG  SER A 160      -8.543  13.159   5.585  1.00 15.00       A   H
ATOM    512  C   SER A 160      -7.905  13.372   3.201  1.00 15.00       A   C
ATOM    513  O   SER A 160      -7.861  14.458   3.782  1.00 15.00       A   O
ATOM    514  N   MET A 161      -8.548  13.183   2.051  1.00 15.00       A   N
ATOM    515  HN  MET A 161      -8.532  12.295   1.639  1.00 15.00       A   H
```

ATOM	516	CA	MET	A	161	-9.274	14.254	1.380	1.00	15.00	A	C
ATOM	517	CB	MET	A	161	-8.393	14.924	0.322	1.00	15.00	A	C
ATOM	518	CG	MET	A	161	-8.984	16.204	-0.248	1.00	15.00	A	C
ATOM	519	SD	MET	A	161	-8.046	16.852	-1.644	1.00	15.00	A	S
ATOM	520	CE	MET	A	161	-8.365	15.594	-2.879	1.00	15.00	A	C
ATOM	521	C	MET	A	161	-10.549	13.709	0.737	1.00	15.00	A	C
ATOM	522	O	MET	A	161	-10.485	12.901	-0.191	1.00	15.00	A	O
ATOM	523	N	GLU	A	162	-11.700	14.154	1.249	1.00	15.00	A	N
ATOM	524	HN	GLU	A	162	-11.668	14.794	1.989	1.00	15.00	A	H
ATOM	525	CA	GLU	A	162	-13.009	13.725	0.747	1.00	15.00	A	C
ATOM	526	CB	GLU	A	162	-13.204	14.094	-0.729	1.00	15.00	A	C
ATOM	527	CG	GLU	A	162	-13.178	15.587	-1.006	1.00	15.00	A	C
ATOM	528	CD	GLU	A	162	-13.264	15.904	-2.485	1.00	15.00	A	C
ATOM	529	OE1	GLU	A	162	-12.204	15.953	-3.143	1.00	15.00	A	O
ATOM	530	OE2	GLU	A	162	-14.391	16.101	-2.985	1.00	15.00	A	O
ATOM	531	C	GLU	A	162	-13.261	12.234	0.984	1.00	15.00	A	C
ATOM	532	O	GLU	A	162	-13.591	11.822	2.097	1.00	15.00	A	O
ATOM	533	N	GLU	A	163	-13.098	11.430	-0.064	1.00	15.00	A	N
ATOM	534	HN	GLU	A	163	-12.829	11.814	-0.924	1.00	15.00	A	H
ATOM	535	CA	GLU	A	163	-13.313	9.989	0.033	1.00	15.00	A	C
ATOM	536	CB	GLU	A	163	-14.399	9.540	-0.951	1.00	15.00	A	C
ATOM	537	CG	GLU	A	163	-15.776	10.128	-0.683	1.00	15.00	A	C
ATOM	538	CD	GLU	A	163	-16.813	9.667	-1.690	1.00	15.00	A	C
ATOM	539	OE1	GLU	A	163	-16.956	10.328	-2.741	1.00	15.00	A	O
ATOM	540	OE2	GLU	A	163	-17.481	8.645	-1.429	1.00	15.00	A	O
ATOM	541	C	GLU	A	163	-12.025	9.211	-0.230	1.00	15.00	A	C
ATOM	542	O	GLU	A	163	-12.018	7.982	-0.185	1.00	15.00	A	O
ATOM	543	N	THR	A	164	-10.936	9.928	-0.500	1.00	15.00	A	N
ATOM	544	HN	THR	A	164	-10.995	10.906	-0.511	1.00	15.00	A	H
ATOM	545	CA	THR	A	164	-9.653	9.288	-0.781	1.00	15.00	A	C
ATOM	546	CB	THR	A	164	-9.009	9.844	-2.067	1.00	15.00	A	C
ATOM	547	OG1	THR	A	164	-8.811	11.257	-1.937	1.00	15.00	A	O
ATOM	548	HG1	THR	A	164	-8.431	11.449	-1.077	1.00	15.00	A	H
ATOM	549	CG2	THR	A	164	-9.889	9.564	-3.277	1.00	15.00	A	C
ATOM	550	C	THR	A	164	-8.667	9.438	0.375	1.00	15.00	A	C
ATOM	551	O	THR	A	164	-8.721	10.406	1.132	1.00	15.00	A	O
ATOM	552	N	VAL	A	165	-7.770	8.460	0.502	1.00	15.00	A	N
ATOM	553	HN	VAL	A	165	-7.796	7.710	-0.129	1.00	15.00	A	H
ATOM	554	CA	VAL	A	165	-6.750	8.466	1.550	1.00	15.00	A	C
ATOM	555	CB	VAL	A	165	-7.046	7.434	2.662	1.00	15.00	A	C
ATOM	556	CG1	VAL	A	165	-6.024	7.548	3.783	1.00	15.00	A	C
ATOM	557	CG2	VAL	A	165	-8.451	7.614	3.214	1.00	15.00	A	C
ATOM	558	C	VAL	A	165	-5.372	8.174	0.956	1.00	15.00	A	C
ATOM	559	O	VAL	A	165	-5.192	7.199	0.225	1.00	15.00	A	O
ATOM	560	N	LEU	A	166	-4.407	9.027	1.279	1.00	15.00	A	N
ATOM	561	HN	LEU	A	166	-4.618	9.778	1.872	1.00	15.00	A	H
ATOM	562	CA	LEU	A	166	-3.043	8.879	0.789	1.00	15.00	A	C

ATOM	563	CB	LEU	A	166	-2.517	10.232	0.294	1.00	15.00	A	C
ATOM	564	CG	LEU	A	166	-1.016	10.326	0.005	1.00	15.00	A	C
ATOM	565	CD1	LEU	A	166	-0.632	9.457	-1.183	1.00	15.00	A	C
ATOM	566	CD2	LEU	A	166	-0.609	11.773	-0.231	1.00	15.00	A	C
ATOM	567	C	LEU	A	166	-2.127	8.321	1.876	1.00	15.00	A	C
ATOM	568	O	LEU	A	166	-1.941	8.939	2.925	1.00	15.00	A	O
ATOM	569	N	TYR	A	167	-1.559	7.147	1.613	1.00	15.00	A	N
ATOM	570	HN	TYR	A	167	-1.750	6.707	0.759	1.00	15.00	A	H
ATOM	571	CA	TYR	A	167	-0.659	6.499	2.559	1.00	15.00	A	C
ATOM	572	CB	TYR	A	167	-0.979	5.007	2.667	1.00	15.00	A	C
ATOM	573	CG	TYR	A	167	-2.365	4.691	3.187	1.00	15.00	A	C
ATOM	574	CD1	TYR	A	167	-2.689	4.875	4.526	1.00	15.00	A	C
ATOM	575	CD2	TYR	A	167	-3.348	4.194	2.338	1.00	15.00	A	C
ATOM	576	CE1	TYR	A	167	-3.952	4.576	5.004	1.00	15.00	A	C
ATOM	577	CE2	TYR	A	167	-4.613	3.895	2.806	1.00	15.00	A	C
ATOM	578	CZ	TYR	A	167	-4.909	4.086	4.139	1.00	15.00	A	C
ATOM	579	OH	TYR	A	167	-6.167	3.785	4.609	1.00	15.00	A	O
ATOM	580	HH	TYR	A	167	-6.452	2.946	4.244	1.00	15.00	A	H
ATOM	581	C	TYR	A	167	0.800	6.686	2.140	1.00	15.00	A	C
ATOM	582	O	TYR	A	167	1.279	6.037	1.208	1.00	15.00	A	O
ATOM	583	N	PHE	A	168	1.499	7.574	2.837	1.00	15.00	A	N
ATOM	584	HN	PHE	A	168	1.060	8.052	3.570	1.00	15.00	A	H
ATOM	585	CA	PHE	A	168	2.902	7.851	2.550	1.00	15.00	A	C
ATOM	586	CB	PHE	A	168	3.196	9.344	2.738	1.00	15.00	A	C
ATOM	587	CG	PHE	A	168	4.562	9.772	2.282	1.00	15.00	A	C
ATOM	588	CD1	PHE	A	168	5.560	10.046	3.204	1.00	15.00	A	C
ATOM	589	CD2	PHE	A	168	4.847	9.904	0.932	1.00	15.00	A	C
ATOM	590	CE1	PHE	A	168	6.817	10.444	2.788	1.00	15.00	A	C
ATOM	591	CE2	PHE	A	168	6.101	10.301	0.509	1.00	15.00	A	C
ATOM	592	CZ	PHE	A	168	7.088	10.571	1.439	1.00	15.00	A	C
ATOM	593	C	PHE	A	168	3.806	7.008	3.448	1.00	15.00	A	C
ATOM	594	O	PHE	A	168	3.800	7.167	4.667	1.00	15.00	A	O
ATOM	595	N	PHE	A	169	4.579	6.113	2.838	1.00	15.00	A	N
ATOM	596	HN	PHE	A	169	4.551	6.042	1.862	1.00	15.00	A	H
ATOM	597	CA	PHE	A	169	5.471	5.237	3.589	1.00	15.00	A	C
ATOM	598	CB	PHE	A	169	5.111	3.765	3.352	1.00	15.00	A	C
ATOM	599	CG	PHE	A	169	3.801	3.321	3.936	1.00	15.00	A	C
ATOM	600	CD1	PHE	A	169	3.736	2.826	5.230	1.00	15.00	A	C
ATOM	601	CD2	PHE	A	169	2.640	3.379	3.185	1.00	15.00	A	C
ATOM	602	CE1	PHE	A	169	2.534	2.399	5.762	1.00	15.00	A	C
ATOM	603	CE2	PHE	A	169	1.436	2.957	3.713	1.00	15.00	A	C
ATOM	604	CZ	PHE	A	169	1.383	2.465	5.002	1.00	15.00	A	C
ATOM	605	C	PHE	A	169	6.936	5.431	3.221	1.00	15.00	A	C
ATOM	606	O	PHE	A	169	7.288	5.557	2.050	1.00	15.00	A	O
ATOM	607	N	VAL	A	170	7.781	5.457	4.243	1.00	15.00	A	N
ATOM	608	HN	VAL	A	170	7.420	5.403	5.154	1.00	15.00	A	H
ATOM	609	CA	VAL	A	170	9.226	5.564	4.062	1.00	15.00	A	C

ATOM 610 CB VAL A 170 9.814 6.811 4.760 1.00 15.00 A C
ATOM 611 CG1 VAL A 170 11.311 6.921 4.496 1.00 15.00 A C
ATOM 612 CG2 VAL A 170 9.104 8.074 4.297 1.00 15.00 A C
ATOM 613 C VAL A 170 9.839 4.304 4.664 1.00 15.00 A C
ATOM 614 O VAL A 170 10.205 4.274 5.837 1.00 15.00 A O
ATOM 615 N VAL A 171 9.916 3.253 3.856 1.00 15.00 A N
ATOM 616 HN VAL A 171 9.631 3.346 2.924 1.00 15.00 A H
ATOM 617 CA VAL A 171 10.422 1.966 4.322 1.00 15.00 A C
ATOM 618 CB VAL A 171 9.577 0.794 3.778 1.00 15.00 A C
ATOM 619 CG1 VAL A 171 8.185 0.818 4.384 1.00 15.00 A C
ATOM 620 CG2 VAL A 171 9.497 0.839 2.257 1.00 15.00 A C
ATOM 621 C VAL A 171 11.890 1.715 3.997 1.00 15.00 A C
ATOM 622 O VAL A 171 12.439 2.249 3.034 1.00 15.00 A O
ATOM 623 N LYS A 172 12.507 0.886 4.831 1.00 15.00 A N
ATOM 624 HN LYS A 172 12.005 0.528 5.593 1.00 15.00 A H
ATOM 625 CA LYS A 172 13.895 0.489 4.666 1.00 15.00 A C
ATOM 626 CB LYS A 172 14.629 0.584 6.010 1.00 15.00 A C
ATOM 627 CG LYS A 172 16.077 0.116 5.982 1.00 15.00 A C
ATOM 628 CD LYS A 172 16.663 0.052 7.385 1.00 15.00 A C
ATOM 629 CE LYS A 172 18.089 -0.474 7.369 1.00 15.00 A C
ATOM 630 NZ LYS A 172 18.666 -0.563 8.741 1.00 15.00 A N
ATOM 631 HZ1 LYS A 172 18.092 -1.201 9.329 1.00 15.00 A H
ATOM 632 HZ2 LYS A 172 18.681 0.378 9.184 1.00 15.00 A H
ATOM 633 HZ3 LYS A 172 19.639 -0.929 8.697 1.00 15.00 A H
ATOM 634 C LYS A 172 13.929 -0.948 4.153 1.00 15.00 A C
ATOM 635 O LYS A 172 13.222 -1.812 4.675 1.00 15.00 A O
ATOM 636 N ILE A 173 14.734 -1.196 3.125 1.00 15.00 A N
ATOM 637 HN ILE A 173 15.267 -0.466 2.749 1.00 15.00 A H
ATOM 638 CA ILE A 173 14.841 -2.531 2.542 1.00 15.00 A C
ATOM 639 CB ILE A 173 15.525 -2.495 1.159 1.00 15.00 A C
ATOM 640 CG1 ILE A 173 14.888 -1.421 0.271 1.00 15.00 A C
ATOM 641 CG2 ILE A 173 15.440 -3.861 0.489 1.00 15.00 A C
ATOM 642 CD1 ILE A 173 15.645 -1.157 -1.014 1.00 15.00 A C
ATOM 643 C ILE A 173 15.612 -3.479 3.459 1.00 15.00 A C
ATOM 644 O ILE A 173 16.757 -3.209 3.827 1.00 15.00 A O
ATOM 645 N GLY A 174 14.972 -4.589 3.824 1.00 15.00 A N
ATOM 646 HN GLY A 174 14.057 -4.740 3.508 1.00 15.00 A H
ATOM 647 CA GLY A 174 15.607 -5.572 4.685 1.00 15.00 A C
ATOM 648 C GLY A 174 16.628 -6.413 3.941 1.00 15.00 A C
ATOM 649 O GLY A 174 16.657 -6.421 2.710 1.00 15.00 A O
ATOM 650 N LEU A 175 17.468 -7.123 4.688 1.00 15.00 A N
ATOM 651 HN LEU A 175 17.395 -7.077 5.664 1.00 15.00 A H
ATOM 652 CA LEU A 175 18.501 -7.967 4.093 1.00 15.00 A C
ATOM 653 CB LEU A 175 19.560 -8.328 5.139 1.00 15.00 A C
ATOM 654 CG LEU A 175 20.297 -7.154 5.791 1.00 15.00 A C
ATOM 655 CD1 LEU A 175 21.180 -7.646 6.927 1.00 15.00 A C
ATOM 656 CD2 LEU A 175 21.122 -6.396 4.762 1.00 15.00 A C

ATOM	657	C	LEU	A	175	17.916	-9.239	3.482	1.00	15.00	A	C
ATOM	658	O	LEU	A	175	18.550	-9.882	2.646	1.00	15.00	A	O
ATOM	659	N	GLU	A	176	16.703	-9.596	3.899	1.00	15.00	A	N
ATOM	660	HN	GLU	A	176	16.243	-9.042	4.563	1.00	15.00	A	H
ATOM	661	CA	GLU	A	176	16.045	-10.797	3.393	1.00	15.00	A	C
ATOM	662	CB	GLU	A	176	15.372	-11.558	4.536	1.00	15.00	A	C
ATOM	663	CG	GLU	A	176	16.328	-11.982	5.641	1.00	15.00	A	C
ATOM	664	CD	GLU	A	176	15.627	-12.684	6.789	1.00	15.00	A	C
ATOM	665	OE1	GLU	A	176	16.324	-13.317	7.610	1.00	15.00	A	O
ATOM	666	OE2	GLU	A	176	14.383	-12.599	6.868	1.00	15.00	A	O
ATOM	667	C	GLU	A	176	15.031	-10.475	2.292	1.00	15.00	A	C
ATOM	668	O	GLU	A	176	14.235	-11.330	1.905	1.00	15.00	A	O
ATOM	669	N	CYS	A	177	15.067	-9.244	1.787	1.00	15.00	A	N
ATOM	670	HN	CYS	A	177	15.724	-8.604	2.130	1.00	15.00	A	H
ATOM	671	CA	CYS	A	177	14.152	-8.828	0.730	1.00	15.00	A	C
ATOM	672	CB	CYS	A	177	13.856	-7.329	0.826	1.00	15.00	A	C
ATOM	673	SG	CYS	A	177	12.731	-6.710	-0.447	1.00	15.00	A	S
ATOM	674	HG	CYS	A	177	11.780	-6.008	0.152	1.00	15.00	A	H
ATOM	675	C	CYS	A	177	14.716	-9.167	-0.647	1.00	15.00	A	C
ATOM	676	O	CYS	A	177	15.792	-8.697	-1.020	1.00	15.00	A	O
ATOM	677	N	HIS	A	178	13.980	-9.984	-1.397	1.00	15.00	A	N
ATOM	678	HN	HIS	A	178	13.129	-10.317	-1.043	1.00	15.00	A	H
ATOM	679	CA	HIS	A	178	14.405	-10.398	-2.733	1.00	15.00	A	C
ATOM	680	CB	HIS	A	178	14.559	-11.924	-2.798	1.00	15.00	A	C
ATOM	681	CG	HIS	A	178	15.463	-12.500	-1.747	1.00	15.00	A	C
ATOM	682	ND1	HIS	A	178	16.826	-12.337	-1.808	1.00	15.00	A	N
ATOM	683	CD2	HIS	A	178	15.147	-13.217	-0.641	1.00	15.00	A	C
ATOM	684	CE1	HIS	A	178	17.311	-12.952	-0.743	1.00	15.00	A	C
ATOM	685	NE2	HIS	A	178	16.333	-13.499	-0.010	1.00	15.00	A	N
ATOM	686	HE2	HIS	A	178	16.443	-14.007	0.822	1.00	15.00	A	H
ATOM	687	C	HIS	A	178	13.410	-9.939	-3.798	1.00	15.00	A	C
ATOM	688	O	HIS	A	178	13.467	-10.386	-4.945	1.00	15.00	A	O
ATOM	689	N	LEU	A	179	12.503	-9.043	-3.417	1.00	15.00	A	N
ATOM	690	HN	LEU	A	179	12.517	-8.713	-2.494	1.00	15.00	A	H
ATOM	691	CA	LEU	A	179	11.491	-8.537	-4.340	1.00	15.00	A	C
ATOM	692	CB	LEU	A	179	10.127	-8.456	-3.649	1.00	15.00	A	C
ATOM	693	CG	LEU	A	179	9.572	-9.775	-3.110	1.00	15.00	A	C
ATOM	694	CD1	LEU	A	179	9.318	-9.672	-1.616	1.00	15.00	A	C
ATOM	695	CD2	LEU	A	179	8.298	-10.160	-3.848	1.00	15.00	A	C
ATOM	696	C	LEU	A	179	11.873	-7.177	-4.914	1.00	15.00	A	C
ATOM	697	O	LEU	A	179	12.573	-6.392	-4.274	1.00	15.00	A	O
ATOM	698	N	SER	A	180	11.400	-6.912	-6.129	1.00	15.00	A	N
ATOM	699	HN	SER	A	180	10.856	-7.589	-6.585	1.00	15.00	A	H
ATOM	700	CA	SER	A	180	11.671	-5.649	-6.807	1.00	15.00	A	C
ATOM	701	CB	SER	A	180	11.753	-5.868	-8.321	1.00	15.00	A	C
ATOM	702	OG	SER	A	180	10.523	-6.350	-8.839	1.00	15.00	A	O
ATOM	703	HG	SER	A	180	10.287	-5.848	-9.623	1.00	15.00	A	H

ATOM	704	C	SER	A	180	10.588	-4.620	-6.488	1.00	15.00	A	C
ATOM	705	O	SER	A	180	9.648	-4.906	-5.745	1.00	15.00	A	O
ATOM	706	N	LEU	A	181	10.723	-3.423	-7.054	1.00	15.00	A	N
ATOM	707	HN	LEU	A	181	11.495	-3.252	-7.633	1.00	15.00	A	H
ATOM	708	CA	LEU	A	181	9.751	-2.358	-6.834	1.00	15.00	A	C
ATOM	709	CB	LEU	A	181	10.288	-1.024	-7.352	1.00	15.00	A	C
ATOM	710	CG	LEU	A	181	11.514	-0.458	-6.636	1.00	15.00	A	C
ATOM	711	CD1	LEU	A	181	11.924	0.863	-7.265	1.00	15.00	A	C
ATOM	712	CD2	LEU	A	181	11.238	-0.284	-5.149	1.00	15.00	A	C
ATOM	713	C	LEU	A	181	8.429	-2.682	-7.518	1.00	15.00	A	C
ATOM	714	O	LEU	A	181	7.364	-2.273	-7.056	1.00	15.00	A	O
ATOM	715	N	GLU	A	182	8.510	-3.420	-8.622	1.00	15.00	A	N
ATOM	716	HN	GLU	A	182	9.393	-3.708	-8.939	1.00	15.00	A	H
ATOM	717	CA	GLU	A	182	7.328	-3.812	-9.377	1.00	15.00	A	C
ATOM	718	CB	GLU	A	182	7.721	-4.363	-10.752	1.00	15.00	A	C
ATOM	719	CG	GLU	A	182	8.259	-3.323	-11.728	1.00	15.00	A	C
ATOM	720	CD	GLU	A	182	9.693	-2.917	-11.441	1.00	15.00	A	C
ATOM	721	OE1	GLU	A	182	10.039	-1.743	-11.699	1.00	15.00	A	O
ATOM	722	OE2	GLU	A	182	10.468	-3.772	-10.956	1.00	15.00	A	O
ATOM	723	C	GLU	A	182	6.516	-4.852	-8.614	1.00	15.00	A	C
ATOM	724	O	GLU	A	182	5.289	-4.790	-8.585	1.00	15.00	A	O
ATOM	725	N	GLU	A	183	7.214	-5.803	-7.990	1.00	15.00	A	N
ATOM	726	HN	GLU	A	183	8.192	-5.797	-8.055	1.00	15.00	A	H
ATOM	727	CA	GLU	A	183	6.563	-6.857	-7.217	1.00	15.00	A	C
ATOM	728	CB	GLU	A	183	7.540	-8.000	-6.942	1.00	15.00	A	C
ATOM	729	CG	GLU	A	183	8.071	-8.677	-8.194	1.00	15.00	A	C
ATOM	730	CD	GLU	A	183	9.051	-9.788	-7.882	1.00	15.00	A	C
ATOM	731	OE1	GLU	A	183	8.688	-10.969	-8.069	1.00	15.00	A	O
ATOM	732	OE2	GLU	A	183	10.181	-9.477	-7.446	1.00	15.00	A	O
ATOM	733	C	GLU	A	183	6.004	-6.314	-5.904	1.00	15.00	A	C
ATOM	734	O	GLU	A	183	5.039	-6.853	-5.362	1.00	15.00	A	O
ATOM	735	N	LEU	A	184	6.624	-5.248	-5.398	1.00	15.00	A	N
ATOM	736	HN	LEU	A	184	7.398	-4.878	-5.873	1.00	15.00	A	H
ATOM	737	CA	LEU	A	184	6.189	-4.618	-4.157	1.00	15.00	A	C
ATOM	738	CB	LEU	A	184	7.261	-3.648	-3.645	1.00	15.00	A	C
ATOM	739	CG	LEU	A	184	6.887	-2.792	-2.430	1.00	15.00	A	C
ATOM	740	CD1	LEU	A	184	6.681	-3.659	-1.197	1.00	15.00	A	C
ATOM	741	CD2	LEU	A	184	7.946	-1.730	-2.174	1.00	15.00	A	C
ATOM	742	C	LEU	A	184	4.865	-3.885	-4.359	1.00	15.00	A	C
ATOM	743	O	LEU	A	184	3.951	-4.004	-3.545	1.00	15.00	A	O
ATOM	744	N	THR	A	185	4.767	-3.136	-5.457	1.00	15.00	A	N
ATOM	745	HN	THR	A	185	5.529	-3.086	-6.070	1.00	15.00	A	H
ATOM	746	CA	THR	A	185	3.553	-2.387	-5.770	1.00	15.00	A	C
ATOM	747	CB	THR	A	185	3.768	-1.385	-6.925	1.00	15.00	A	C
ATOM	748	OG1	THR	A	185	4.258	-2.072	-8.083	1.00	15.00	A	O
ATOM	749	HG1	THR	A	185	5.104	-2.480	-7.880	1.00	15.00	A	H
ATOM	750	CG2	THR	A	185	4.758	-0.302	-6.522	1.00	15.00	A	C

ATOM	751	C	THR	A	185	2.391	-3.317	-6.114	1.00	15.00	A	C
ATOM	752	O	THR	A	185	1.230	-2.974	-5.895	1.00	15.00	A	O
ATOM	753	N	LEU	A	186	2.710	-4.495	-6.655	1.00	15.00	A	N
ATOM	754	HN	LEU	A	186	3.654	-4.707	-6.814	1.00	15.00	A	H
ATOM	755	CA	LEU	A	186	1.692	-5.474	-7.020	1.00	15.00	A	C
ATOM	756	CB	LEU	A	186	2.260	-6.507	-7.997	1.00	15.00	A	C
ATOM	757	CG	LEU	A	186	2.504	-6.026	-9.430	1.00	15.00	A	C
ATOM	758	CD1	LEU	A	186	3.235	-7.093	-10.232	1.00	15.00	A	C
ATOM	759	CD2	LEU	A	186	1.192	-5.655	-10.104	1.00	15.00	A	C
ATOM	760	C	LEU	A	186	1.136	-6.180	-5.786	1.00	15.00	A	C
ATOM	761	O	LEU	A	186	-0.064	-6.428	-5.696	1.00	15.00	A	O
ATOM	762	N	GLU	A	187	2.016	-6.493	-4.836	1.00	15.00	A	N
ATOM	763	HN	GLU	A	187	2.959	-6.261	-4.964	1.00	15.00	A	H
ATOM	764	CA	GLU	A	187	1.611	-7.171	-3.608	1.00	15.00	A	C
ATOM	765	CB	GLU	A	187	2.837	-7.693	-2.853	1.00	15.00	A	C
ATOM	766	CG	GLU	A	187	2.522	-8.719	-1.774	1.00	15.00	A	C
ATOM	767	CD	GLU	A	187	1.872	-9.976	-2.322	1.00	15.00	A	C
ATOM	768	OE1	GLU	A	187	1.072	-10.596	-1.589	1.00	15.00	A	O
ATOM	769	OE2	GLU	A	187	2.162	-10.340	-3.482	1.00	15.00	A	O
ATOM	770	C	GLU	A	187	0.781	-6.248	-2.718	1.00	15.00	A	C
ATOM	771	O	GLU	A	187	-0.172	-6.688	-2.072	1.00	15.00	A	O
ATOM	772	N	VAL	A	188	1.149	-4.966	-2.689	1.00	15.00	A	N
ATOM	773	HN	VAL	A	188	1.928	-4.682	-3.212	1.00	15.00	A	H
ATOM	774	CA	VAL	A	188	0.425	-3.977	-1.896	1.00	15.00	A	C
ATOM	775	CB	VAL	A	188	1.212	-2.648	-1.777	1.00	15.00	A	C
ATOM	776	CG1	VAL	A	188	0.372	-1.566	-1.112	1.00	15.00	A	C
ATOM	777	CG2	VAL	A	188	2.500	-2.863	-0.994	1.00	15.00	A	C
ATOM	778	C	VAL	A	188	-0.953	-3.719	-2.508	1.00	15.00	A	C
ATOM	779	O	VAL	A	188	-1.918	-3.451	-1.797	1.00	15.00	A	O
ATOM	780	N	GLN	A	189	-1.036	-3.826	-3.835	1.00	15.00	A	N
ATOM	781	HN	GLN	A	189	-0.231	-4.049	-4.346	1.00	15.00	A	H
ATOM	782	CA	GLN	A	189	-2.291	-3.613	-4.550	1.00	15.00	A	C
ATOM	783	CB	GLN	A	189	-2.018	-3.433	-6.051	1.00	15.00	A	C
ATOM	784	CG	GLN	A	189	-3.235	-3.039	-6.880	1.00	15.00	A	C
ATOM	785	CD	GLN	A	189	-4.033	-4.236	-7.372	1.00	15.00	A	C
ATOM	786	OE1	GLN	A	189	-3.476	-5.300	-7.647	1.00	15.00	A	O
ATOM	787	NE2	GLN	A	189	-5.348	-4.068	-7.474	1.00	15.00	A	N
ATOM	788	HE21	GLN	A	189	-5.724	-3.195	-7.232	1.00	15.00	A	H
ATOM	789	HE22	GLN	A	189	-5.889	-4.824	-7.787	1.00	15.00	A	H
ATOM	790	C	GLN	A	189	-3.272	-4.767	-4.315	1.00	15.00	A	C
ATOM	791	O	GLN	A	189	-4.486	-4.559	-4.283	1.00	15.00	A	O
ATOM	792	N	LYS	A	190	-2.737	-5.978	-4.141	1.00	15.00	A	N
ATOM	793	HN	LYS	A	190	-1.761	-6.072	-4.168	1.00	15.00	A	H
ATOM	794	CA	LYS	A	190	-3.557	-7.172	-3.918	1.00	15.00	A	C
ATOM	795	CB	LYS	A	190	-2.691	-8.434	-3.937	1.00	15.00	A	C
ATOM	796	CG	LYS	A	190	-2.079	-8.768	-5.287	1.00	15.00	A	C
ATOM	797	CD	LYS	A	190	-1.096	-9.919	-5.161	1.00	15.00	A	C

ATOM	798	CE	LYS	A	190	-0.249	-10.075	-6.412	1.00	15.00	A	C
ATOM	799	NZ	LYS	A	190	0.838	-11.075	-6.218	1.00	15.00	A	N
ATOM	800	HZ1	LYS	A	190	0.431	-12.008	-6.002	1.00	15.00	A	H
ATOM	801	HZ2	LYS	A	190	1.451	-10.787	-5.428	1.00	15.00	A	H
ATOM	802	HZ3	LYS	A	190	1.414	-11.150	-7.080	1.00	15.00	A	H
ATOM	803	C	LYS	A	190	-4.337	-7.117	-2.603	1.00	15.00	A	C
ATOM	804	O	LYS	A	190	-5.378	-7.761	-2.470	1.00	15.00	A	O
ATOM	805	N	SER	A	191	-3.837	-6.349	-1.634	1.00	15.00	A	N
ATOM	806	HN	SER	A	191	-3.009	-5.850	-1.798	1.00	15.00	A	H
ATOM	807	CA	SER	A	191	-4.495	-6.236	-0.331	1.00	15.00	A	C
ATOM	808	CB	SER	A	191	-3.533	-5.688	0.729	1.00	15.00	A	C
ATOM	809	OG	SER	A	191	-3.219	-4.328	0.490	1.00	15.00	A	O
ATOM	810	HG	SER	A	191	-2.876	-4.229	-0.401	1.00	15.00	A	H
ATOM	811	C	SER	A	191	-5.776	-5.396	-0.392	1.00	15.00	A	C
ATOM	812	O	SER	A	191	-6.553	-5.369	0.563	1.00	15.00	A	O
ATOM	813	N	PHE	A	192	-5.990	-4.718	-1.518	1.00	15.00	A	N
ATOM	814	HN	PHE	A	192	-5.330	-4.777	-2.241	1.00	15.00	A	H
ATOM	815	CA	PHE	A	192	-7.178	-3.885	-1.709	1.00	15.00	A	C
ATOM	816	CB	PHE	A	192	-6.834	-2.658	-2.559	1.00	15.00	A	C
ATOM	817	CG	PHE	A	192	-5.948	-1.652	-1.883	1.00	15.00	A	C
ATOM	818	CD1	PHE	A	192	-6.495	-0.559	-1.234	1.00	15.00	A	C
ATOM	819	CD2	PHE	A	192	-4.571	-1.792	-1.906	1.00	15.00	A	C
ATOM	820	CE1	PHE	A	192	-5.685	0.375	-0.618	1.00	15.00	A	C
ATOM	821	CE2	PHE	A	192	-3.755	-0.862	-1.291	1.00	15.00	A	C
ATOM	822	CZ	PHE	A	192	-4.314	0.223	-0.646	1.00	15.00	A	C
ATOM	823	C	PHE	A	192	-8.291	-4.668	-2.405	1.00	15.00	A	C
ATOM	824	O	PHE	A	192	-9.431	-4.208	-2.485	1.00	15.00	A	O
ATOM	825	N	VAL	A	193	-7.950	-5.853	-2.903	1.00	15.00	A	N
ATOM	826	HN	VAL	A	193	-7.030	-6.169	-2.784	1.00	15.00	A	H
ATOM	827	CA	VAL	A	193	-8.902	-6.700	-3.622	1.00	15.00	A	C
ATOM	828	CB	VAL	A	193	-8.180	-7.514	-4.724	1.00	15.00	A	C
ATOM	829	CG1	VAL	A	193	-9.171	-8.211	-5.643	1.00	15.00	A	C
ATOM	830	CG2	VAL	A	193	-7.249	-6.619	-5.528	1.00	15.00	A	C
ATOM	831	C	VAL	A	193	-9.646	-7.658	-2.682	1.00	15.00	A	C
ATOM	832	O	VAL	A	193	-10.687	-8.211	-3.040	1.00	15.00	A	O
ATOM	833	N	SER	A	194	-9.122	-7.834	-1.473	1.00	15.00	A	N
ATOM	834	HN	SER	A	194	-8.307	-7.348	-1.230	1.00	15.00	A	H
ATOM	835	CA	SER	A	194	-9.729	-8.741	-0.496	1.00	15.00	A	C
ATOM	836	CB	SER	A	194	-8.703	-9.143	0.565	1.00	15.00	A	C
ATOM	837	OG	SER	A	194	-7.575	-9.764	-0.028	1.00	15.00	A	O
ATOM	838	HG	SER	A	194	-7.718	-9.854	-0.973	1.00	15.00	A	H
ATOM	839	C	SER	A	194	-10.985	-8.168	0.173	1.00	15.00	A	C
ATOM	840	O	SER	A	194	-11.582	-8.818	1.033	1.00	15.00	A	O
ATOM	841	N	ASP	A	195	-11.392	-6.965	-0.222	1.00	15.00	A	N
ATOM	842	HN	ASP	A	195	-10.888	-6.492	-0.917	1.00	15.00	A	H
ATOM	843	CA	ASP	A	195	-12.575	-6.331	0.361	1.00	15.00	A	C
ATOM	844	CB	ASP	A	195	-12.264	-4.891	0.776	1.00	15.00	A	C

```
ATOM    845  CG  ASP A 195     -11.266  -4.816   1.914  1.00 15.00      A    C
ATOM    846  OD1 ASP A 195     -11.692  -4.914   3.084  1.00 15.00      A    O
ATOM    847  OD2 ASP A 195     -10.059  -4.663   1.635  1.00 15.00      A    O
ATOM    848  C   ASP A 195     -13.772  -6.358  -0.591  1.00 15.00      A    C
ATOM    849  O   ASP A 195     -14.680  -5.532  -0.481  1.00 15.00      A    O
ATOM    850  N   GLU A 196     -13.780  -7.320  -1.511  1.00 15.00      A    N
ATOM    851  HN  GLU A 196     -13.036  -7.957  -1.543  1.00 15.00      A    H
ATOM    852  CA  GLU A 196     -14.868  -7.443  -2.480  1.00 15.00      A    C
ATOM    853  CB  GLU A 196     -14.311  -7.734  -3.880  1.00 15.00      A    C
ATOM    854  CG  GLU A 196     -13.336  -6.696  -4.409  1.00 15.00      A    C
ATOM    855  CD  GLU A 196     -13.176  -6.769  -5.916  1.00 15.00      A    C
ATOM    856  OE1 GLU A 196     -14.085  -6.298  -6.632  1.00 15.00      A    O
ATOM    857  OE2 GLU A 196     -12.143  -7.294  -6.380  1.00 15.00      A    O
ATOM    858  C   GLU A 196     -15.860  -8.546  -2.102  1.00 15.00      A    C
ATOM    859  O   GLU A 196     -16.843  -8.766  -2.811  1.00 15.00      A    O
ATOM    860  N   VAL A 197     -15.615  -9.233  -0.986  1.00 15.00      A    N
ATOM    861  HN  VAL A 197     -14.836  -9.000  -0.440  1.00 15.00      A    H
ATOM    862  CA  VAL A 197     -16.489 -10.333  -0.565  1.00 15.00      A    C
ATOM    863  CB  VAL A 197     -15.713 -11.674  -0.499  1.00 15.00      A    C
ATOM    864  CG1 VAL A 197     -16.649 -12.861  -0.683  1.00 15.00      A    C
ATOM    865  CG2 VAL A 197     -14.596 -11.714  -1.534  1.00 15.00      A    C
ATOM    866  C   VAL A 197     -17.175 -10.078   0.786  1.00 15.00      A    C
ATOM    867  O   VAL A 197     -18.036 -10.853   1.204  1.00 15.00      A    O
ATOM    868  N   ILE A 198     -16.812  -8.992   1.461  1.00 15.00      A    N
ATOM    869  HN  ILE A 198     -16.141  -8.388   1.082  1.00 15.00      A    H
ATOM    870  CA  ILE A 198     -17.397  -8.685   2.769  1.00 15.00      A    C
ATOM    871  CB  ILE A 198     -16.378  -8.007   3.708  1.00 15.00      A    C
ATOM    872  CG1 ILE A 198     -15.767  -6.769   3.039  1.00 15.00      A    C
ATOM    873  CG2 ILE A 198     -15.294  -8.993   4.117  1.00 15.00      A    C
ATOM    874  CD1 ILE A 198     -14.916  -5.924   3.963  1.00 15.00      A    C
ATOM    875  C   ILE A 198     -18.657  -7.820   2.681  1.00 15.00      A    C
ATOM    876  O   ILE A 198     -19.273  -7.516   3.702  1.00 15.00      A    O
ATOM    877  N   VAL A 199     -19.050  -7.440   1.469  1.00 15.00      A    N
ATOM    878  HN  VAL A 199     -18.544  -7.736   0.683  1.00 15.00      A    H
ATOM    879  CA  VAL A 199     -20.228  -6.590   1.286  1.00 15.00      A    C
ATOM    880  CB  VAL A 199     -19.886  -5.315   0.490  1.00 15.00      A    C
ATOM    881  CG1 VAL A 199     -19.049  -4.368   1.336  1.00 15.00      A    C
ATOM    882  CG2 VAL A 199     -19.161  -5.665  -0.802  1.00 15.00      A    C
ATOM    883  C   VAL A 199     -21.389  -7.313   0.607  1.00 15.00      A    C
ATOM    884  O   VAL A 199     -22.176  -6.691  -0.110  1.00 15.00      A    O
ATOM    885  N   SER A 200     -21.515  -8.613   0.848  1.00 15.00      A    N
ATOM    886  HN  SER A 200     -20.879  -9.054   1.450  1.00 15.00      A    H
ATOM    887  CA  SER A 200     -22.589  -9.394   0.243  1.00 15.00      A    C
ATOM    888  CB  SER A 200     -22.190  -9.889  -1.153  1.00 15.00      A    C
ATOM    889  OG  SER A 200     -22.140  -8.821  -2.088  1.00 15.00      A    O
ATOM    890  HG  SER A 200     -22.902  -8.866  -2.673  1.00 15.00      A    H
ATOM    891  C   SER A 200     -23.010 -10.564   1.129  1.00 15.00      A    C
```

```
ATOM    892  O    SER A 200     -23.300 -11.654   0.633  1.00 15.00       A    O
ATOM    893  N    THR A 201     -23.060 -10.334   2.438  1.00 15.00       A    N
ATOM    894  HN   THR A 201     -22.837  -9.442   2.781  1.00 15.00       A    H
ATOM    895  CA   THR A 201     -23.458 -11.376   3.375  1.00 15.00       A    C
ATOM    896  CB   THR A 201     -22.245 -12.183   3.916  1.00 15.00       A    C
ATOM    897  OG1  THR A 201     -21.068 -11.362   3.963  1.00 15.00       A    O
ATOM    898  HG1  THR A 201     -21.048 -10.776   3.205  1.00 15.00       A    H
ATOM    899  CG2  THR A 201     -21.975 -13.418   3.062  1.00 15.00       A    C
ATOM    900  C    THR A 201     -24.277 -10.798   4.537  1.00 15.00       A    C
ATOM    901  O    THR A 201     -23.696 -10.283   5.492  1.00 15.00       A    O
ATOM    902  N    ASN A 202     -25.629 -10.900   4.407  1.00 15.00       A    N
ATOM    903  HN   ASN A 202     -25.974 -11.334   3.596  1.00 15.00       A    H
ATOM    904  CA   ASN A 202     -26.633 -10.414   5.395  1.00 15.00       A    C
ATOM    905  CB   ASN A 202     -26.048  -9.723   6.639  1.00 15.00       A    C
ATOM    906  CG   ASN A 202     -25.920 -10.668   7.823  1.00 15.00       A    C
ATOM    907  OD1  ASN A 202     -25.803 -11.884   7.653  1.00 15.00       A    O
ATOM    908  ND2  ASN A 202     -25.926 -10.112   9.032  1.00 15.00       A    N
ATOM    909 HD21  ASN A 202     -26.002  -9.136   9.091  1.00 15.00       A    H
ATOM    910 HD22  ASN A 202     -25.883 -10.704   9.817  1.00 15.00       A    H
ATOM    911  C    ASN A 202     -27.700  -9.537   4.745  1.00 15.00       A    C
ATOM    912  O    ASN A 202     -27.394  -8.532   4.103  1.00 15.00       A    O
ATOM    913  CAT  UNK B   1     -15.066  -1.512   2.893  1.00 15.00       B    C
ATOM    914  CBG  UNK B   1     -15.597  -1.373   4.171  1.00 15.00       B    C
ATOM    915  CBF  UNK B   1     -15.527  -0.148   4.824  1.00 15.00       B    C
ATOM    916  CBK  UNK B   1     -14.926   0.940   4.199  1.00 15.00       B    C
ATOM    917  CAV  UNK B   1     -14.395   0.801   2.921  1.00 15.00       B    C
ATOM    918  CAQ  UNK B   1     -14.467  -0.424   2.269  1.00 15.00       B    C
ATOM    919  SAA  UNK B   1     -13.810  -0.597   0.655  1.00 15.00       B    S
ATOM    920  OAD  UNK B   1     -13.309   0.733   0.175  1.00 15.00       B    O
ATOM    921  OAF  UNK B   1     -12.676  -1.579   0.675  1.00 15.00       B    O
ATOM    922  NAJ  UNK B   1     -14.980  -1.134  -0.346  1.00 15.00       B    N
ATOM    923  HAB  UNK B   1     -14.568  -1.556  -1.153  1.00 15.00       B    H
ATOM    924  CAL  UNK B   1     -15.860  -0.026  -0.743  1.00 15.00       B    C
ATOM    925  CAN  UNK B   1     -17.109  -0.567  -1.444  1.00 15.00       B    C
ATOM    926  CAP  UNK B   1     -18.154   0.293  -1.761  1.00 15.00       B    C
ATOM    927  CBA  UNK B   1     -19.293  -0.199  -2.387  1.00 15.00       B    C
ATOM    928  CAX  UNK B   1     -19.386  -1.551  -2.699  1.00 15.00       B    C
ATOM    929  CAY  UNK B   1     -18.342  -2.411  -2.379  1.00 15.00       B    C
ATOM    930  CAU  UNK B   1     -17.203  -1.919  -1.753  1.00 15.00       B    C
ATOM    931  NAI  UNK B   1     -16.249   0.766   0.435  1.00 15.00       B    N
ATOM    932  HAA  UNK B   1     -17.219   0.622   0.634  1.00 15.00       B    H
ATOM    933  CAM  UNK B   1     -16.015   2.039   0.128  1.00 15.00       B    C
ATOM    934  CAR  UNK B   1     -16.362   3.113   0.939  1.00 15.00       B    C
ATOM    935  CAS  UNK B   1     -16.928   2.886   2.189  1.00 15.00       B    C
ATOM    936  CBD  UNK B   1     -17.245   3.957   3.015  1.00 15.00       B    C
ATOM    937  CBB  UNK B   1     -16.997   5.258   2.592  1.00 15.00       B    C
ATOM    938  CBE  UNK B   1     -16.433   5.486   1.342  1.00 15.00       B    C
```

```
ATOM    939  CAW UNK B   1     -16.117   4.414   0.517  1.00 15.00      B    C
ATOM    940  CAK UNK B   1     -15.308   2.158  -1.062  1.00 15.00      B    C
ATOM    941  OAC UNK B   1     -14.892   3.210  -1.542  1.00 15.00      B    O
ATOM    942  NAH UNK B   1     -15.166   0.955  -1.606  1.00 15.00      B    N
ATOM    943  SAB UNK B   1     -14.379   0.643  -2.999  1.00 15.00      B    S
ATOM    944  OAE UNK B   1     -12.969   1.143  -2.897  1.00 15.00      B    O
ATOM    945  OAG UNK B   1     -15.072   1.347  -4.126  1.00 15.00      B    O
ATOM    946  CAO UNK B   1     -14.363  -1.078  -3.316  1.00 15.00      B    C
ATOM    947  CAZ UNK B   1     -15.340  -1.651  -4.122  1.00 15.00      B    C
ATOM    948  CBI UNK B   1     -15.328  -3.021  -4.358  1.00 15.00      B    C
ATOM    949  CBJ UNK B   1     -14.340  -3.817  -3.789  1.00 15.00      B    C
ATOM    950  CBH UNK B   1     -13.362  -3.244  -2.985  1.00 15.00      B    C
ATOM    951  CBC UNK B   1     -13.374  -1.873  -2.750  1.00 15.00      B    C
END
```

PROCHECK

Ramachandran Plot
yog3

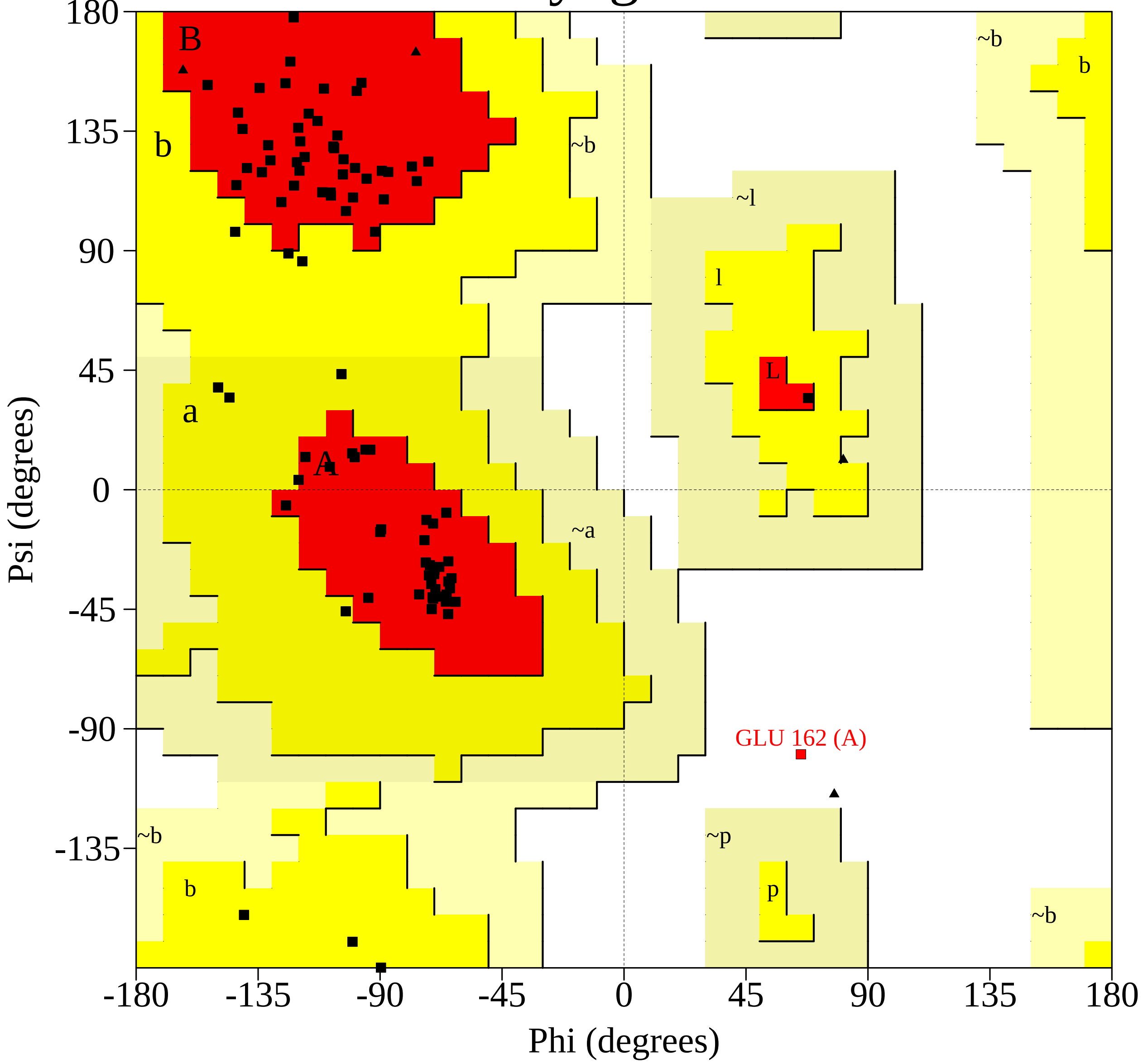

Plot statistics

Residues in most favoured regions [A,B,L]	78	86.7%
Residues in additional allowed regions [a,b,l,p]	11	12.2%
Residues in generously allowed regions [~a,~b,~l,~p]	0	0.0%
Residues in disallowed regions	1	1.1%
	----	------
Number of non-glycine and non-proline residues	90	100.0%
Number of end-residues (excl. Gly and Pro)	3	
Number of glycine residues (shown as triangles)	4	
Number of proline residues	1	

Total number of residues	98	

Based on an analysis of 118 structures of resolution of at least 2.0 Angstroms and R-factor no greater than 20%, a good quality model would be expected to have over 90% in the most favoured regions.

yog3_01.ps

Ramachandran plots for all residue types

yog3

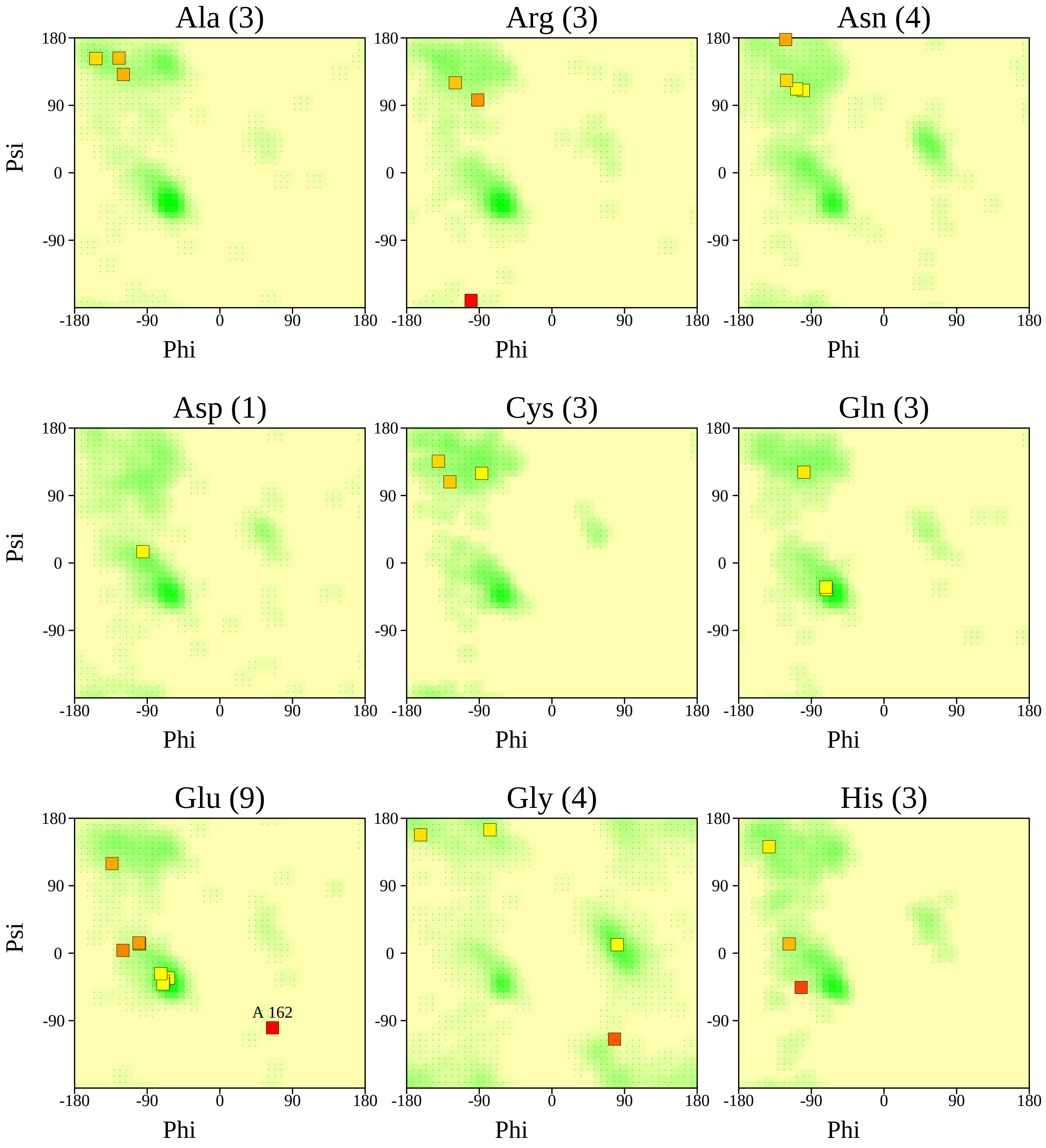

Numbers of residues are shown in brackets. Those in unfavourable conformations (score < -3.00) are labelled.
Shading shows favourable conformations as obtained from an analysis of 163 structures at resolution 2.0A or better.

Ramachandran plots for all residue types

yog3

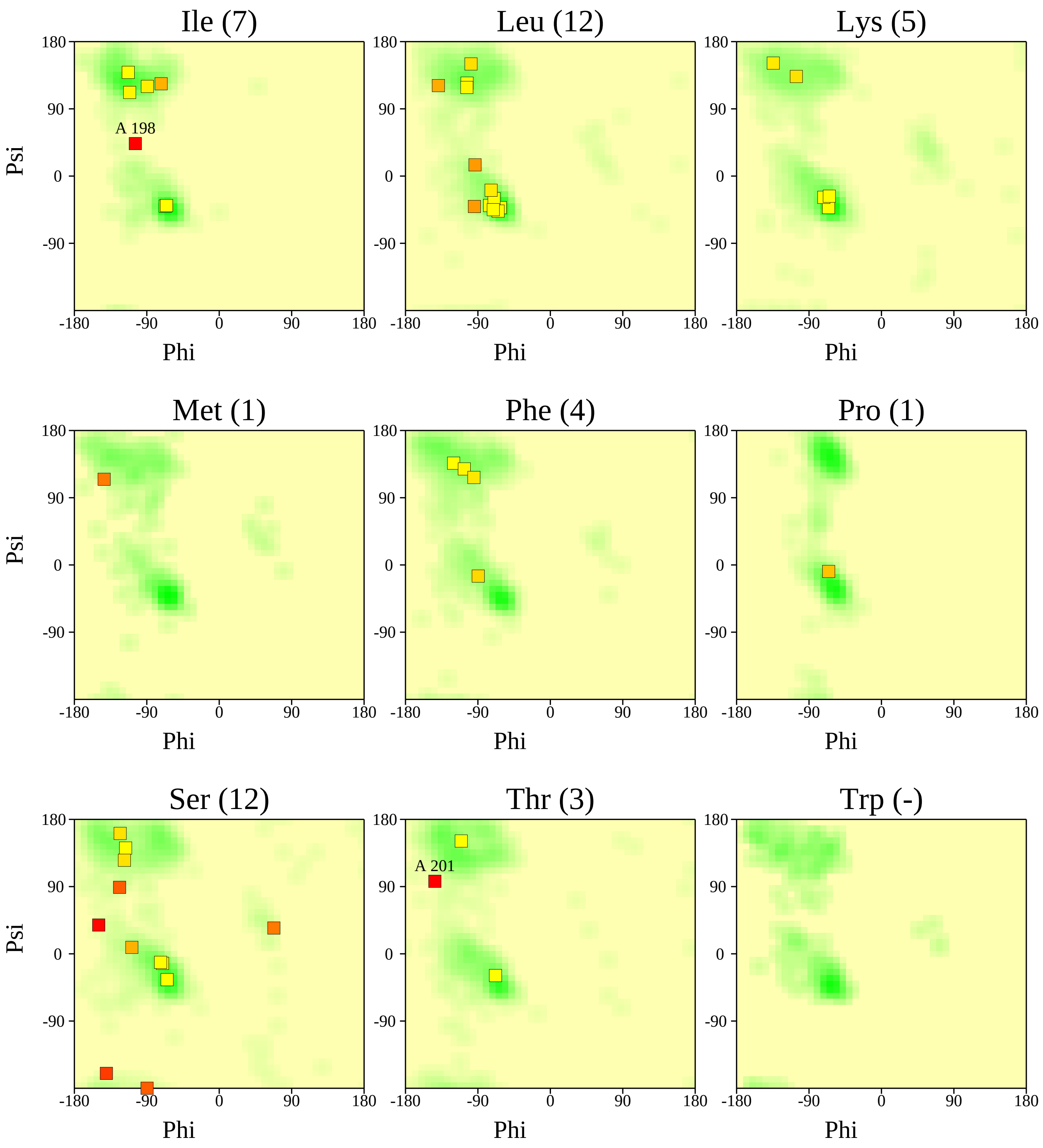

Numbers of residues are shown in brackets. Those in unfavourable conformations (score < -3.00) are labelled.
Shading shows favourable conformations as obtained from an analysis of 163 structures at resolution 2.0A or better.

Ramachandran plots for all residue types

yog3

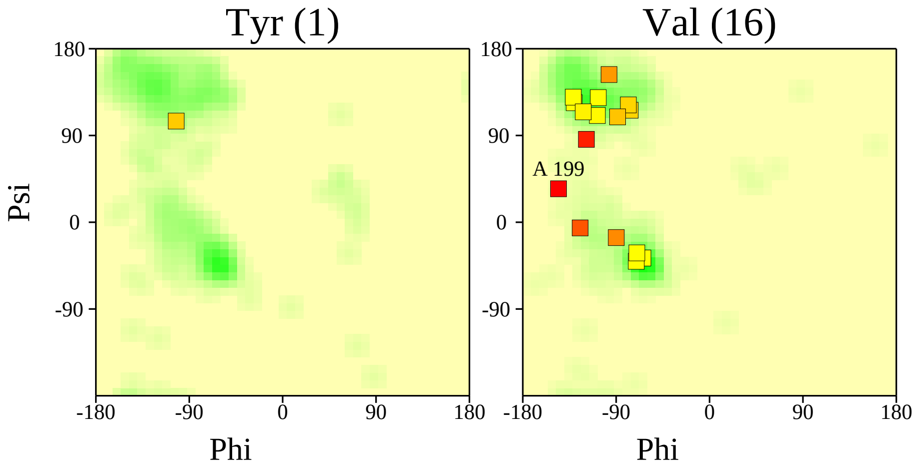

Numbers of residues are shown in brackets. Those in unfavourable conformations (score < -3.00) are labelled.
Shading shows favourable conformations as obtained from an analysis of 163 structures at resolution 2.0A or better.

Chi1-Chi2 plots

yog3

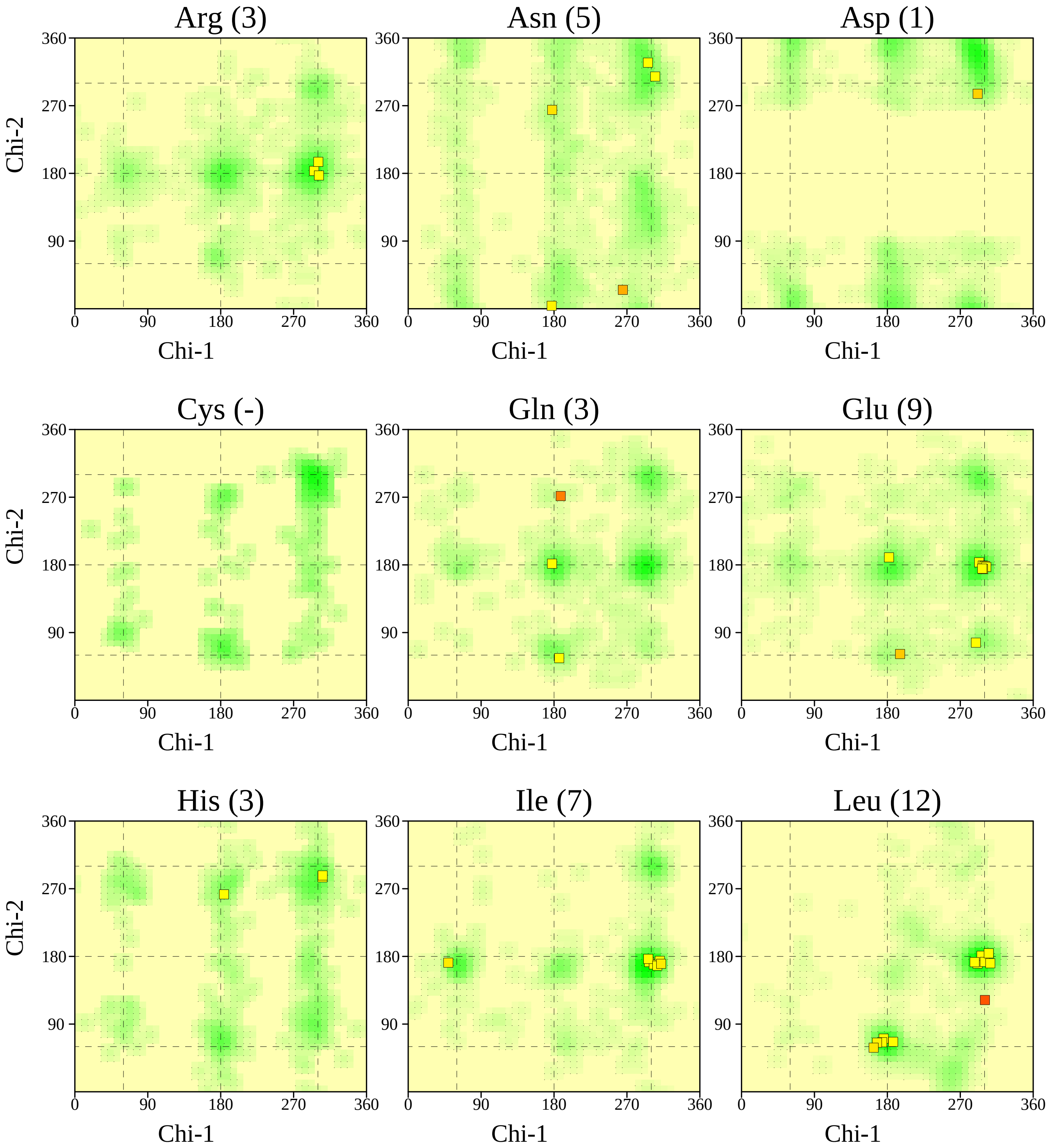

Numbers of residues are shown in brackets. Those in unfavourable conformations (score < -3.00) are labelled.
Shading shows favourable conformations as obtained from an analysis of 163 structures at resolution 2.0A or better.

Chi1-Chi2 plots

yog3

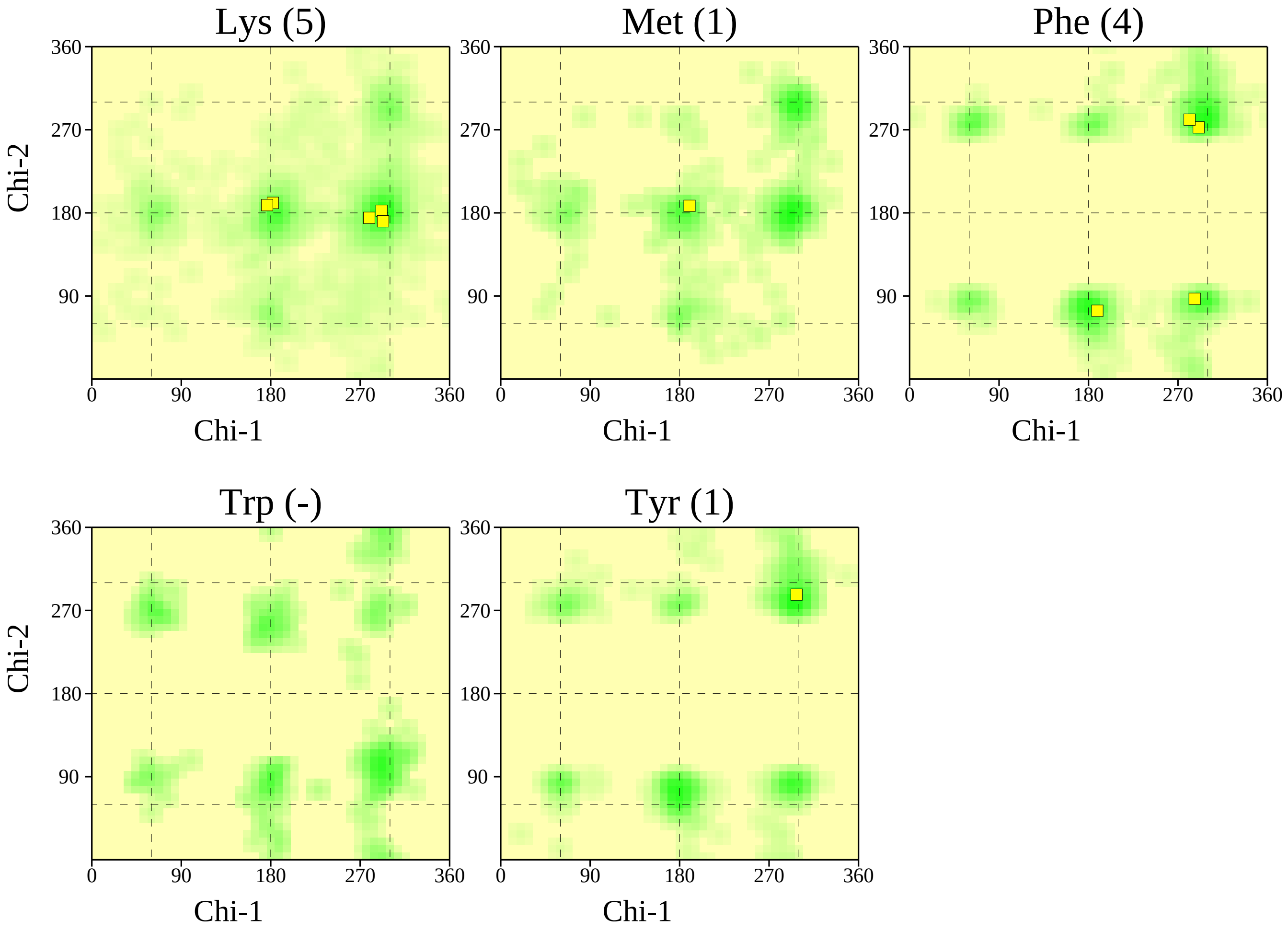

Numbers of residues are shown in brackets. Those in unfavourable conformations (score < -3.00) are labelled.
Shading shows favourable conformations as obtained from an analysis of 163 structures at resolution 2.0A or better.

PROCHECK

Main-chain parameters
yog3

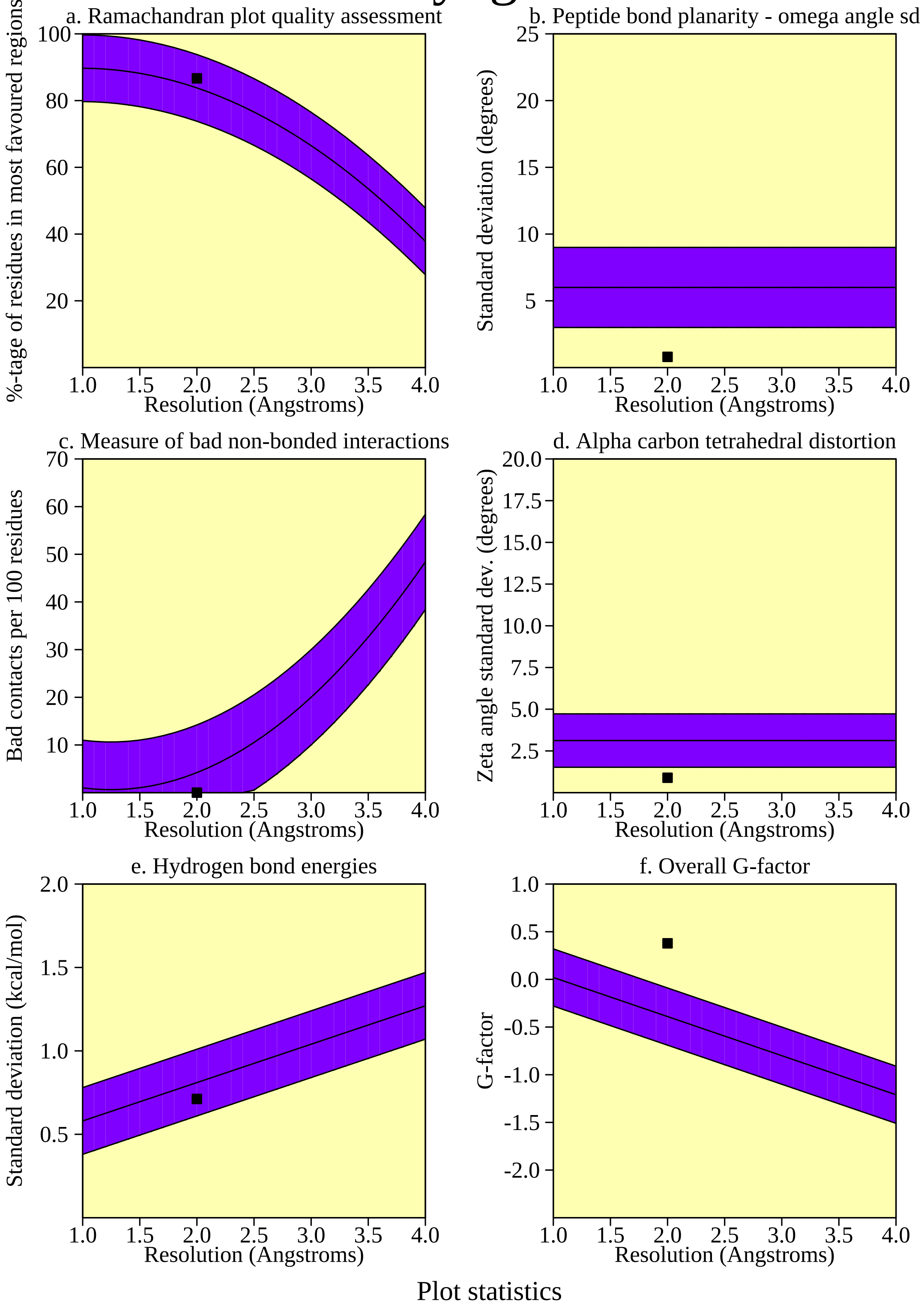

Plot statistics

Stereochemical parameter	No. of data pts	Parameter value	Comparison values Typical value	Band width	No. of band widths from mean	
a. %-tage residues in A, B, L	90	86.7	83.8	10.0	0.3	Inside
b. Omega angle st dev	96	0.8	6.0	3.0	-1.7	BETTER
c. Bad contacts / 100 residues	0	0.0	4.2	10.0	-0.4	Inside
d. Zeta angle st dev	93	0.9	3.1	1.6	-1.4	BETTER
e. H-bond energy st dev	68	0.7	0.8	0.2	-0.5	Inside
f. Overall G-factor	98	0.4	-0.4	0.3	2.6	BETTER

PROCHECK

Side-chain parameters

yog3

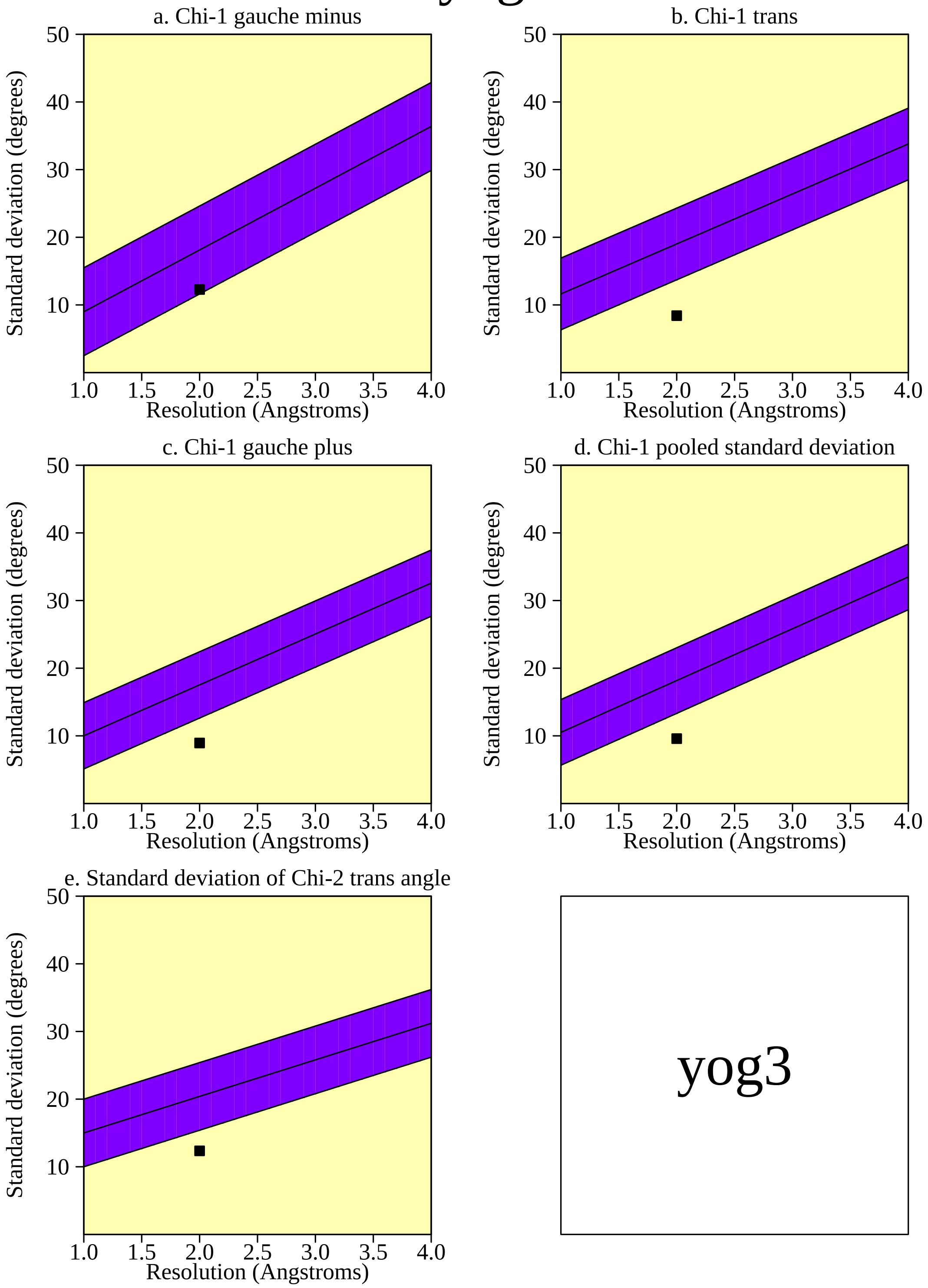

Plot statistics

Stereochemical parameter	No. of data pts	Parameter value	Comparison values Typical value	Band width	No. of band widths from mean	
a. Chi-1 gauche minus st dev	10	12.3	18.1	6.5	-0.9	Inside
b. Chi-1 trans st dev	35	8.4	19.0	5.3	-2.0	BETTER
c. Chi-1 gauche plus st dev	44	8.9	17.5	4.9	-1.8	BETTER
d. Chi-1 pooled st dev	89	9.6	18.2	4.8	-1.8	BETTER
e. Chi-2 trans st dev	31	12.4	20.4	5.0	-1.6	BETTER

Main-chain bond lengths

yog3

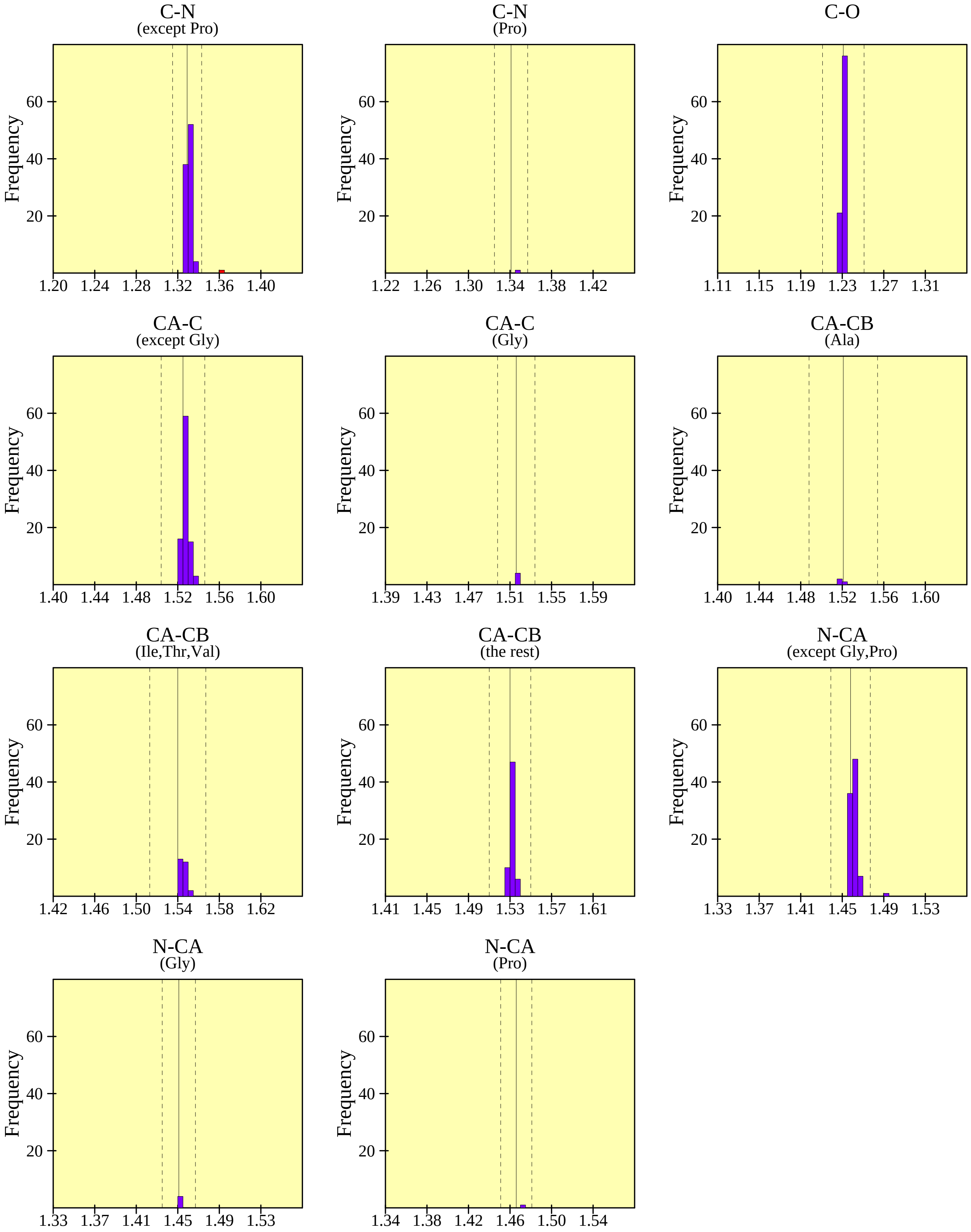

Black bars > 2.0 st. devs. from mean.

Solid and dashed lines represent the mean and standard deviation values as per Engh & Huber small-molecule data.
